# Convenient and Efficient Synthesis of Functionalized 2-Sulfenylindoles

**DOI:** 10.3390/ma13204492

**Published:** 2020-10-10

**Authors:** Justyna Doroszuk, Mateusz Musiejuk, Bartosz Jędrzejewski, Juliusz Walczak, Dariusz Witt

**Affiliations:** Department of Organic Chemistry, Faculty of Chemistry, Gdansk University of Technology, Narutowicza 11/12, 80-233 Gdansk, Poland; juswiecz@student.pg.gda.pl (J.D.); mateusz.musiejuk@interia.eu (M.M.); barjedrz@student.pg.edu.pl (B.J.); pyrher@gmail.com (J.W.)

**Keywords:** indoles, sulfides, thiotosylates, sulfenylation, sulfenylindoles

## Abstract

A simple, efficient, and practical sulfenylation at the C2 position of *N*-tosylindoles under mild conditions was developed. The designed transformation is based on the reaction of *N*-tosylindoles with BuLi and *S*-alkyl, and *S*-aryl phosphorodithioates or thiotosylates to produce 2-sulfenylindoles in moderate to high yields. The presence of additional hydroxy, carboxy, or amino functionalities did not disturb the formation of products.

## 1. Introduction

Indole is a structural functionality found in a diversity of biologically active molecules [1]. The functionalization of an indole ring by a variety of substituents and methods has been intensively studied [2,3,4]. These indole derivatives were used as blue-light emitting materials [5], materials for solar cells [6], potential prodrugs [7], and the dual-specificity tyrosine phosphorylation-regulated kinase 1A (DYRK1A) inhibitors [8].

**Figure 1:**
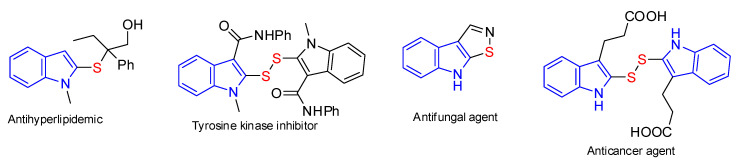
Selected examples of biologically active sulfenylindoles.

Sulfenylindoles, as important derivatives of indoles, can be frequently found in medicinal and bioorganic chemistry. Selected examples are presented in Figure 1.

They were applied in drug discovery and development for the treatment of various diseases. Compounds for the treatment of cancer [9], HIV [10,11], vascular [12], heart disease [13], respiratory disorders [14], and allergies [15] were developed. These compounds were also applied as COX-2 inhibitors in medicinal chemistry [16,17] and potent inhibitors of tubulin polymerization [18,19].

**Scheme 1:**
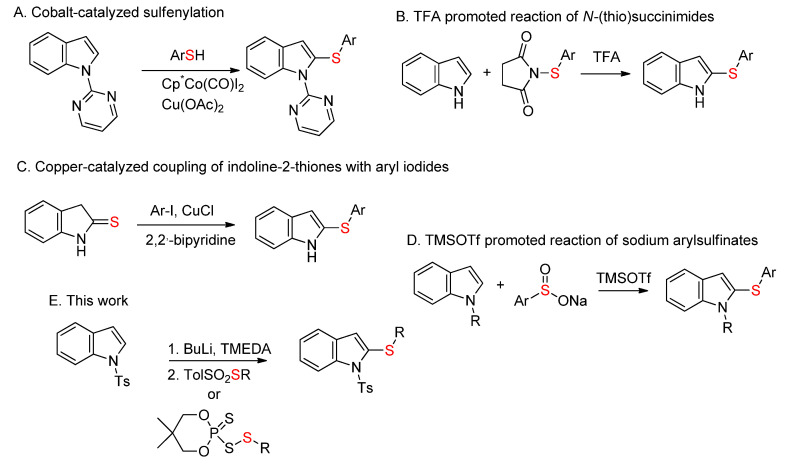
General strategies for the synthesis of 2-sulfenylindoles (**A**–**D**) and our new approach (**E**).

There are many efficient methodologies for the synthesis of sulfenylindoles reported in the literature [20,21,22,23,24,25]. Among them, the direct sulfenylation of indoles is one of the most efficient and common strategies [26,27,28,29,30,31,32,33]. Due to the nucleophilic properties of an indole ring, it can be easily functionalized at the C3 position by using a variety of electrophiles. The formation of carbon–carbon bonds and carbon–heteroatom bonds can be observed according to the structure of electrophiles. The 3-Sulfenylindoles are readily available by the reaction of indoles with a variety of sulfenylating reagents. The most commonly utilized reagents are sulfenyl halides [34], thiols [35], disulfides [36,37,38], arylsulfonyl hydrazides [39,40], arylsulfonyl chlorides [41,42], sulfinic acids [43], and sulfonium salts [44,45]. Usually, sulfenylation of indoles proceeds at the C3 position of an indole ring. In contrast, sulfenylation at the C2 position of indoles is difficult but has also been accomplished. The most common strategies involve introducing a directing group at the N1 position (Scheme 1A), [46] blocking the C3 position, [47] using a N-(thio)succinimide/trifluoroacetic acid (TFA) reaction system (Scheme 1B) [48], and removing the proton at the C2 position [49,50,51,52].

Other practical procedures involve the reaction of copper-catalyzed coupling of indoline-2-thiones with aryl iodides (Scheme 1C) [53], acid-catalyzed rearrangement of 3-sulfenylindole [54,55], and multicomponent synthesis of thieno[2,3-b]indole derivatives [56,57,58]. Recently, regioselective sulfenylation at the C2 position of indoles with sodium arylsulfinates has been accomplished in the presence of TMSOTf as a promoter (Scheme 1D) [59].

Although the presented methods provided 2-sulfenylated indoles, the applied harsh conditions were incompatible with various functional groups. Moreover, in most cases, the arylsulfenyl group can be introduced in the C2 position. Thus, the development of an efficient and versatile strategy for the synthesis of 2-sulenylindoles possessing alkylsulfenyl and arylsulfenyl functionalities with additional functional groups is highly desirable. In this context, we report a regioselective sulfenylation at the C2 position of *N*-tosylindoles with sulfenylating reagents (Scheme 1E). Earlier studies demonstrated the application of readily available 5,5-dimethyl-2-thioxo-1,3,2-dioxaphosphorinane-2-disulfanyl derivatives for the preparation of functionalized unsymmetrical disulfides [60,61,62,63,64], *α*-sulfenylated carbonyl compounds [65], and unsymmetrical alkynyl sulfides [66,67].

## 2. Materials and Methods

All 1-((4-methylphenyl)sulfonyl)indoles (**1a**–**d**) were obtained from indole, 5-methoxyindole, 5-bromoindole, and 5-aminoindole, respectively. Sodium hydride and *p*-toluenesulfonyl chloride were purchased from Merck (Darmstadt, Germany). All bromides required for preparation thiotosylates **3** were purchased from ProChimia (Sopot, Poland). The 5,5-Dimethyl-2-thioxo-1,3,2-dioxaphosphorinane-2-disulfanyl derivatives [60,61,62,63,64,65] **2** and thiotosylates [67] **3** were prepared by the literature methods. Sodium 4-methylbenzenesulfonothioate was obtained from sodium 4-methylbenzenesulfinate purchased from Merck (Darmstadt, Germany). *N*,*N*,*N*’,*N*’-tetramethylethylenediamine (TMEDA) is available from Merck (Darmstadt, Germany). Tetrahydrofuran was pre-dried over KOH pellets and distilled. Subsequently, tetrahydrofurane (THF) was dried by heating under reflux over potassium in the presence of benzophenone as an indicator and distilled. Thin layer chromatography (TLC) was performed with silica gel Supelco UV254 (St. Louis, MI, USA). Column chromatography was performed using silica gel 60 (230–400 mesh, Merck, Darmstadt, Germany). NMR spectra were recorded on Brucker 400 MHz spectrometers. The residual solvent peak was used as the internal reference (CDCl_3_: δ = 7.26 ppm for ^1^H, δ = 77.0 ppm for ^13^C). IR spectra were recorded on Nicolet Is50 Fourier-transform infrared (FT-IR) spectrometer (Wien, Austria) by Attenuated Total Reflectance (ATR) method. Melting points were measured with Gallenkamp 7936B apparatus (Warwick, UK).

### 2.1. General Procedure for the Preparation of 2-sulfenyl-1-((4-methylphenyl)sulfonyl)-1H-indoles ***8*** from Phosphorodithioates ***2***

To a stirred, cooled to 0 °C solution of indole **1a**–**d** (1 mmol) and *N,N,N′,N′-*tetramethylethane-1,2-diamine (1 mmol) in dry THF (5 mL) was added dropwise *n*-BuLi (2.5 M in hexane; 1 mmol). The mixture was stirred at 0 °C for 5 min. Then the solution of phosphorodithioate disulfanyl derivative **2** (1 mmol) in dry THF (5 mL) was added. The reaction was warmed to room temperature and stirred for 15 min. The mixture was diluted with Et_2_O (30 mL) and washed with water (10 mL), dried over MgSO_4_, filtered and concentrated under vacuum. The products were purified by column chromatography.

### 2.2. General Procedure for the Preparation of 2-sulfenyl-1-((4-methylphenyl)sulfonyl)-1H-indoles ***8*** from Thiotosylates ***3***

To a stirred, cooled to 0 °C solution of indole **1a**–**d** (1 mmol) and *N,N,N′,N′-*tetramethylethane-1,2-diamine (1 mmol) in dry THF (5 mL) was added dropwise *n*-BuLi (2.5 M in hexane; 1 mmol). The mixture was stirred in 0 °C for 5 min. Then the solution of thiotosylates **3** (1 mmol) in dry THF (5 mL) was added. The reaction was warmed to room temperature and stirred for 15 min. The mixture was diluted with Et_2_O (30 mL) and washed with water (10 mL), dried over MgSO_4_, filtered, and concentrated under vacuum. The products were purified by column chromatography.

Synthesis of starting materials and 2-sulfenylindoles **8** with analytical data and copy of IR, and NMR spectra are in the Supplementary Materials.

## 3. Results and Discussion

The preliminary determination of the conditions was performed on *N*-tosylindole **1a** to obtain the required 2-sulfenylindole **8**. The sulfenylation at the C2 position of indoles was investigated, employing various electrophilic sulfenylating compounds **2**–**7** to determine the most efficient reagents. The results are summarized in Table 1.

By using didodecyl disulfide **4** and diphenyl disulfide **7**, expected products **8a** and **8b** were produced in 72% and 95% yields, respectively. It appeared that diaryl disulfides were more effective than dialkyl disulfides in this synthesis (Table 1; entries 3 and 6). Disulfide **7** is probably the better electrophile due to the better leaving group properties of the aryl thiolate anion. However, the main disadvantage of disulfides is the introduction of only half of a starting material into the product.

When **1a** was treated with *N*-(thiododecyl)phthalimide **5** or *N*-(thiododecyl)succinimide **6**, indole **8a** was obtained in 55% and 53% yield, respectively (Table 1; entries 4 and 5). Although *N*-(thiododecyl)phthalimide or *N*-(thiododecyl)succinimide are known as good electrophilic reagents, it looks like leaving groups, phthalimide or succinimide anions, are less efficient for examined transformation. The best yield was observed by using *S*-dodecyl toluenethiosulfonate **3a** or 1-((5,5-dimethyl-2-thioxo-1,3,2-dioxaphosphorinan-2-yl)disulfanyl) dodecane **2a** as the sulfenylating reagent, with which **8a** was obtained in 83% and 72% yields, respectively (Table 1; entries 1 and 2). From a practical point of view, corresponding thiosulfonates and 5,5-dimethyl-2-thioxo-1,3,2-dioxaphosphorinane-2-disulfanyl derivatives are readily available from thiols or appropriate alkyl halides and were selected as sulfenylating reagents for further studies. In addition, THF was the best solvent to accomplish sulfenylation at the C2 position. *N*,*N*,*N*′,*N*′-Tetramethylethane-1,2-diamine (TMEDA) was added to avoid the aggregation of regioselective [49] generated 2-lithium-*N*-tosylindoles and to improve their reactivity.

When the most efficient reagent and a useful set of conditions were determined, then we examined the scope of the reaction by subjecting indole derivatives **1a**–**d** to the sulfenylation at the C2 position by a variety of phosphorodithioate disulfanyl derivatives **2**. The results are summarized in Table 2.

As shown in Table 2, the formation of 2-sulfenylated indoles **8** was accomplished when the indole ring was not substituted (Table 2; entries 1–9) in 65–94% yields. The presence of an electron-donating or an electron-withdrawing group at the C5 position did not disturb the progress of the reaction, and appropriate indoles **8** were obtained in 69–91% yields (Table 2; entries 10–23). When the protected amino group was attached in the position C5 of an indole ring, then 2-sulfenylated indoles **8** were obtained in lower yields 65–78%. The formation of products **8** can be accomplished for alkyl and aryl derivatives **2**. Moreover, the presence of additional functional groups did not affect reactivity, and functionalized 2-sulfenyl indoles were produced in good yields.

Although the above reactions were successful, we were interested in that they improved the formation of 2-sulfenylated indoles **8** by using thiotosylates **3** as a sulfenylating reagent. We selected indoles **1a**–**d** and appropriate thiotosylates **3** with the same substitution pattern to clarify the effect of the sulfenylating reagent. The results are summarized in Table 3.

As demonstrated, the expected 2-sulfenylindoles **8** were obtained in good yields independent of the substitution pattern of the indole ring. When the indole ring was not substituted at the C5 position, the yield of 2-sulfenylindoles **8a**–**8i** was in the range 68–92% (Table 3; entries 1–9). The presence of an electron-donating group at the C5 position did not disturb the reaction and 2-sulfenylindoles **8j**–**8p** were obtained in 67–82% yields (Table 3; entries 10–16). The substitution of the indole ring at the C5 position by an electron-withdrawing substituent did not affect the reactivity, and the corresponding 2-sulfenylindoles **8r**–**8y** were produced in good yields (68–86%) (Table 3; entries 17–23). However, when the protected amino group was present in the C5 position, a slightly lower yield (62–79%) of products **8z**–**8af** was observed (Table 3; entries 24–30).

Interestingly, the developed sulfenylation method can be applied to either *S*-alkyl thiotosylates, or *S*-aryl thiotosylates. The presence of additional functional groups, such as ester, cyano, hydroxy, and protected amino moieties or carbon–carbon double bonds, did not disturb the formation of 2-sulfenylindoles **8**. However, using thiotosylates **3** instead of phosphorodithioate disulfanyl derivatives **2** as a sulfenylating reagent did not improve the yield of product **8**. Although both methods provided 2-sulfenylindoles **8** in comparable yields and tolerance of additional functional groups, the using of thiotosylates **3** seems to be more convenient. These compounds are readily available from the reaction of appropriate alkyl halides and sodium thiotosylate or thiols and tosyl bromide.

**Figure 2:**
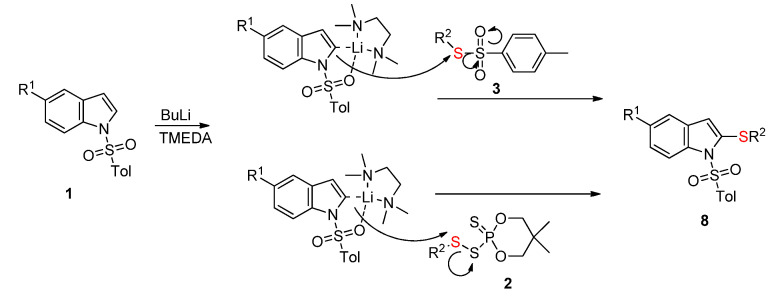
Plausible reaction mechanism.

The suggested mechanism to explain the course of the reactions involves the initial regioselective formation of the 2-lithium-*N*-tosylindoles through the reaction of BuLi with *N*-tosylindoles **1**. The resulting lithium salt reacts with electrophilic phosphorodithioate disulfanyl derivatives **2** or thiotosylates **3** to yield 2-sulfenylindoles **8** (Figure 2).

**Scheme 2:**
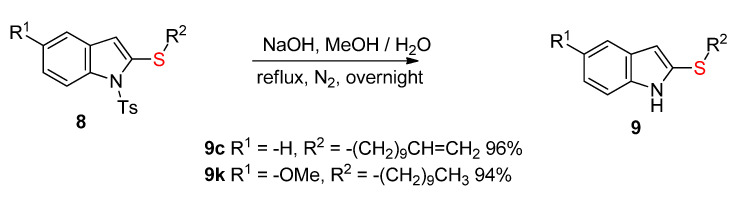
Formation of nonprotected indoles **9**.

The tosyl group protects the indole functionality and is also used as directing metalation group [68] to produce 2-lithium-*N*-tosylindoles. The regioselective proton removal is responsible for exclusive 2-sulfenylated *N*-tosylindole **8** formation. Although a variety of protected compounds **8** were obtained, we were interested in using them as starting materials for the preparation of nonprotected indoles. The standard basic conditions for tosyl group removal were applied [69]. Selected compounds **8c** and **8k** were treated with NaOH in MeOH/H_2_O, and the resulting mixture was refluxed overnight under nitrogen (Scheme 2).

As demonstrated, the formation of nonprotected indole **9** was convenient and effective. When 2-sulfenylindoles **8c** and **8k** were treated with NaOH in methanol (conditions were not optimized), nonprotected products **9c** and **9k** were obtained in 96% and 94% yields, respectively. This deprotection method, together with the developed regioselective sulfenylation of *N*-tosylindoles **1** at the C2 position, gives an attractive alternative for the preparation of 3-unsubstituted nonprotected 2-sulfenylindoles.

## 4. Conclusions

In summary, we developed a convenient, efficient, and versatile regioselective sulfenylation of 3-unsubstituted *N*-tosylindoles **1** to access a variety of new functionalized indole derivatives. These methods were accomplished with readily available phosphorodithioate disulfanyl derivatives **2** or thiotosylates **3** as the sulfenylating reagents, and regioselectively generated 2-lithium-*N*-tosylindoles. Moreover, developed methods can be applied to *S*-alkyl and *S*-aryl derivatives of **2** and **3**. The reactions were accomplished under mild conditions and the presence of various functional groups did not disturb the formation of 2-sulfenylindoles **8**.

## Figures and Tables

**Table 1:** Evaluation of the reaction conditions.

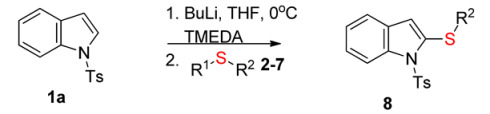			
**Entry ^1^**	**R ^1^-S-R ^2^**	**8**	**Yield (%) ^2^**
1	**2a**	**8a**	72
2	**3a**	**8a**	83
3	**4**	**8a**	72
4	**5**	**8a**	55
5	**6**	**8a**	53
6	**7**	**8b**	95
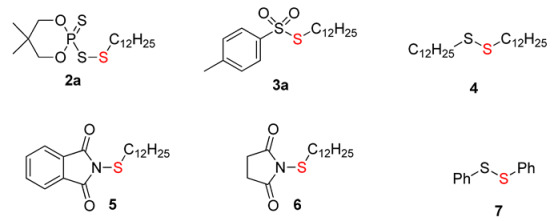			

^1^ Reaction conditions: **1a** (1 mmol), TMEDA (1 mmol), BuLi (1 mmol), 0 °C, THF, 5 min. Then, **2**–**7** (1 mmol) was added, rt, 15 min. ^2^ Isolated yields.

**Table 2:** Reaction of indoles **1** with phosphorodithioate disulfanyl derivatives **2**.

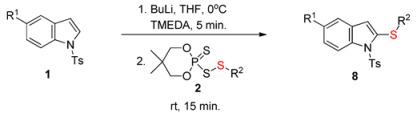				
**Entry ^1^**	**R ^1^**	**R ^2^**	**8**	**Yield (%) ^2^**
1	H– **1a**	CH_3_(CH_2_)_11_–	**8a**	72
2		C_6_H_5_–	**8b**	94
3		CH_2_=CH(CH_2_)_9_–	**8c**	78
4		CH_3_(CH_2_)_9_–	**8d**	78
5		4–CH_3_–C_6_H_4_–	**8e**	86
6		CH_3_OOC(CH_2_)_10_–	**8f**	75
7		HO(CH_2_)_11_–	**8g**	67
8		4–CN–C_6_H_4_–CH_2_–	**8h**	65
9		C_6_H_5_CH_2_–	**8i**	87
10	CH_3_O– **1b**	CH_2_=CH(CH_2_)_9_–	**8j**	71
11		CH_3_(CH_2_)_9_–	**8k**	81
12		4–CH_3_–C_6_H_4_–	**8l**	86
13		CH_3_OOC(CH_2_)_10_–	**8m**	72
14		HO(CH_2_)_11_–	**8n**	78
15		4–CN–C_6_H_4_–CH_2_–	**8o**	72
16		C_6_H_5_CH_2_–	**8p**	82
17	Br– **1c**	CH_2_=CH(CH_2_)_9_–	**8r**	69
18		CH_3_(CH_2_)_9_–	**8s**	84
19		4–CH_3_–C_6_H_4_–	**8t**	91
20		CH_3_OOC(CH_2_)_10_–	**8u**	79
21		HO(CH_2_)_11_–	**8w**	76
22		4–CN–C_6_H_4_–CH_2_–	**8x**	69
23		C_6_H_5_CH_2_–	**8y**	85
24	BocNH– **1d**	CH_2_=CH(CH_2_)_9_–	**8z**	65
25		CH_3_(CH_2_)_9_–	**8aa**	68
26		4–CH_3_–C_6_H_4_–	**8ab**	72
27		CH_3_OOC(CH_2_)_10_–	**8ac**	65
28		HO(CH_2_)_11_–	**8ad**	71
29		4–CN–C_6_H_4_–CH_2_–	**8ae**	66
30		C_6_H_5_CH_2_–	**8af**	78

^1^ Reaction conditions: **1** (1 mmol), TMEDA (1 mmol), BuLi (1 mmol or 2 mmol for **1d**), 0 °C, THF, 5 min. Then, **2** (1 mmol) was added, rt, 15 min. ^2^ Isolated yields.

**Table 3:** Reaction of indoles **1** with thiotosylates **3**.

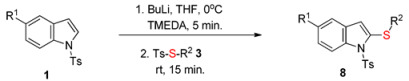				
**Entry ^1^**	**R ^1^**	**R ^2^**	**8**	**Yield (%) ^2^**
1	H– **1a**	CH_3_(CH_2_)_11_–	**8a**	83
2		C_6_H_5_–	**8b**	92
3		CH_2_=CH(CH_2_)_9_–	**8c**	81
4		CH_3_(CH_2_)_9_–	**8d**	82
5		4–CH_3_–C_6_H_4_–	**8e**	82
6		CH_3_OOC(CH_2_)_10_–	**8f**	69
7		HO(CH_2_)_11_–	**8g**	68
8		4–CN–C_6_H_4_–CH_2_–	**8h**	69
9		C_6_H_5_CH_2_–	**8i**	85
10	CH_3_O– **1b**	CH_2_=CH(CH_2_)_9_–	**8j**	78
11		CH_3_(CH_2_)_9_–	**8k**	81
12		4–CH_3_–C_6_H_4_–	**8l**	82
13		CH_3_OOC(CH_2_)_10_–	**8m**	67
14		HO(CH_2_)_11_–	**8n**	76
15		4–CN–C_6_H_4_–CH_2_–	**8o**	68
16		C_6_H_5_CH_2_–	**8p**	82
17	Br– **1c**	CH_2_=CH(CH_2_)_9_–	**8r**	74
18		CH_3_(CH_2_)_9_–	**8s**	83
19		4–CH_3_–C_6_H_4_–	**8t**	86
20		CH_3_OOC(CH_2_)_10_–	**8u**	78
21		HO(CH_2_)_11_–	**8w**	76
22		4–CN–C_6_H_4_–CH_2_–	**8x**	68
23		C_6_H_5_CH_2_–	**8y**	84
24	BocNH– **1d**	CH_2_=CH(CH_2_)_9_–	**8z**	70
25		CH_3_(CH_2_)_9_–	**8aa**	79
26		4–CH_3_–C_6_H_4_–	**8ab**	71
27		CH_3_OOC(CH_2_)_10_–	**8ac**	62
28		HO(CH_2_)_11_–	**8ad**	69
29		4–CN–C_6_H_4_–CH_2_–	**8ae**	63
30		C_6_H_5_CH_2_–	**8af**	78

^1^ Reaction conditions: **1** (1 mmol), TMEDA (1 mmol), BuLi (1 mmol or 2 mmol for **1d**), 0 °C, THF, 5 min. Then, **3** (1 mmol) was added, rt, 15 min. ^2^ Isolated yields.

## Supplementary Materials

The following are available online at https://www.mdpi.com/1996-1944/13/20/4492/s1: Synthesis of starting materials and 2-sulfenylindoles 8 with analytical data and copy of IR, and NMR spectra.
