# Supplementary material for: Convenient and Efficient Synthesis of Functionalized 2-Sulfenylindoles
Source: Materials (Basel). 2020 Oct 10;13(20):4492. doi: 10.3390/ma13204492 (PMC7601631; doi:10.3390/ma13204492)
Supplement: Supplementary file 1 [file materials-13-04492-s001.pdf]

materials

Supplementary Information

Convenient and Efficient Synthesis of Functionalized 2-Sulfenylindoles

Abstract: A simple, efficient, and practical sulfenylation at the C2 position of *N*-tosylindoles under mild conditions was developed. The designed transformation is based on the reaction of *N*-tosylindoles with BuLi and *S*-alkyl and *S*-aryl thiotosylates to produce 2-sulfenylindoles in moderate to high yields. The presence of additional hydroxy, carboxy, or amino functionalities did not disturb the formation of products.

Table of Contents

1	General information	3
2	Typical procedure for the preparation of sodium 4-methylbenzenesulfonothioate.	3
3	General procedure for the preparation of thiotosylates derivatives **3** and representative analytical data	3
4	General procedure for the preparation of 1-[(4-methylphenyl)sulfonyl]indoles **1a–d** and representative analytical data	4
5	General procedure for the preparation of 2-sulfenyl-1-[(4-methylphenyl)sulfonyl]-1*H*-indoles **8a–af** from phosphorodithioate disulfanyl derivatives **2**.	5
6	General procedure for the preparation of 2-sulfenyl-1-[(4-methylphenyl)sulfonyl]-1*H*-indoles **8a–af** from thiotosylates **3** and representative analytical data.	6
7	General procedure for the preparation of 2-(dodecyl-1-sulfenyl)-1-[(4-methylphenyl)sulfonyl]-1*H*-indole **8a** and representative analytical data	19
8	General procedure for preparation of 2-sulfenyl-1*H*-indoles **9c** and **9k** and representative analytical data	20
9	NMR and IR spectra	22

1. General Information

All 1-[(4-methylphenyl)sulfonyl]indoles (**1a–d**) were obtained from indole, 5-methoxyindole, 5-bromoindole, and 5-aminoindole, respectively. Sodium hydride and *p*-toluenesulfonyl chloride were purchased from Merck. All bromides required for preparation thiotosylates **3** were purchased from ProChimia. 5,5-Dimethyl-2-thioxo-1,3,2-dioxaphosphorinane-2-disulfanyl derivatives [*RSC Adv.*, **2016**, *6*, 105449–105453] **2** and thiotosylates [*Eur. J. Org. Chem.* **2018**, 6333–6337] **3** were prepared by the literature methods. Sodium 4-methylbenzenesulfonothioate was obtained from sodium 4-methylbenzenesulfinate purchased from Merck. *N*,*N*,*N*′,*N*′-tetramethylethylenediamine (TMEDA) is available from Merck. Tetrahydrofuran was pre-dried over KOH pellets and distilled. Subsequently, THF was dried by heating under reflux over potassium in the presence of benzophenone as an indicator and distilled. TLC was performed with silica gel Polygram SIL G/UV254. Column chromatography was performed using silica gel 60 (230–400 mesh, Merck). NMR spectra were recorded on Brucker 400 MHz spectrometers. The residual solvent peak was used as the internal reference ($CDCl_3$: δ = 7.26 ppm for ^1H, δ = 77.0 ppm for ^{13}C). IR spectra were recorded on Nicolet Is50 Ft-IR spectrometer by ATR method. Melting points were measured with a Gallenkamp 7936B apparatus.

2. Typical Procedure for the Preparation of Sodium 4-methylbenzenesulfonothioate

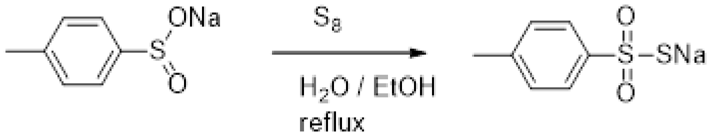

To a stirred solution of sodium 4-methylbenzenesulfinate (112 mmol) in H_2O/EtOH (100 mL/100 mL) was added sulfur (122 mmol). Mixture was warmed to reflux for 8 h. Then residual sulfur was removed by filtration. Filtrate was evaporated. The wet solid was dried under vacuum for 9 h at room temperature to provide 18.8 g (80%) of sodium 4-methylbenzenesulfonotioate (procedure from L. Wang, D.L.J. Clive, *Org. Synth.* 2013, **90**, 10–24).

3. General Procedure for the Preparation of Thiotosylates Derivatives 3 and Representative Analytical Data

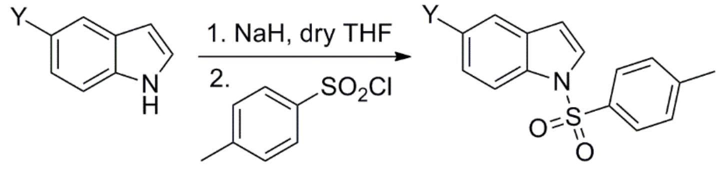

1a-d

Y: **1a** H-
1b MeO-
1c Br-
1d BocNH-

To a stirred solution of alkyl bromide (1 eq) in acetonitrile (20 mL) was added sodium 4-methylbenzene-sulfonotioate (1.1 eq). Reaction was stirred for 3 h under reflux. Then mixture was slowly cooled to room temperature and stirred overnight. After evaporation of the solvent under reduced pressure, the residue was dissolved in Et_2O and washed with water, dried over $MgSO_4$, filtered and concentrated under vacuum. The products were purified by column chromatography (procedure from J. Doroszuk, M. Musiejuk, Ł. Ponikiewski, D. Witt, *Eur. J. Org. Chem.* **2018**, 6333–6337, doi:10.1002/ejoc.201801181).

4. General Procedure for the Preparation of 1-[(4-methylphenyl)sulfonyl]indoles (1a-d) and Representative Analytical Data

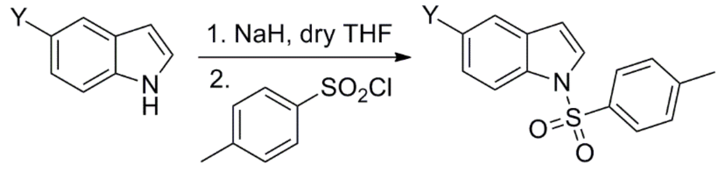

1a-d

Y: **1a** H-
1b MeO-
1c Br-
1d BocNH-

To a stirred, cooled to 0 °C suspension of NaH (1.5 mmol) in dry THF (5 mL) was added dropwise a solution of indole (1 mmol) in dry THF (5 mL). Mixture was stirred at 0 °C for 15 min. Then the solution of *p*-toluenesulfonyl chloride (1.1 mmol) in dry THF (5 mL) was added slowly. Mixture was warm to room temperature and stirred overnight. Then reaction mixture was diluted with EtOAc (25 mL) and washed with water (10 mL), dried over $MgSO_4$, filtered and concentrated under vacuum. The product was purified by column chromatography.

1-[(4-methylphenyl)sulfonyl]-1H-indole 1a

Chromatography: Petroleum ether-toluene (4:1), R_f = 0.3, a yellow solid mp: 84.6–85.2 °C, yield: 0.219 g (81%):

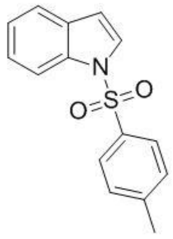

^{1}H NMR (400 MHz, $CDCl_3$) δ 8.01 (d, *J* = 9.1 Hz, 1H), 7.79 (d, *J* = 8.4 Hz, 2H), 7.59 (d, *J* = 3.7 Hz, 1H), 7.55 (d, *J* = 7.8 Hz, 1H), 7.35 – 7.31 (m, 1H), 7.25 (dd, *J* = 6.0, 1.7 Hz, 2H), 7.24–7.22 (m, 1H), 6.68 (d, *J* = 3.7 Hz, 1H), 2.36 (s, 3H).

^{13}C NMR (101 MHz, $CDCl_3$) δ 144.9, 135.4, 134.9, 130.8, 129.9, 126.8, 126.3, 124.5, 123.3, 121.4, 113.5, 109.0, 21.6; signals: 13 expected, 13 observed.

5-methoxy-1-[(4-methylphenyl)sulfonyl]-1H-indole 1b

Chromatography: Toluene, R_f = 0.4, a white solid mp: 108.3–108.6 °C, yield: 0.262 g (87%):

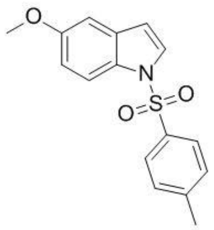

^{1}H NMR (400 MHz, $CDCl_3$) δ 7.92–7.88 (m, 1H), 7.77–7.73 (m, 2H), 7.54 (d, *J* = 3.6 Hz, 1H), 7.23 (dd, *J* = 8.6, 0.6 Hz, 2H), 6.99–6.93 (m, 2H), 6.60 (dd, *J* = 3.6, 0.7 Hz, 1H), 3.83 (s, 3H), 2.35 (s, 3H).

^{13}C NMR (101 MHz, $CDCl_3$) δ 156.4, 144.8, 135.3, 131.8, 129.8, 129.6, 127.1, 126.7, 114.4, 113.7, 109.2, 103.6, 55.6, 21.5; signals: 14 expected, 14 observed

5-bromo-1-[(4-methylphenyl)sulfonyl]-1H-indole 1c

Chromatography: Petroleum ether-Dichloromethane (4:1), R_f = 0.3, a white solid mp: 133.2–133.5 °C, yield: 0.318 g (91%):

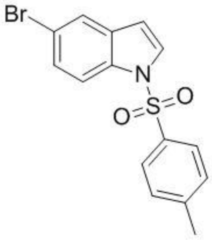

^{1}H NMR (400 MHz, $CDCl_3$) δ 7.88 (d, *J* = 8.8 Hz, 1H), 7.78–7.74 (m, 2H), 7.68 (d, *J* = 1.7 Hz, 1H), 7.58 (d, *J* = 3.7 Hz, 1H), 7.44–7.40 (m, 1H), 7.25 (dd, *J* = 8.6, 0.6 Hz, 2H), 6.61 (dd, *J* = 3.7, 0.7 Hz, 1H), 2.37 (s, 3H).

^{13}C NMR (101 MHz, $CDCl_3$) δ 145.3, 135.0, 133.5, 132.5, 130, 127.6, 127.5, 126.8, 124.0, 116.8, 115, 108.3, 21.6; signals: 13 expected, 13 observed.

5-[[(1,1-dimethylethoxy)carbonyl]amino]-1-[(4-methylphenyl)sulfonyl]-1H-indole 1d

Chromatography: Petroleum ether-Dichloromethane (4:1), R_f = 0.2, a white solid mp: 138.9–139.3 °C, yield: 0.332 g (86%)

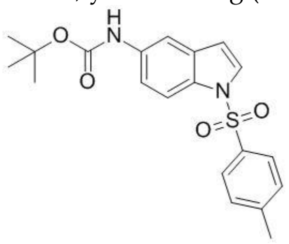

^{1}H NMR (400 MHz, $CDCl_3$) δ 8.21 (s, 1H), 7.71 (s, 1H), 7.29 (d, *J* = 4.3 Hz, 2H), 7.20–7.11 (m, 2H), 6.50 (s, 2H), 1.57 (s, 12H).

^{13}C NMR (101 MHz, $CDCl_3$) δ 153.1, 144.9, 135.2, 134.2, 131.5, 131.2, 129.8, 127.2, 126.7, 116.9, 113.9, 111.3, 109.4, 80.5, 28.4, 21.5; signals: 16 expected, 16 observed.

5. General Procedure for the Preparation of 2-sulfenyl-1-[(4-methylphenyl)sulfonyl]-1*H*-indoles 8 from Phosphorodithioate Disulfanyl Derivatives 2

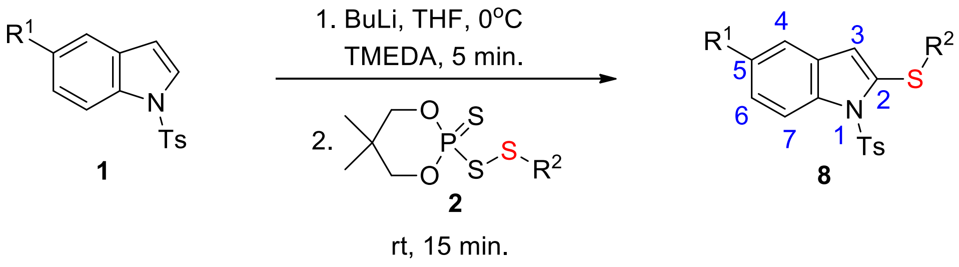

To a stirred, cooled to 0 °C solution of indole **1a–d** (1 mmol) and *N,N,N′,N′*-tetramethylethane-1,2-diamine (1 mmol) in dry THF (5 mL) was added dropwise *n*-BuLi (2.5 M in hexane; 1 mmol*). Mixture was stirred in 0 °C for 5 min. Then the solution of phosphorodithioate disulfanyl derivative **2** (1 mmol) in dry THF (5 mL) was added. Reaction was warmed to room temperature and stirred for 15 min. Mixture was diluted with Et_2O (30 mL) and washed with water (10 mL), dried over $MgSO_4$, filtered and concentrated under vacuum. The products were purified by column chromatography.
*in case of indole **1d** (1 mmol), *n*-BuLi (2 mmol) must be added

Table S1. Reaction of indoles **1** with phosphorodithioate disulfanyl derivatives **2**.

Entry	R^1	R^2	Yield[b] [%]
1	H **1a**	$CH_3(CH_2)_{11}$–	72 **8a**
2		C_6H_5–	94 **8b**
3		CH_2=$CH(CH_2)_9$–	78 **8c**
4		$CH_3(CH_2)_9$–	78 **8d**
5		4–CH_3–C_6H_4–	86 **8e**
6		$H_3COOC(CH_2)_{10}$–	75 **8f**
7		$HO(CH_2)_{11}$–	67 **8g**
8		4–CN–C_6H_4–CH_2–	65 **8h**
9		$C_6H_5CH_2$–	87 **8i**
10	CH_3O– **1b**	CH_2=$CH(CH_2)_9$–	71 **8j**
11		$CH_3(CH_2)_9$–	81 **8k**
12		4–CH_3–C_6H_4–	86 **8l**
13		$H_3COOC(CH_2)_{10}$–	72 **8m**
14		$HO(CH_2)_{11}$–	78 **8n**
15		4–CN–C_6H_4–CH_2–	72 **8o**
16		$C_6H_5CH_2$–	82 **8p**
17	Br– **1c**	CH_2=$CH(CH_2)_9$–	69 **8r**
18		$CH_3(CH_2)_9$–	84 **8s**
19		4–CH_3–C_6H_4–	91 **8t**
20		$H_3COOC(CH_2)_{10}$–	79 **8u**
21		$HO(CH_2)_{11}$–	76 **8w**
22		4–CN–C_6H_4–CH_2–	69 **8x**
23		$C_6H_5CH_2$–	85 **8y**
24	$(CH_3)_3COC(O)NH$– **1d**	CH_2=$CH(CH_2)_9$–	65 **8z**
25		$CH_3(CH_2)_9$–	68 **8aa**
26		4–CH_3–C_6H_4–	72 **8ab**
27		$H_3COOC(CH_2)_{10}$–	65 **8ac**
28		$HO(CH_2)_{11}$–	71 **8ad**
29		4–CN–C_6H_4–CH_2–	66 **8ae**
30		$C_6H_5CH_2$–	78 **8af**

[b] isolated yields

6. General Procedure for the Preparation of 2-sulfenyl-1-[(4-methylphenyl)sulfonyl]-1*H*-indoles 8 from Thiotosylates 3 and Representative Analytical Data

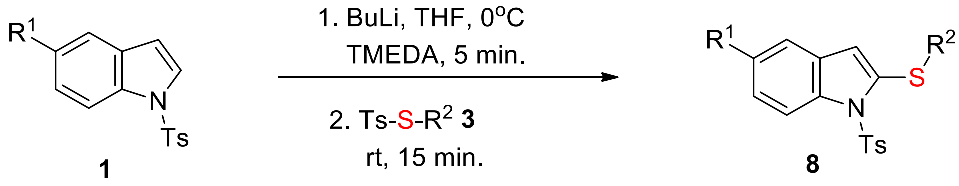

To a stirred, cooled to 0 °C solution of indole **1a–d** (1 mmol) and *N,N,N′,N′*-tetramethylethane-1,2-diamine (1 mmol) in dry THF (5 mL) was added dropwise *n*-BuLi (2.5 M in hexane; 1 mmol*). Mixture was stirred in 0 °C for 5 min. Then the solution of thiotosylates **3** (1 mmol) in dry THF (5 mL) was added. Reaction was warmed to room temperature and stirred for 15 min. Mixture was diluted with Et_2O (30 mL) and washed with water (10 mL), dried over $MgSO_4$, filtered and concentrated under vacuum. The products were purified by column chromatography. *in case of indole **1d** (1 mmol), *n*-BuLi (2 mmol) must be added

Table S2. Reaction of indoles **1** with thiotosylates **3**.

Entry	R^1	R^2	Yield[b] [%]
1		$CH_3(CH_2)_{11}$–	83 **8a**
2		C_6H_5–	92 **8b**
3		CH_2=$CH(CH_2)_9$–	81 **8c**
4		$CH_3(CH_2)_9$–	82 **8d**
5	H	4–CH_3–C_6H_4–	82 **8e**
6		$H_3COOC(CH_2)_{10}$–	69 **8f**
7		$HO(CH_2)_{11}$–	68 **8g**
8		4–CN–C_6H_4–CH_2–	69 **8h**
9		$C_6H_5CH_2$–	85 **8i**
10		CH_2=$CH(CH_2)_9$–	78 **8j**
11		$CH_3(CH_2)_9$–	81 **8k**
12		4–CH_3–C_6H_4–	82 **8l**
13	CH_3O–	$H_3COOC(CH_2)_{10}$–	67 **8m**
14		$HO(CH_2)_{11}$–	76 **8n**
15		4–CN–C_6H_4–CH_2–	68 **8o**
16		$C_6H_5CH_2$–	82 **8p**
17		CH_2=$CH(CH_2)_9$–	74 **8r**
18		$CH_3(CH_2)_9$–	83 **8s**
19		4–CH_3–C_6H_4–	86 **3t**
20	Br–	$H_3COOC(CH_2)_{10}$–	78 **8u**
21		$HO(CH_2)_{11}$–	76 **8w**
22		4–CN–C_6H_4–CH_2–	68 **8x**
23		$C_6H_5CH_2$–	84 **8y**
24		CH_2=$CH(CH_2)_9$–	70 **8z**
25		$CH_3(CH_2)_9$–	79 **8aa**
26		4–CH_3–C_6H_4–	71 **8ab**
27	$(CH_3)_3COC(O)NH$–	$H_3COOC(CH_2)_{10}$–	62 **8ac**
28		$HO(CH_2)_{11}$–	69 **8ad**
29		4–CN–C_6H_4–CH_2–	63 **8ae**
30		$C_6H_5CH_2$–	78 **8af**

[b] isolated yields

2-(Dodec-1-ylsulfanyl)-1-[(4-methylphenyl)sulfonyl]-1H-indole 8a

Chromatography: Petroleum ether (PE)-Toluene (T) (1:2), R_f = 0.5, a yellowish oil, yield: 0.340 g (72%) (Table S1); 0.392 g (83%) (Table S2).

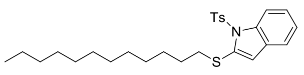

^{1}H NMR (400 MHz, $CDCl_3$) δ 8.26–8.20 (m, 1H), 7.89–7.80 (m, 2H), 7.46–7.35 (m, 1H), 7.32–7.19 (m, 4H), 6.50 (d, *J* = 0.5 Hz, 1H), 2.96 (t, *J* = 7.4 Hz, 2H), 2.36 (s, 3H), 1.75–1.64 (m, 2H), 1.49–1.40 (m, 2H), 1.37–1.24 (m, 16H), 0.92 (t, *J* = 6.8 Hz, 3H).
^{13}C NMR (101 MHz, $CDCl_3$) δ 144.8, 137.8, 135.6, 135.0, 130.0, 129.6, 127.1, 124.0, 123.6, 119.5, 114.7, 111.7, 35.1, 31.9, 29.7, 29.6, 29.6, 29.5, 29.4, 29.2, 28.9, 28.4, 22.7, 21.6, 14.1; signals: 25 expected, 25 observed.
IR (ATR) 2924 (w), 2852 (w), 1441 (m), 1373 (m), 1174 (s), 1088 (m), 677 (s), 580 (s), 542 (s).
HRMS (ESI): m/z [M + H]$^+$ calcd for $C_{27}H_{38}NO_2S_2$: 472.2338; found: 472.2342

2-[Phenylsulfanyl]-1-[(4-methylphenyl)sulfonyl]-1H-indole 8b

Chromatography: Toluene, R_f = 0.4, a white solid mp: 110–112 °C, yield: 0.357 g (94%) (Table S1); 0.349 g (92%) (Table S2).

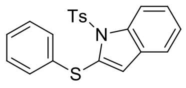

^{1}H NMR (400 MHz, $CDCl_3$) δ 8.26 (d, *J* = 8.4 Hz, 1H), 7.86 (d, *J* = 8.4 Hz, 2H), 7.57 (dd, *J* = 15.7, 5.7 Hz, 1H), 7.40–7.32 (m, 2H), 7.33–7.29 (m, 5H), 7.21 (d, *J* = 8.0 Hz, 2H), 6.47 (d, *J* = 0.6 Hz, 1H), 2.37 (s, 3H).
^{13}C NMR (101 MHz, $CDCl_3$) δ 144.9, 135.6, 131.3, 129.9, 129.6, 127.9, 127.2, 126.8, 124.7, 124.5, 123.7, 120.1, 115.4, 114.8, 113.5, 109.0, 21.6, signals: 17 expected and observed.
IR (ATR) 1444 (m), 1363 (s), 1171 (s), 1084 (m), 1005 (m), 796 (m), 752 (s), 681(s), 586 (s), 538 (s).
HRMS (ESI): m/z [M + H]$^+$ calcd for $C_{21}H_{18}NO_2S_2$: 380.0773; found: 380.0776.

2-(Undec-10-en-1-ylsulfanyl)-1-[(4-methylphenyl)sulfonyl]-1H-indole 8c

Chromatography: PE-CH_2Cl_2 (4:1), R_f = 0.5, a yellow solid mp: 62.7–63.2 °C, yield: 0.355 g (78%) (Table S1); 0.369 g (81%) (Table S2).

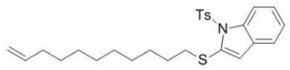

^{1}H NMR (400 MHz, $CDCl_3$) δ 8.23 (dd, *J* = 8.3, 0.9 Hz, 1H), 7.87–7.83 (m, 2H), 7.40 (ddd, *J* = 7.6, 1.4, 0.7 Hz, 1H), 7.31–7.18 (m, 4H), 6.50 (d, *J* = 0.7 Hz, 1H), 5.84 (dd, *J* = 17.0, 10.2 Hz, 1H), 5.05–4.94 (m, 2H), 2.96 (t, *J* = 7.4 Hz, 2H), 2.36 (s, 3H), 2.11–2.03 (m, 2H), 1.69 (dt, *J* = 15.0, 7.3 Hz, 2H), 1.49–1.25 (m, 12H).
^{13}C NMR (101 MHz, $CDCl_3$) δ 144.8, 139.2, 137.8, 135.6, 135.0, 130.0, 129.6, 129.0, 128.2, 127.1, 125.3, 124.0, 123.6, 119.5, 114.7, 114.2, 111.7, 35.1, 33.8, 29.4, 29.1, 28.9, 28.4, 21.6; signals: 24 observed, 24 expected.
IR (ATR) 2921 (w), 2848 (w), 1441 (m), 1371 (m), 1165 (m), 1088 (m), 746 (s), 683 (s), 584 (s), 538 (s)
HRMS (ESI): m/z [M + H]$^+$ calcd for $C_{26}H_{34}NO_2S_2$: 456.2025; found: 456.2029.

2-(Dec-1-ylsulfanyl)-1-[(4-methylphenyl)sulfonyl]-1H-indole 8d

Chromatography: PE-T (1:2), R_f = 0.5, a yellowish oil, yield: 0.346 g (78%) (Table S1); 0.364 g (82%) (Table S2).

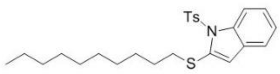

^{1}H NMR (400 MHz, $CDCl_3$) δ 8.27–8.19 (m, 1H), 7.88–7.81 (m, 2H), 7.45–7.36 (m, 1H), 7.32–7.19 (m, 4H), 6.50 (d, *J* = 0.5 Hz, 1H), 2.96 (t, *J* = 7.4 Hz, 2H), 2.36 (s, 3H), 1.74–1.65 (m, 2H), 1.49–1.40 (m, 2H), 1.38–1.23 (m, 12H), 0.92 (t, *J* = 6.8 Hz, 3H).

^{13}C NMR (101 MHz, $CDCl_3$) δ 144.8, 137.8, 135.6, 135.0, 130.0, 129.6, 127.1, 124.0, 123.6, 119.5, 114.6, 111.6, 35.1, 31.9, 29.6, 29.5, 29.3, 29.2, 28.9, 28.4, 22.7, 21.6, 14.1; signals: 23 expected, 23 observed.
IR (ATR) 2924 (w), 2852 (w), 1441 (m), 1373 (m), 1174 (s), 1088 (m), 677 (s), 580 (s), 542 (s).
HRMS (ESI): m/z $[M + H]^+$ calcd for $C_{25}H_{34}NO_2S_2$: 444.2025; found: 444.2023.

2-[(4-Methylphenyl)sulfanyl]-1-[(4-methylphenyl)sulfonyl]-1H-indole 8e

Chromatography: T, R_f = 0.4, a white solid mp: 112.6–113.9 °C, yield: 0.338 g (86%) (Table S1); 0.323 g (82%) (Table S2).

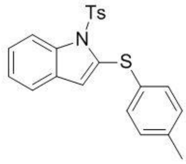

^{1}H NMR (400 MHz, $CDCl_3$) δ 8.24 (d, *J* = 8.4 Hz, 1H), 7.90 (d, *J* = 8.4 Hz, 2H), 7.35–7.27 (m, 4H), 7.24–7.15 (m, 5H), 6.21 (d, *J* = 0.7 Hz, 1H), 2.40 (s, 3H), 2.36 (s, 3H)
^{13}C NMR (101 MHz, $CDCl_3$) δ 144.9, 138.6, 137.9, 135.6, 135.2, 132.6, 130.2, 129.7, 129.7, 129.7, 127.2, 124.3, 123.6, 119.8, 114.7, 113.5, 21.6, 21.2; signals: 18 expected and observed.
IR (ATR) 1444 (m), 1363 (s), 1171 (s), 1084 (m), 1005 (m), 796 (m), 752 (s), 681(s), 586 (s), 538 (s).
HRMS (ESI): m/z $[M + H]^+$ calcd for $C_{22}H_{20}NO_2S_2$: 394.0930; found: 394.0933.

2-(10-Methoxycarbonylodec-1-ylsulfanyl)-1-[(4-methylphenyl)sulfonyl]-1H-indole 8f

Chromatography: PE-$CHCl_3$ (4:1), R_f = 0.2, a waxy solid, yield: 0.376 g (75%) (Table S1); 0.346 g (69%) (Table S2).

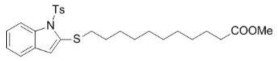

^{1}H NMR (400 MHz, $CDCl_3$) δ 8.24–8.20 (m, 1H), 7.86–7.82 (m, 2H), 7.41–7.38 (m, 1H), 7.30–7.19 (m, 4H), 6.49 (t, *J* = 2.4 Hz, 1H), 3.69 (s, 3H), 2.95 (t, *J* = 7.4 Hz, 2H), 2.35 (s, 3H), 1.71–1.59 (m, 2H), 1.48–1.23 (m, 16H).
^{13}C NMR (101 MHz, $CDCl_3$) δ 174.3, 144.8, 137.8, 135.6, 135, 130, 129.6, 127.1, 124.0, 123.6, 119.5, 114.6, 111.7, 51.5, 35.1, 34.1, 29.4, 29.4, 29.2, 29.1, 28.9, 28.4, 25, 21.6; signals: 24 observed and expected.
IR (ATR) 2920 (w), 2848 (w), 1736 (s), 1441 (m), 1365 (s), 1167 (s), 1090 (s), 744 (s), 584 (s), 540 (s)
HRMS (ESI): m/z $[M + H]^+$ calcd for $C_{27}H_{36}NO_4S_2$: 502.2080; found: 502.2083.

2-(11-Hydroxydodec-1-ylsulfanyl)-1-[(4-methylphenyl)sulfonyl]-1H-indole 8g

Chromatography: $CHCl_3$, R_f = 0.2, a waxy solid, yield: 0.317 g (67%) (Table S1); 0.322 g (68%) (Table S2).

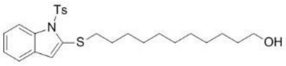

^{1}H NMR (400 MHz, $CDCl_3$) δ 8.24–8.21 (m, 1H), 7.85 (d, *J* = 8.4 Hz, 2H), 7.42–7.38 (m, 1H), 7.24 (ddd, *J* = 8.9, 8.5, 1.3 Hz, 4H), 6.50 (d, *J* = 0.6 Hz, 1H), 3.66 (t, *J* = 6.6 Hz, 2H), 2.96 (t, *J*=7.4 Hz, 2H), 2.36 (s, 3H), 1.48–1.21 (m, 18H).
^{13}C NMR (101 MHz, $CDCl_3$) δ 144.8, 137.8, 135.6, 135, 129.6, 128.2, 127.1, 124.1, 123.6, 119.5, 114.7, 111.8, 63.1, 35.1, 32.8, 29.6, 29.5, 29.5, 29.4, 29.2, 28.9, 28.4, 25.7, 21.6; signals: 24 expected and observed.
IR (ATR) 3593 (w), 2922 (m), 2846 (m), 1442 (s), 1361 (s), 1167 (s), 1090 (s), 744 (s), 685 (s), 584 (s), 538 (s)
HRMS (ESI): m/z $[M + H]^+$ calcd for $C_{26}H_{36}NO_3S_2$: 474.2131; found: 474.2129.

2-[(4-Cyanobenzyl)sulfanyl]-1-[(4-methylphenyl)sulfonyl]-1H-indole 8h

Chromatography: toluene, $R_f = 0.2$, a red solid mp: 148.1–150.0 °C, yield: 0.272 g (65%) (Table S1); 0.289 g (69%) (Table S2).

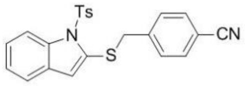

^{1}H NMR (400 MHz, CDCl$_3$) δ 8.29 (dd, *J* = 8.4, 0.8 Hz, 1H), 7.85–7.81 (m, 2H), 7.57–7.54 (m, 2H), 7.40–7.34 (m, 3H), 7.29–7.24 (m, 2H), 7.22 (d, *J* = 8.2 Hz, 2H), 6.46 (d, *J* = 0.6 Hz, 1H), 4.23 (s, 2H), 2.37 (s, 3H).
^{13}C NMR (101 MHz, CDCl$_3$) δ 145.1, 142.4, 138.4, 135.5, 132.3, 130.9, 129.8, 129.7, 129.1, 127.1, 125.3, 123.9, 120.4, 118.7, 117.5, 115.0, 111.1, 40.5, 21.6; signals: 19 expected and observed.
IR (ATR) 2922(w), 2227 (m), 1437 (m), 1371 (s), 1169 (s), 1088 (s), 748 (s), 673 (s), 579 (s), 536 (s)
HRMS (ESI): m/z [M + H]$^+$ calcd for $C_{23}H_{19}N_2O_2S_2$: 419.0882; found: 419.0888.

2-(Benzylsulfanyl)-1-[(4-methylphenyl)sulfonyl]-1H-indole 8i

Chromatography: toluene, $R_f = 0.3$, a waxy yellow solid, yield: 0.342 g (87%) (Table S1); 0.334 g (85%) (Table S2).

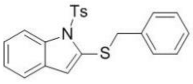

^{1}H NMR (400 MHz, CDCl$_3$) δ 8.26 (d, *J* = 8.4 Hz, 1H), 7.85 (d, *J* = 8.4 Hz, 2H), 7.57 (dd, *J* = 15.7, 5.7 Hz, 1H), 7.39–7.33 (m, 2H), 7.33–7.29 (m, 5H), 7.21 (d, *J* = 8.0 Hz, 2H), 6.47 (d, *J* = 0.6 Hz, 1H), 4.19 (s, 2H), 2.37 (s, 3H).
^{13}C NMR (101 MHz, CDCl$_3$) δ 144.9, 138.0, 136.3, 135.7, 133.2, 129.9, 129.6, 129.1, 128.5, 127.4, 127.2, 124.6, 123.6, 120, 114.8, 114.7, 40.6, 21.6; 18 expected and observed.
IR (ATR) 2924 (w), 1597 (w), 1442 (m), 1367 (s), 1169 (s), 1119 (s), 1088 (s), 748 (s), 677 (s), 575 (s), 536 (s)
HRMS (ESI): m/z [M + H]$^+$ calcd for $C_{22}H_{20}NO_2S_2$: 394.0930; found: 394.0933.

2-(Undec-10-en-1-ylsulfanyl)-5-methoxy-1-[(4-methylphenyl)sulfonyl]-1H-indole 8j

Chromatography: toluene, $R_f = 0.4$, a white solid mp: 45.6–46.3 °C, yield: 0.345 g (71%) (Table S1); 0.379 g (78%) (Table S2).

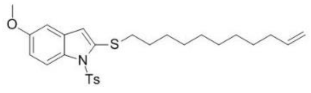

^{1}H NMR (400 MHz, CDCl$_3$) δ 8.11 (d, *J* = 9.1 Hz, 1H), 7.81 (d, *J* = 8.4 Hz, 2H), 7.20 (d, *J* = 8.0 Hz, 2H), 6.85 (d, *J* = 2.5 Hz, 2H), 6.44–6.41 (m, 1H), 5.89–5.78 (m, 1H), 5.05–4.93 (m, 2H), 3.83 (s, 3H), 2.95 (t, *J* = 7.4 Hz, 2H), 2.36 (s, 3H), 1.47–1.26 (m, 16H).
^{13}C NMR (101 MHz, CDCl$_3$) δ 156.7, 145.0, 139.1, 135.7, 132.5, 131.0, 129.8, 129.6, 127.1, 124.4, 115.6, 114.6, 111.9, 102.2, 55.6, 35.1, 33.9, 31.9, 29.5, 29.3, 28.9, 28.5, 28.4, 21.6; signals: 25 expected, 24 observed.
IR (ATR) 2924 (m), 2850 (m), 1604 (w), 1456 (m), 1427 (m), 1375 (m), 1213 (m), 1151 (s), 1034 (m), 812 (m), 671 (s), 602 (s), 540 (s)
HRMS (ESI): m/z [M + H]$^+$ calcd for $C_{27}H_{36}NO_3S_2$: 486.2131; found: 486.2135.

2-(Dec-1-ylsulfanyl)-5-methoxy-1-[(4-methylphenyl)sulfonyl]-1H-indole 8k

Chromatography: toluene, $R_f = 0.4$, a yellow oil, yield: 0.384 g (81%) (Table S1); 0.384 g (81%) (Table S2).

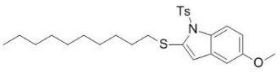

^{1}H NMR (400 MHz, $CDCl_3$) δ 8.11 (d, *J* = 9.1 Hz, 1H), 7.81 (d, *J* = 8.4 Hz, 2H), 7.20 (d, *J* = 8.0 Hz, 2H), 6.93–6.83 (m, 2H), 6.43 (d, *J* = 0.5 Hz, 1H), 3.83 (s, 3H), 2.95 (t, *J* = 7.4 Hz, 2H), 2.36 (s, 3H), 1.73–1.62 (m, 2H), 1.41–1.23 (m, 16H), 0.93–0.89 (m, 3H).

^{13}C NMR (101 MHz, $CDCl_3$) δ 156.6, 144.7, 135.7, 132.5, 131.0, 129.8, 129.6, 127.1, 115.6, 112.6, 111.9, 102.2, 55.8, 35.1, 31.9, 29.5, 29.3, 28.9, 28.5, 28.4, 25.6, 22.7, 21.6, 14.1; signals: 24 expected and observed.

IR (ATR) 2952 (w), 2924 (m), 2848 (w), 1597 (w), 1458 (m), 1435 (m), 1369 (m), 1213 (s), 1178 (s), 1153 (s), 814 (s), 669 (s), 600 (s), 542 (s)

HRMS (ESI): m/z [M + H]$^+$ calcd for $C_{26}H_{36}NO_3S_2$: 474.2131; found: 474.2134.

2-(4-Methylphenylsulfanyl)-5-methoxy-1-[(4-methylphenyl)sulfonyl]-1H-indole 8l

Chromatography: toluene, R_f = 0.4, a yellow oil, yield: 0.364 g (86%) (Table S1); 0.347 g (82%) (Table S2).

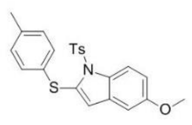

^{1}H NMR (400 MHz, $CDCl_3$) δ 8.11 (d, *J* = 9.1 Hz, 1H), 7.87–7.83 (m, 2H), 7.34–7.30 (m, 2H), 7.19 (dd, *J* = 19.8, 8.0 Hz, 4H), 6.89 (dd, *J* = 9.1, 2.6 Hz, 1H), 6.76 (d, *J* = 2.5 Hz, 1H), 6.12 (d, *J* = 0.6 Hz, 1H), 3.80 (s, 3H), 2.38 (s, 3H). 2.37 (s, 3H).

^{13}C NMR (101 MHz, $CDCl_3$) δ 157.6, 144.9, 138.6, 137.9, 135.6, 135.2, 132.6, 130.2, 129.7, 129.7, 127.2, 124.3, 123.6, 119.8, 114.7, 113.5, 55.8, 21.6, 21.2; signals: 19 expected and observed.

IR (ATR) 2921 (w), 1604 (w), 1456 (w), 1429 (m), 1365 (s), 1211 (s), 1176 (s), 1149 (s), 1110 (m), 1083 (m), 1033 (s), 811 (s), 670 (s), 597 (s), 538 (s)

HRMS (ESI): m/z [M + H]$^+$ calcd for $C_{23}H_{22}NO_3S_2$: 424.1036; found: 424.1040

2-(10-Methoxycarbonyldec-1-ylsulfanyl)-5-methoxy-1-[(4-methylphenyl)sulfonyl]-1H-indole 8m

Chromatography: T-PE (1:4), R_f = 0.4, a waxy solid, yield: 0.383 g (72%) (Table S1); 0.356 g (67%) (Table S2).

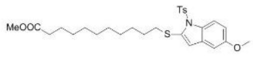

^{1}H NMR (400 MHz, $CDCl_3$) δ 8.11 (d, *J* = 9.1 Hz, 1H), 7.81 (d, *J* = 8.4 Hz, 2H), 7.20 (d, *J* = 7.9 Hz, 2H), 6.91–6.84 (m, 2H), 6.43 (s, 1H), 3.83 (s, 3H), 3.69 (s, 3H), 2.95 (t, *J* = 7.4 Hz, 2H), 2.35 (s, 3H), 1.72–1.62 (m, 4H), 1.47–1.26 (m, 14H).

^{13}C NMR (101 MHz, $CDCl_3$) δ 174.3, 156.6, 144.7, 135.5, 132.5, 131.0, 129.6, 129.4, 127.1, 115.7, 112.6, 112.4, 102.2, 55.6, 51.5, 35.1, 34.1, 29.4, 29.4, 29.2, 29.1, 28.9, 28.4, 25.0, 21.6; signals: 26 expected, 25 observed.

IR (ATR) 2925 (m), 2852 (w), 1734 (m), 1456 (m), 1433 (m), 1369 (m), 1173 (s), 1149 (s), 1090 (m), 1032 (m), 671 (s), 592 (s), 538 (s)

HRMS (ESI): m/z [M + H]$^+$ calcd for $C_{28}H_{38}NO_5S_2$: 532.2186; found: 532.2187.

2-(11-Hydroxyundec-1-ylsulfanyl)-5-methoxy-1-[(4-methylphenyl)sulfonyl]-1H-indole 8n

Chromatography: $CHCl_3$, R_f = 0.3, a yellow oil, yield: 0.393 g (78%) (Table S1); 0.383 g (76%) (Table S2).

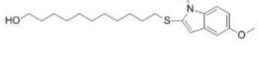

^{1}H NMR (400 MHz, $CDCl_3$) δ 8.10 (d, *J* = 9.1 Hz, 1H), 7.80 (d, *J* = 8.4 Hz, 2H), 7.20 (d, *J* = 8.0 Hz, 2H), 6.91–6.83 (m, 2H), 6.43 (d, *J* = 0.5 Hz, 1H), 3.83 (s, 3H), 3.66 (t, *J* = 6.6 Hz, 2H), 2.95 (t, *J* = 7.4 Hz, 2H), 2.35 (s, 3H), 1.47–1.23 (m, 18H).

^{13}C NMR (101 MHz, CDCl$_3$) δ 156.6, 144.7, 135.6, 135.5, 132.5, 131.0, 129.6, 127.1, 115.7, 112.6, 112.0, 102.2, 63.1, 55.6, 35.1, 32.8, 29.6, 29.5, 29.5, 29.4, 29.1, 28.8, 28.4, 25.7, 21.6; signals: 25 expected and observed.
IR (ATR) 3583 (w), 2924 (s), 2850 (s), 1599 (m), 1456 (s), 1358 (m), 1176 (s), 1151 (s), 1086 (s), 1034 (s), 816 (s), 673 (s), 602 (s), 540 (s)
HRMS (ESI): m/z [M + H]$^+$ calcd for $C_{27}H_{38}NO_4S_2$: 504.2237; found: 504.2241.

2-[(4-Cyanobenzyl)sulfanyl]-5-methoxy-1-[(4-methylphenyl)sulfonyl]-1H-indole 8o

Chromatography: toluene, R$_f$ = 0.2, a red solid mp: 116.1–117.7 °C, yield: 0.323 g (72%) (Table S1); 0.305 g (68%) (Table S2).

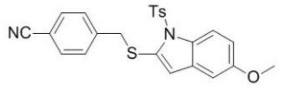

^{1}H NMR (400 MHz, CDCl$_3$) δ 8.17 (d, *J* = 9.2 Hz, 1H), 7.79 (d, *J* = 8.4 Hz, 2H), 7.60–7.50 (m, 2H), 7.31–7.25 (m, 4H), 6.98–6.97 (m, 1H), 6.80 (d, *J* = 2.6 Hz, 1H), 6.37 (s, 1H), 4.22 (s, 2H), 3.83 (s, 3H), 2.37 (s, 3H).
^{13}C NMR (101 MHz, CDCl$_3$) δ 156.7, 144.7, 142.4, 138.4, 135.5, 132.3, 130.9, 129.7, 129.1, 127.1, 125.3, 123.9, 120.4, 118.7, 117.5, 115.0, 111.1, 55.8, 40.5, 21.6; signals: 20 expected and observed.
IR (ATR) 3302 (w), 2924 (w), 2225 (w), 1658 (w), 1435 (m), 1367 (m), 1161 (s), 1028 (s), 671 (s), 567 (s), 542 (s)
HRMS (ESI): m/z [M + H]$^+$ calcd for $C_{24}H_{21}N_2O_3S_2$: 449.0988; found: 449.0992.

2-(Benzylsulfanyl)-5-methoxy-1-[(4-methylphenyl)sulfonyl]-1H-indole 8p

Chromatography: T-PE (1:4), R$_f$ = 0.3, a yellowish solid; mp: 116.1–117.7 °C, yield: 0.347 g (82%) (Table S1); 0.347 g (82%) (Table S2).

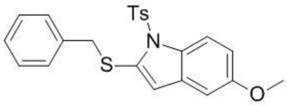

^{1}H NMR (400 MHz, CDCl$_3$) δ 8.14 (d, *J* = 9.1 Hz, 1H), 7.81 (d, *J* = 8.4 Hz, 2H), 7.32–7.28 (m, 5H), 7.21–7.19 (m, 2H), 6.93 (dd, *J* = 9.1, 2.6 Hz, 1H), 6.81 (d, *J* = 2.5 Hz, 1H), 6.39 (d, *J* = 0.6 Hz, 1H), 4.19 (s, 2H), 3.82 (s, 3H), 2.36 (s, 3H).
^{13}C NMR (101 MHz, CDCl$_3$) δ 156.7, 144.7, 142.4, 138.4, 135.5, 132.3, 130.9, 129.7, 129.1, 127.1, 125.3, 123.9, 120.4, 118.7, 117.5, 115.0, 55.8, 40.5, 21.6; signals: 19 expected and observed.
IR (ATR) 2922 (w), 2224 (w), 1593 (w), 1504 (w), 1437 (m), 1371 (s), 1169 (s), 795 (s), 665 (s), 592 (s), 540 (s)
HRMS (ESI): m/z [M + H]$^+$ calcd for $C_{23}H_{22}NO_3S_2$: 424.1036; found: 424.1035.

2-(Undec-10-en-1-ylsulfanyl)-5-bromo-1-[(4-methylphenyl)sulfonyl]-1H-indole 8r

Chromatography: T-PE (1:4), R$_f$ = 0.3, a waxy solid, yield: 0.369 g (69%) (Table S1); 0.396 g (74%) (Table S2).

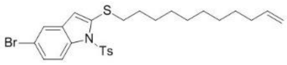

^{1}H NMR (400 MHz, CDCl$_3$) δ 8.08 (d, *J* = 8.9 Hz, 1H), 7.84–7.81 (m, 2H), 7.51 (d, *J* = 1.7 Hz, 1H), 7.38-7.32 (m, 1H), 7.25–7.22 (m, 2H), 6.38 (d, *J* = 0.6 Hz, 1H), 5.84 (ddt, *J* = 16.9, 10.2, 6.7 Hz, 1H), 5.05–4.94 (m, 2H), 2.95 (t, *J* = 7.4 Hz, 2H), 2.38 (s, 3H), 2.10–1.95 (m, 2H), 1.75–1.60 (m, 2H), 1.49–1.27 (m, 12H).
^{13}C NMR (101 MHz, CDCl$_3$) δ 145.2, 139.2, 137.1, 136.4, 135.2, 131.7, 129.7, 127.1, 126.6, 122, 117.1, 115.9, 114.2, 109.5, 35.1, 34.8, 34.8, 29.4, 29.1, 29.1, 28.9, 28.9, 28.3, 21.6; signals: 24 observed and expected.
IR (ATR) 2925 (m), 2852 (m), 1595 (w), 1435 (m), 1373(s), 1223 (m), 1173 (s), 1088 (m), 910 (m), 804 (m), 706 (s), 665 (s), 592 (s), 540 (s)

HRMS (ESI): m/z [M + H]+ calcd for $C_{26}H_{33}BrNO_2S_2$: 534.1131; found: 534.1136.

2-(Dec-1-ylsulfanyl)-5-bromo-1-[(4-methylphenyl)sulfonyl]-1H-indole 8s

Chromatography: T-PE (1:4), R_f = 0.3, a yellow oil, yield: 0.439 g (84%) (Table S1); 0.434 g (83%) (Table S2).

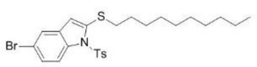

^{1}H NMR (400 MHz, CDCl$_3$) δ 8.08 (d, *J* = 8.9 Hz, 1H), 7.85–7.80 (m, 2H), 7.51 (d, *J* = 1.9 Hz, 1H), 7.38–7.32 (m, 1H), 7.25–7.21 (m, 2H), 6.38 (d, *J* = 0.5 Hz, 1H), 2.95 (t, *J* = 7.4 Hz, 2H), 2.38 (s, 3H), 1.72–1.60 (m, 2H), 1.49–1.24 (m, 14H), 0.91 (t, *J* = 7.1 Hz, 3H).
^{13}C NMR (101 MHz, CDCl$_3$) δ 145.2, 137.2, 136.4, 135.2, 131.7, 129.7, 127.2, 126.6, 122.0, 117.1, 115.9, 109.5, 34.8, 31.9, 29.6, 29.5, 29.3, 29.2, 28.9, 28.3, 22.7, 21.3, 14.1; signals: 23 expected and observed.
IR (ATR) 2943 (w), 2922 (m), 2852 (m), 1595 (w), 1437 (m), 1375 (s), 1171 (s), 1090 (s), 800 (m), 706 (s), 663 (s), 590 (s), 540 (s)
HRMS (ESI): m/z [M + H]+ calcd for $C_{25}H_{33}BrNO_2S_2$: 522.1131; found: 522.1132.

2-(4-Methylphenylsulfanyl)-5-bromo-1-[(4-methylphenyl)sulfonyl]-1H-indole 8t

Chromatography: PE-DCM (4:1), R_f = 0.4, a waxy solid, yield: 0.430 g (91%) (Table S1); 0.406 g (86%) (Table S2).

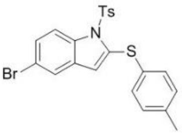

^{1}H NMR (400 MHz, CDCl$_3$) δ 8.07 (d, *J* = 8.9 Hz, 1H), 7.88 (d, *J* = 8.4 Hz, 2H), 7.40 (d, *J* = 1.9 Hz, 1H), 7.36 (d, *J* = 2.0 Hz, 1H), 7.35–7.32 (m, 2H), 7.25 (d, *J* = 8.0 Hz, 2H), 7.19 (d, *J* = 7.9 Hz, 2H), 5.99 (d, *J* = 0.6 Hz, 1H), 2.40 (s, 3H), 2.39 (s, 3H).
^{13}C NMR (101 MHz, CDCl$_3$) δ 145.3, 139.3, 137.9, 136.4, 135.2, 133.3, 131.5, 130.1, 129.8, 128.6, 127.2, 126.7, 122.2, 117.1, 115.9, 111.0, 21.7, 21.3; signals: 18 expected and observed.
IR (ATR) 1595 (w), 1439 (m), 1367 (m), 1169 (s), 1173 (m), 1086 (m), 804 (m), 665 (s), 598 (s), 540 (s)
HRMS (ESI): m/z [M + H]+ calcd for $C_{22}H_{19}BrNO_2S_2$: 472.0035; found: 472.0037.

2-(10-Methoxycarbonylodec-1-ylsulfanyl)-5-bromo-1-[(4-methylphenyl)sulfonyl]-1H-indole 8u

Chromatography: T, R_f = 0.4, a waxy solid, yield: 0.459 g (79%) (Table S1); 0.453 g (78%) (Table S2).

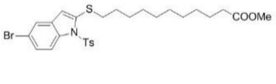

^{1}H NMR (400 MHz, CDCl$_3$) δ 8.08 (d, *J* = 8.9 Hz, 1H), 7.82 (dd, *J* = 8.4, 1.7 Hz, 2H), 7.51 (d, *J* = 2.0 Hz, 1H), 7.38–7.32 (m, 1H), 7.23 (d, *J* = 8.1 Hz, 2H), 6.38 (s, 1H), 3.69 (s, 3H), 2.95 (t, *J* = 7.4 Hz, 2H), 2.38 (s, 3H), 2.33 (t, *J* = 7.5 Hz, 2H), 1.68–1.50 (m, 4H), 1.48–1.26 (m, 12H).
^{13}C NMR (101 MHz, CDCl$_3$) δ 174.3, 145.2, 137.1, 136.4, 135.2, 131.7, 129.7, 127.1, 126.6, 122.0, 117.1, 115.9, 109.6, 51.5, 34.8, 34.1, 29.4, 29.4, 29.2, 29.1, 28.9, 28.3, 24.9, 21.6; signals: 25 expected, 24 observed.
IR (ATR) 2925 (m), 2852 (m), 1734 (s), 1437 (s), 1367 (s), 1169 9s), 708 (s), 663 (s), 582 (s), 540 (s)
HRMS (ESI): m/z [M + H]+ calcd for $C_{27}H_{35}BrNO_4S_2$: 580.1185; found: 580.1181.

2-(11-Hydroxyundec-1-ylsulfanyl)-5-bromo-1-[(4-methylphenyl)sulfonyl]-1H-indole 8w

Chromatography: $CHCl_3$, R_f = 0.3, a waxy solid, yield: 0.420 g (76%) (Table S1); 0.420 g (76%) (Table S2).

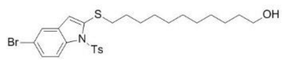

^{1}H NMR (400 MHz, $CDCl_3$) δ 8.08 (d, *J* = 8.9 Hz, 1H), 7.82 (d, *J* = 8.4 Hz, 2H), 7.51 (d, *J* = 1.7 Hz, 1H), 7.38–7.32 (m, 1H), 7.23 (d, *J* = 8.0 Hz, 2H), 6.38 (d, *J* = 0.6 Hz, 1H), 3.67 (t, *J* = 6.6 Hz, 2H), 2.95 (t, *J* = 7.4 Hz, 2H), 2.38 (s, 3H), 1.75–1.60 (m, 2H), 1.48–1.21 (m, 16H).
^{13}C NMR (101 MHz, $CDCl_3$) δ 145.2, 137.1, 136.4, 135.2, 131.7, 129.7, 127.1, 126.6, 122.0, 117.1, 115.9, 109.6, 63.1, 34.8, 32.8, 29.6, 29.5, 29.5, 29.4, 29.1, 28.9, 28.3, 25.7, 21.6; signals: 24 expected and observed.
IR (ATR) 3359 (w), 2916 (s), 2850 (s), 1441 (s), 1379 (s), 1169 (s), 1092 (s), 806 (m), 706 (s), 665 (s), 579 (s), 536 (s)
HRMS (ESI): m/z [M + H]$^+$ calcd for $C_{26}H_{35}BrNO_3S_2$: 552.1236; found: 552.1238.

2-[(4-Cyanobenzyl)sulfanyl]-5-bromo-1-[(4-methylphenyl)sulfonyl]-1H-indole 8x

Chromatography: PE-$CHCl_3$ (4:1), R_f = 0.5, a waxy solid, yield: 0.343 g (69%) (Table S1); 0.338 g (68%) (Table S2).

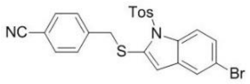

^{1}H NMR (400 MHz, $CDCl_3$) δ 8.15 (d, *J* = 9.0 Hz, 1H), 7.80 (d, *J* = 8.4 Hz, 2H), 7.57 (d, *J* = 8.4 Hz, 2H), 7.51 (d, *J* = 1.7 Hz, 1H), 7.45–7.41 (m, 1H), 7.37 (d, *J* = 8.4 Hz, 2H), 7.24 (d, *J* = 8.0 Hz, 2H), 6.36 (d, *J* = 0.6 Hz, 1H), 4.23 (s, 2H), 2.39 (s, 3H).
^{13}C NMR (101 MHz, $CDCl_3$) δ 145.1, 142.4, 138.4, 135.5, 132.3, 130.9, 129.8, 129.7, 129.1, 127.1, 125.3, 123.9, 120.4, 118.7, 117.5, 115.0, 111.1, 40.5, 21.6; signals: 19 expected and observed.
IR (ATR) 2922 (w), 2224 (w), 1593 (w), 1504 (w), 1437 (m), 1371 (s), 1169 (s), 795 (s), 665 (s), 592 (s), 540 (s) cm^{-1}
HRMS (ESI): m/z [M + H]$^+$ calcd for $C_{23}H_{18}BrN_2O_2S_2$: 496.9988; found: 496.9991.

2-(Benzylsulfanyl)-5-bromo-1-[(4-methylphenyl)sulfonyl]-1H-indole 8y

Chromatography: PE-$CHCl_3$ (2:1), R_f = 0.3, a waxy solid, yield: 0.402 g (85%) (Table S1); 0.397 g (84%) (Table S2).

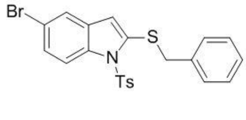

^{1}H NMR (400 MHz, CDCl3) δ 8.12 (d, *J* = 8.9 Hz, 1H), 7.82 (d, *J* = 8.4 Hz, 2H), 7.49 (d, *J* = 1.8 Hz, 1H), 7.38–7.34 (m, 1H), 7.33–7.29 (m, 5H), 7.23 (d, *J* = 8.1 Hz, 2H), 6.36 (d, *J* = 0.6 Hz, 1H), 4.18 (s, 2H), 2.38 (s, 3H).
^{13}C NMR (101 MHz, $CDCl_3$) δ 144.9, 138.0, 136.3, 135.7, 133.2, 129.9, 129.6, 129.1, 128.5, 127.4, 124.6, 123.6, 120.0, 118.2, 117.6, 114.7, 40.6, 21.6; 18 expected and observed.
IR (ATR) 2922 (w), 1593 (w), 1439 (m), 1371 (s), 1167 (s), 1084 (m), 802 (s), 696 (s), 667 (s), 596 (s), 579 (s), 536 (s) cm^{-1}
HRMS (ESI): m/z [M + H]$^+$ calcd for $C_{22}H_{19}BrNO_2S_2$: 472.0035; found: 472.0043.

2-(Undec-10-en-1-ylsulfanyl)-5-[[(1,1-dimethylethoxy)carbonyl]amino]-1-[(4-methylphenyl)sulfonyl]-1H-indole 8z

Chromatography: PE-$CHCl_3$ (2:1), R_f = 0.5, a waxy solid, yield: 0.371 g (65%) (Table S1); 0.400 g (70%) (Table S2).

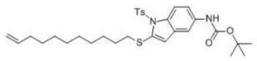

^{1}H NMR (400 MHz, $CDCl_3$) δ 8.10 (d, *J* = 8.9 Hz, 1H), 7.79 (d, *J* = 8.4 Hz, 2H), 7.59 (s, 1H), 7.19 (d, *J* = 8.2 Hz, 2H), 7.15–7.07 (m, 1H), 6.54 (brs, 1H), 6.41 (s, 1H), 5.86–5.81 (m, 1H), 4.97–4.91 (m, 2H) 2.93 (t, *J* = 7.4 Hz, 2H), 2.35 (s, 3H), 2.10–1.95 (m, 2H), 1.60–1.52 (m, 2H), 1.54 (s, 9H), 1.48–1.20 (m, 12H).

^{13}C NMR (101 MHz, $CDCl_3$) δ 156.5, 145.1, 141.0, 140.1, 135.2, 133.0, 131.0, 129.9, 128.1, 127.2, 124.1, 119.6, 115.4, 113.6, 109.1, 81.9, 38.4, 32.8, 29.4, 28.7, 28.7, 28.1, 27.1, 25.7, 21.6; signals: 27 expected, 25 observed.

IR (ATR) 2915 (m), 2850 (m), 1440 (s), 1378 (s), 1166 (s), 700 (s), 665 (s), 578 (s), 536 (s) cm^{-1}

HRMS (ESI): m/z $[M + H]^+$ calcd for $C_{31}H_{43}N_2O_4S_2$: 571.2659; found: 571.2664.

2-(Dec-1-ylsulfanyl)-5-[[(1,1-dimethylethoxy)carbonyl]amino]-1-[(4-methylphenyl)sulfonyl]-1H-indole 8aa

Chromatography: PE-$CHCl_3$ (2:1), R_f = 0.3, a waxy solid, yield: 0.380 g (68%) (Table S1); 0.441 g (79%) (Table S2).

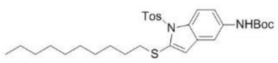

^{1}H NMR (400 MHz, $CDCl_3$) δ 8.10 (d, *J* = 8.9 Hz, 1H), 7.79 (d, *J* = 8.4 Hz, 2H), 7.59 (d, *J* = 3.7 Hz, 1H), 7.21–7.19 (m, 2H), 7.15–7.07 (m, 1H), 6.51 (s, 1H), 6.42 (s, 1H), 2.93 (t, *J* = 7.4 Hz, 2H), 2.36 (s, 3H), 1.71–1.61 (m, 2H), 1.54 (s, 9H), 1.48–1.19 (m, 14H), 0.91 (t, *J* = 6.8 Hz, 3H).

^{13}C NMR (101 MHz, $CDCl_3$) δ 156.5, 145.1, 141.0, 135.2, 133.0, 131.0, 129.9, 129.3, 126.9, 124.1, 119.6, 113.6, 109.1, 81.9, 38.4, 34.8, 31.9, 29.5, 29.3, 28.9, 28.3, 22.7, 21.3, 14.1; signals: 26 expected, 24 observed

IR (ATR) 3365 (w), 2924 (m), 2852 (m), 1720 (s), 1537 (m), 1454 (s), 1365 (s), 1160 (s), 806 (s), 675 (s), 584 (s), 542 (s)

HRMS (ESI): m/z $[M + H]^+$ calcd for $C_{30}H_{43}N_2O_4S_2$: 559.2659; found: 559.2663.

2-(4-Methylphenylsulfanyl)-5-[[(1,1-dimethylethoxy)carbonyl]amino]-1-[(4-methylphenyl)sulfonyl]-1H-indole 8ab

Chromatography: Toluene, R_f = 0.3, a waxy solid, yield: 0.366 g (72%) (Table S1); 0.361 g (71%) (Table S2).

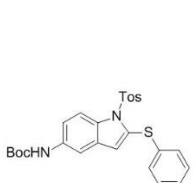

^{1}H NMR (400 MHz, $CDCl_3$) δ 7.89 (d, *J* = 8.8 Hz, 1H), 7.76 (d, *J* = 8.4 Hz, 2H), 7.57 (d, *J* = 3.7 Hz, 1H), 7.35-6.95 (m, 7H), 6.47 (brs, 1H), 6.12 (d, J= 0.6 Hz, 1H), 2.38 (s, 3H), 2.37 (s, 3H), 1.53 (s, 9H).

^{13}C NMR (101 MHz, $CDCl_3$) δ 156.1, 145.1, 141.2, 137.9, 135.2, 134.9, 133.1, 130.9, 129.9, 127.6, 127.1, 126.8, 123.7, 115.2, 113.5, 109.1, 82.5, 28.1, 21.6, 21.2; signals: 21 expected, 20 observed.

IR (ATR) 2924 (w), 2364 (w), 1705 (w), 1452 (m), 1365 (m), 1155 (s), 808 (s), 671 (s), 582 (s), 542 (s)

HRMS (ESI): m/z $[M + H]^+$ calcd for $C_{27}H_{29}N_2O_4S_2$: 509.1563; found: 509.1569.

2-(10-Methoxycarbonylodec-1-ylsulfanyl)-5-[[(1,1-dimethylethoxy)carbonyl]amino]-1-[(4-methylphenyl)sulfonyl]-1H-indole 8ac

Chromatography: toluene, R_f = 0.1, a yellowish oil, yield: 0.401 g (65%) (Table S1); 0.382 g (62%) (Table S2).

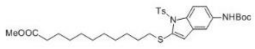

^{1}H NMR (400 MHz, $CDCl_3$) δ 7.93 (d, *J* = 8.8 Hz, 1H), 7.76 (d, *J* = 8.4 Hz, 2H), 7.57 (d, *J* = 3.7 Hz, 1H), 7.38 (s, 1H), 7.22 (d, *J* = 8.1 Hz, 2H), 7.21–7.17 (m, 1H), 6.62 (d, *J* = 4.2 Hz, 1H), 3.67 (s, 3H), 2.81 (t, *J* = 7.3 Hz, 2H), 2.34 (s, 3H), 2.33 (t, *J* = 7.5 Hz, 2H), 1.70–1.50 (m, 4H), 1.48 (s, 9H), 1.47–1.20 (m, 12H).

^{13}C NMR (101 MHz, $CDCl_3$) δ 174.3, 156.5, 145.1, 141.0, 135.2, 133.0, 131.0, 129.9, 128.1, 126.9, 124.1, 119.6, 113.6, 109.1, 81.9, 52.3, 38.4, 31.9, 29.5, 29.3, 28.9, 28.9, 28.3, 27.9, 22.7, 21.3; signals: 28 expected, 26 observed

IR (ATR) 2924 (m), 2364 (m), 1705 (s), 1734 (s), 1437 (s), 1367 (s), 1155 (s), 808 (s), 671 (s), 582 (s), 542 (s)

HRMS (ESI): m/z $[M + H]^+$ calcd for $C_{32}H_{45}N_2O_6S_2$: 617.2714; found: 617.2711.

2-(11-Hydroxyundec-1-ylsulfanyl)-5-[[(1,1-dimethylethoxy)carbonyl]amino]-1-[(4-methylphenyl)sulfonyl]-1H-indole 8ad

Chromatography: chloroform, R_f = 0.1, a yellowish oil, yield: 0.418 g (71%) (Table S1); 0.406 g (69%) (Table S2).

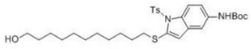

^{1}H NMR (400 MHz, $CDCl_3$) δ 7.94 (d, *J* = 8.8 Hz, 1H), 7.77 (d, *J* = 8.4 Hz, 2H), 7.58 (d, *J* = 3.7 Hz, 1H), 7.39 (s, 1H), 7.24 (d, *J* = 8.0 Hz, 2H), 7.18-7.15 (m, 1H), 6.64 (d, *J* = 4.2 Hz, 1H), 3.67 (t, *J* = 6.6 Hz, 2H), 2.83 (t, *J* = 7.4 Hz, 2H), 2.37 (s, 3H), 1.70-1.50 (m, 4H), 1.47 (s, 9H), 1.38–1.25 (m, 14H).

^{13}C NMR (101 MHz, $CDCl_3$) δ 156.5, 145.1, 141.0, 135.2, 133.0, 131.0, 129.9, 128.1, 127.2, 124.1, 119.6, 113.6, 109.1, 81.9, 63.1, 38.4, 32.8, 29.6, 29.4, 29.2, 29.2, 28.7, 28.1, 27.1, 25.7, 21.6; signals: 27 expected, 26 observed.

IR (ATR) 2924 (m), 2364 (m), 1705 (s), 1734 (s), 1437 (s), 1169 (s), 1092 (s), 806 (m), 706 (s), 665 (s), 579 (s), 536 (s)

HRMS (ESI): m/z $[M + H]^+$ calcd for $C_{31}H_{45}N_2O_5S_2$: 589.2764; found: 589.2762.

2-[(4-Cyanobenzyl)sulfanyl]-5-[[(1,1-dimethylethoxy)carbonyl]amino]-1-[(4-methylphenyl)sulfonyl]-1H-indole 8ae

Chromatography: toluene, R_f = 0.2, a yellowish oil, yield: 0.352 g (66%) (Table S1); 0.336 g (63%) (Table S2).

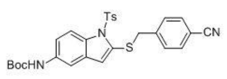

^{1}H NMR (400 MHz, $CDCl_3$) δ 8.15 (d, *J* = 9.0 Hz, 1H), 7.80 (d, *J* = 8.4 Hz, 2H), 7.57 (d, *J* = 8.4 Hz, 2H), 7.51 (d, *J* = 1.7 Hz, 1H), 7.44–7.41 (m, 1H), 7.37 (d, *J* = 8.4 Hz, 2H), 7.24 (d, *J* = 8.0 Hz, 2H), 6.36 (d, *J* = 0.6 Hz, 1H), 4.23 (s, 2H), 2.39 (s, 3H), 1.47 (s, 9H).

^{13}C NMR (101 MHz, $CDCl_3$) δ 156.1, 145.1, 142.1, 137.9, 135.2, 134.9, 133.1, 130.9, 129.9, 129.8, 127.6, 126.8, 123.7, 120.0, 117.5, 116.4, 113.5, 109.1, 82.5, 40.0, 28.1, 21.2; signals: 22 expected and observed.

IR (ATR) 3325 (w), 2922 (w), 2850 (w), 2225 (w), 1689 (m), 1595 (m), 1529 (m), 1456 (s), 1369 (m), 1160 (s), 904 (m), 850 (m), 731 (s), 671 (s), 582 (s), 544 (s) cm^{-1}

HRMS (ESI): m/z $[M + H]^+$ calcd for $C_{28}H_{28}N_3O_4S_2$: 534.1516; found: 534.1521.

2-(Benzylsulfanyl)-5-[[(1,1-dimethylethoxy)carbonyl]amino]-1-[(4-methylphenyl)sulfonyl]-1H-indole 8af

Chromatography: PE-DCM (4:1), R_f = 0.2, a yellowish oil, yield: 0.397 g (78%) (Table S1); 0.396 g (78%) (Table S2).

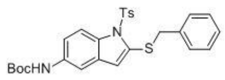

^{1}H NMR (400 MHz, $CDCl_3$) δ 8.14 (d, *J* = 9.0 Hz, 1H), 7.79 (d, *J* = 8.4 Hz, 2H), 7.35–7.25 (m, 5H), 7.21–7.18 (m, 2H), 7.09–6.95 (m, 1H), 6.51 (s, 1H), 6.38 (s, 1H), 4.18 (s, 2H), 2.37 (s, 3H), 1.54 (s, 9H).
^{13}C NMR (101 MHz, $CDCl_3$) δ 156.1, 144.9, 136.3, 135.4, 134.4, 134.2, 130.4, 129.6, 129.1, 128.6, 128.5, 128.0, 127.5, 127.1, 127.1, 115.2, 114.7, 82.5, 40.4, 28.4, 21.6; signals: 21 expected and observed.
IR (ATR) 3325 (w), 2921 (w), 1691 (w), 1452 (m), 1367 (m), 1155 (s), 1087 (m), 810 (w), 700 (m), 671 (s), 582 (s), 541 (s) cm^{-1}
HRMS (ESI): m/z [M + H]$^+$ calcd for $C_{27}H_{29}N_2O_4S_2$: 509.1563; found: 509.1566.

7. General Procedure for the Preparation of 2-(dodecyl-1-sulfenyl)-1-[(4-methylphenyl)sulfonyl]-1*H*-indole 8a and Representative Analytical Data

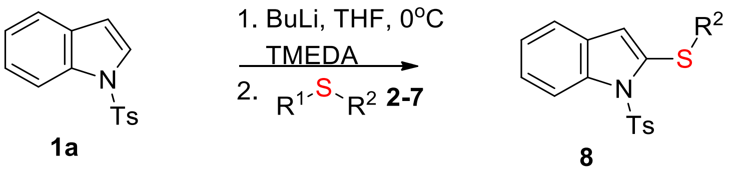

To a stirred, cooled to 0 °C solution of indole **1a** (1 mmol) and *N*,*N*,*N′*,*N′*-tetramethylethane-1,2-diamine (1 mmol) in dry THF (5 mL) was added dropwise n-BuLi (2.5 M in hexane; 1 mmol). Mixture was stirred in 0 °C for 5 min. After that, the solution of thioalkylating agents **2**–**7** (1 mmol) in dry THF (5 mL) was added. Reaction was warmed to room temperature and stirred for 15 min. Mixture was diluted in Et_2O and washed with water, dried over $MgSO_4$, filtered and concentrated under vacuum. The products were purified by column chromatography.

Lp.	R¹	R²	Yield^b [%]
1	**(2a)** 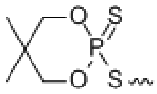	$CH_3(CH_2)_{11}$–	**8a (72)**
2	**(3a)** 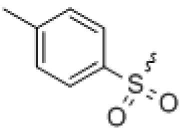	$CH_3(CH_2)_{11}$–	**8a (83)**
3	**(2b)** $CH_3(CH_2)_{11}S$–	$CH_3(CH_2)_{11}$–	**8a (72)**
4	**(2d)** 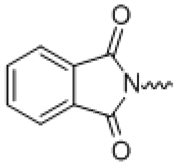	$CH_3(CH_2)_{11}$–	**8a (55)**
5	**(2e)** 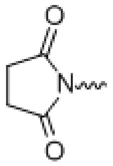	$CH_3(CH_2)_{11}$–	**8a (53)**
6	**(2f)** C_6H_5S	C_6H_5–	**8b (95)**

^{b} isolated yields

2-(Dodec-1-ylsulfanyl)-1-[(4-methylphenyl)sulfonyl]-1H-indole 8a

Chromatography: Petroleum ether (PE)-Toluene (T) (1:2), R_f = 0.5, a yellowish oil, yield: 0.392 g (83%).

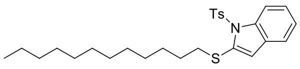

¹H NMR (400 MHz, $CDCl_3$) δ 8.26–8.20 (m, 1H), 7.89–7.80 (m, 2H), 7.46–7.35 (m, 1H), 7.32–7.19 (m, 4H), 6.50 (d, *J* = 0.5 Hz, 1H), 2.96 (t, *J* = 7.4 Hz, 2H), 2.36 (s, 3H), 1.75–1.64 (m, 2H), 1.49–1.40 (m, 2H), 1.37–1.24 (m, 16H), 0.92 (t, *J* = 6.8 Hz, 3H).
¹³C NMR (101 MHz, $CDCl_3$) δ 144.8, 137.8, 135.6, 135.0, 130.0, 129.6, 127.1, 124.0, 123.6, 119.5, 114.7, 111.7, 35.1, 31.9, 29.7, 29.6, 29.6, 29.5, 29.4, 29.2, 28.9, 28.4, 22.7, 21.6, 14.1; signals: 25 expected and 25 observed.
IR (ATR) 2924 (w), 2852 (w), 1441 (m), 1373 (m), 1174 (s), 1088 (m), 677 (s), 580 (s), 542 (s)
HRMS (ESI): m/z $[M + H]^+$ calcd for $C_{27}H_{38}NO_2S_2$: 472.2338; found: 472.2342.

2-[Phenylsulfanyl]-1-[(4-methylphenyl)sulfonyl]-1H-indole 8b

Chromatography: Toluene, R_f = 0.4, a white solid mp: 110–112 °C, yield: 0.349 g (92%).

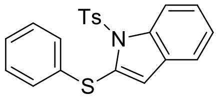

¹H NMR (400 MHz, $CDCl_3$) δ 8.26 (d, *J* = 8.4 Hz, 1H), 7.86 (d, *J* = 8.4 Hz, 2H), 7.57 (dd, *J* = 15.7, 5.7 Hz, 1H), 7.40–7.32 (m, 2H), 7.33–7.29 (m, 5H), 7.21 (d, *J* = 8.0 Hz, 2H), 6.47 (d, *J* = 0.6 Hz, 1H), 2.37 (s, 3H).
¹³C NMR (101 MHz, $CDCl_3$) δ 144.9, 135.6, 131.3, 129.9, 129.6, 127.9, 127.2, 126.8, 124.7, 124.5, 123.7, 120.1, 115.4, 114.8, 113.5, 109.0, 21.6, signals: 17 expected and observed.
IR (ATR) 1444 (m), 1363 (s), 1171 (s), 1084 (m), 1005 (m), 796 (m), 752 (s), 681(s), 586 (s), 538 (s)
HRMS (ESI): m/z $[M + H]^+$ calcd for $C_{21}H_{18}NO_2S_2$: 380.0773; found: 380.0776.

8. General Procedure for Preparation of 2-sulfenyl-*1H*-indoles 9c and 9k and Representative Analytical Data

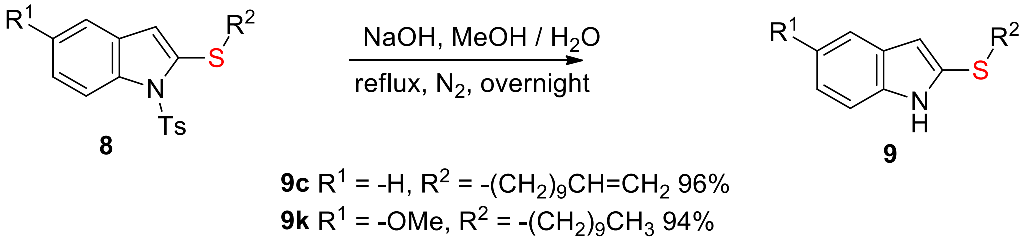

A solution of NaOH (2M, 3 mL) was added to a stirring solution of 2-sulfenyl-1-[(4-methylphenyl)sulfonyl]-1*H*-indole **8c** or **8k** (1.48 mmol) in methanol (10 mL) and the resulting mixture was refluxed under nitrogen overnight. The reaction mixture was concentrated under reduce pressure and extracted with diethyl ether (30 mL), organic layer was washed with brine (10 mL). The combined organic layer was dried over anhydrous $MgSO_4$ and concentrated under reduce pressure. The crude product was purified by silica gel column chromatography.

2-(Undec-10-en-1-ylsulfanyl)-1H-indole 9c

Chromatography: PE-DCM (4:1), R_f = 0.5, a yellowish oil, yield: 0.428 g (96%).

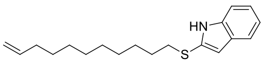

^{1}H NMR (400 MHz, $CDCl_3$) δ 8.28–8.20 (m, 1H), 7.98 (s, 1H), 7.45–7.35 (m, 1H), 7.30–7.10 (m, 2H), 6.50 (d, *J* = 0.7 Hz, 1H), 5.60-5.87 (m, 1H), 5.05–4.94 (m, 2H), 2.96 (t, *J* = 7.4 Hz, 2H), 2.11–2.03 (m, 2H), 1.72–1.60 (m, 2H), 1.49–1.25 (m, 12H).

^{13}C NMR (101 MHz, $CDCl_3$) δ 139.2, 137.8, 135.0, 129.6, 124.0, 123.6, 119.5, 114.6, 114.2, 111.6, 35.1, 33.8, 31.9, 29.6, 29.5, 29.3, 29.2, 28.9, 28.4, signals: 19 expected and observed.

IR (ATR) 2921 (w), 2848 (w), 1441 (m), 1371 (m), 1165 (m), 1088 (m), 746 (s), 683 (s), 584 (s), 538 (s)

HRMS (ESI): m/z [M + H]$^+$ calcd for $C_{19}H_{28}NS$: 302.1937; found: 302.1940.

2-(Dec-1-ylsulfanyl)-5-methoxy-1H-indole 9k

Chromatography: PE-DCM (4:1), R_f = 0.5, a yellowish oil, yield: 0.444g (94%).

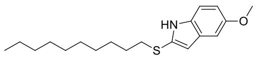

^{1}H NMR (400 MHz, $CDCl_3$) δ 7.97 (brs, 1H), 7.23 (d, *J* = 8.8 Hz, 1H), 7.03 (d, *J* = 2.4 Hz, 1H), 6.87–6.85 (m, 1H), 6.57-6.55 (m, 1H), 3.86 (s, 3H), 2.83 (t, *J* = 7.5 Hz, 2H), 1.67–1.56 (m, 2H), 1.44–1.23 (m, 14H), 0.90 (t, *J* = 6.9 Hz, 3H)

^{13}C NMR (101 MHz, $CDCl_3$) δ 156.3, 132.1, 129.7, 127.1, 112.8, 111.2, 108.3, 101.6, 55.8, 36.8, 31.9, 29.9, 29.5, 29.5, 29.3, 29.2, 28.6, 22.7, 14.1; signals: 19 expected and observed.

IR (ATR) 3396 (w), 2921 (s), 2850 (s), 1620 (s), 1430 (s), 1214 (s), 1160 (s), 1035 (s), 752 (s) cm^{-1}

HRMS (ESI): m/z [M + H]$^+$ calcd for $C_{19}H_{30}NOS$: 320.2043; found: 320.2041.

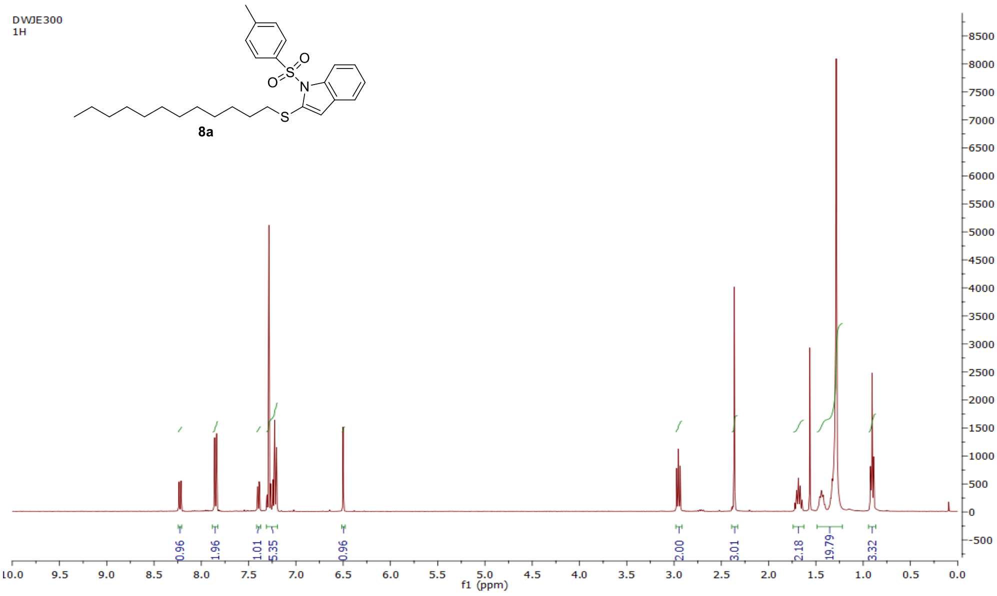

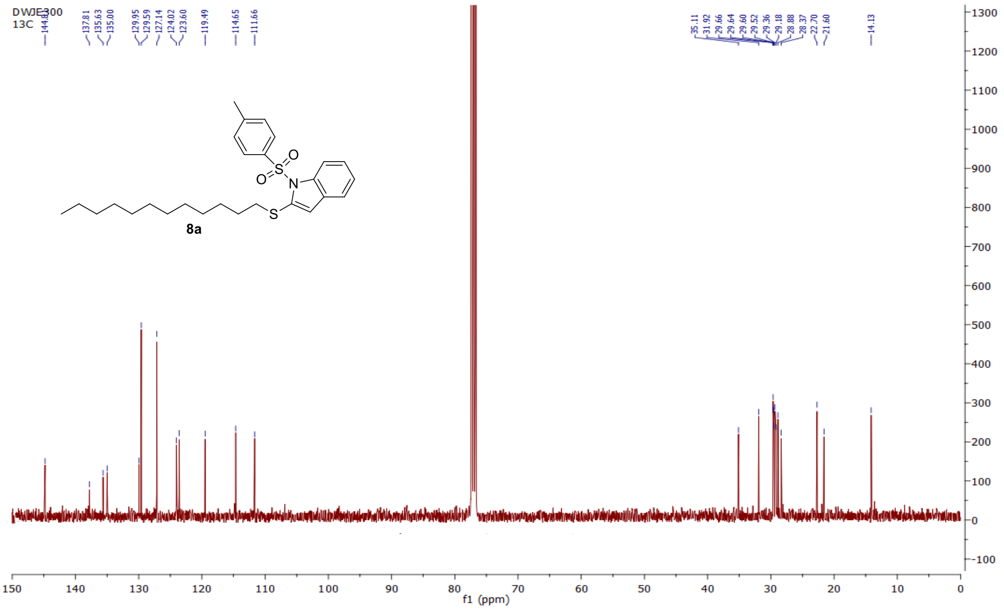
DWJE300
13C
144.81
137.81
135.63
135.00
129.95
129.59
127.14
124.02
123.60
119.49
114.65
111.66
35.11
31.92
29.66
29.64
29.60
29.52
29.36
29.18
28.88
28.37
22.70
21.60
14.13
8a
f1 (ppm)

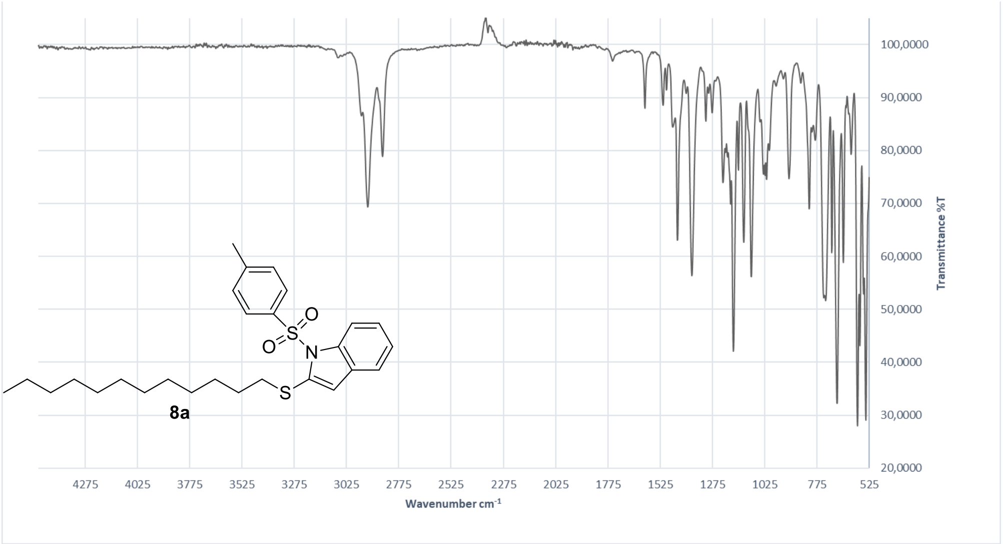
8a
Wavenumber cm-1
Transmittance %T

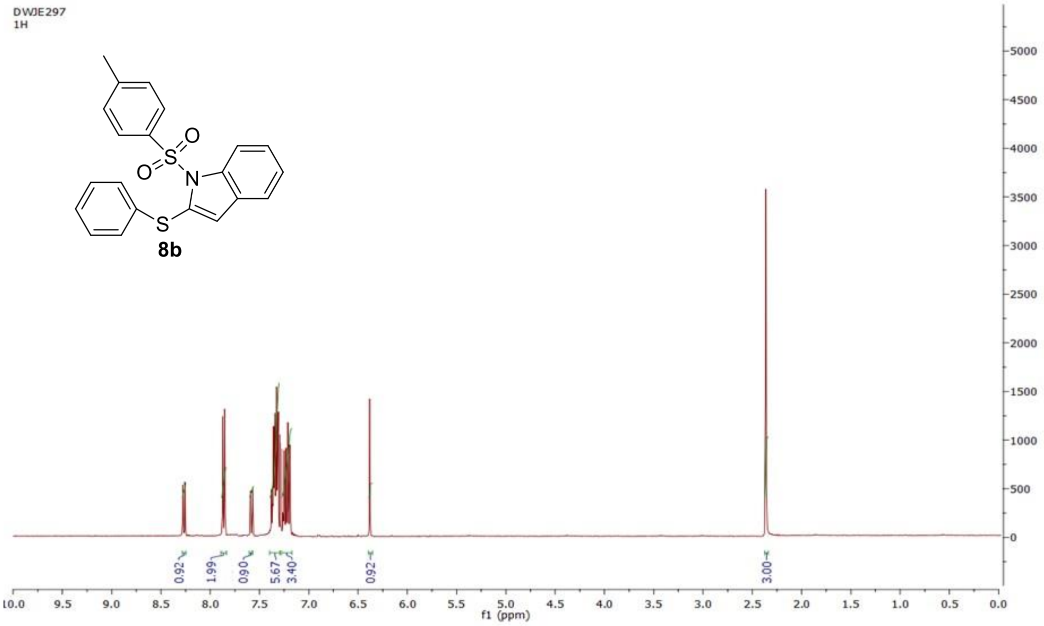
DWJE297
1H
8b
0.92
1.99
0.90
5.67
3.40
0.92
3.00
f1 (ppm)

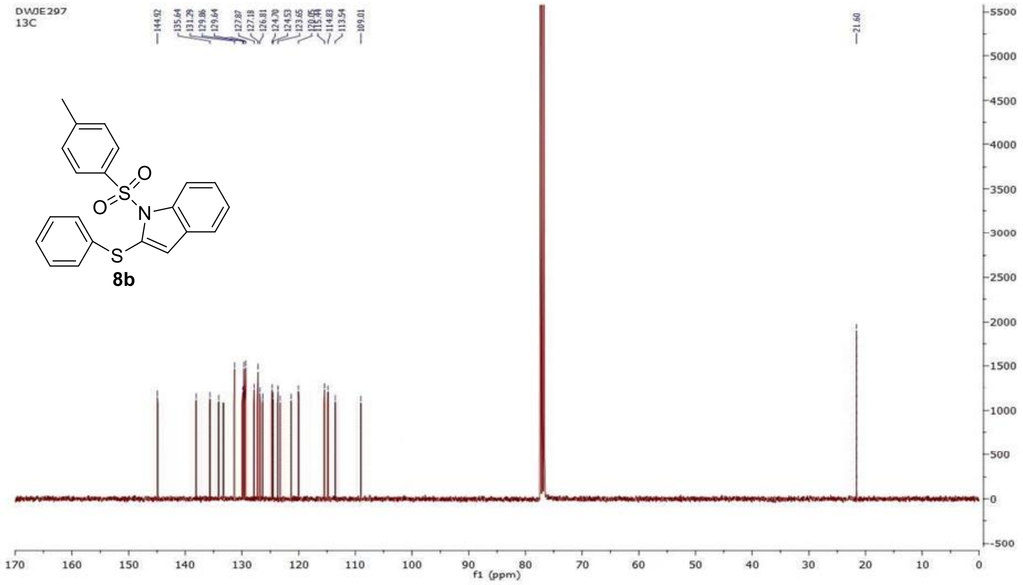
DWJE297
13C
144.92
135.64
131.29
129.86
129.64
127.87
127.18
126.81
124.70
124.53
123.65
114.83
113.54
109.01
21.60
8b
f1 (ppm)

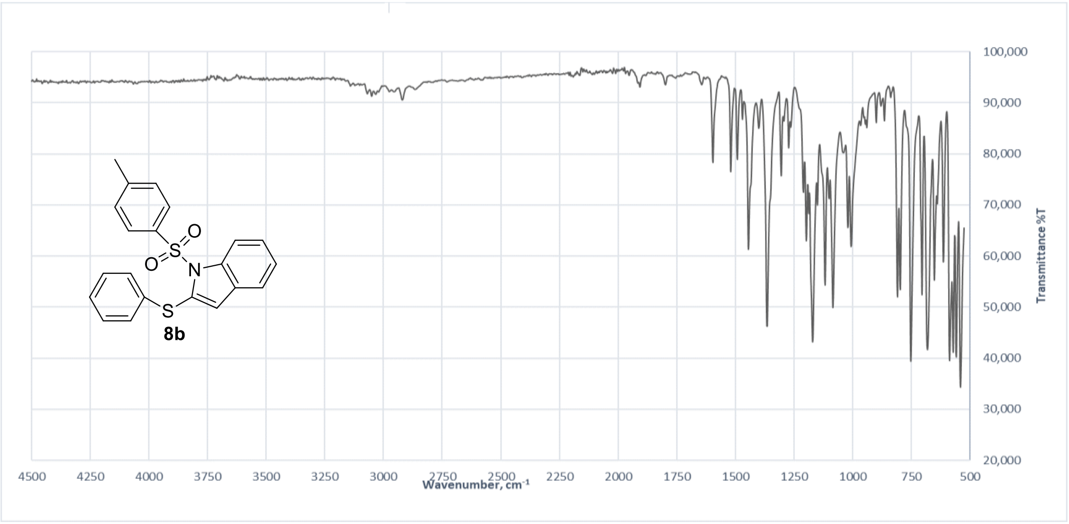
8b
Wavenumber, cm⁻¹
Transmittance %T

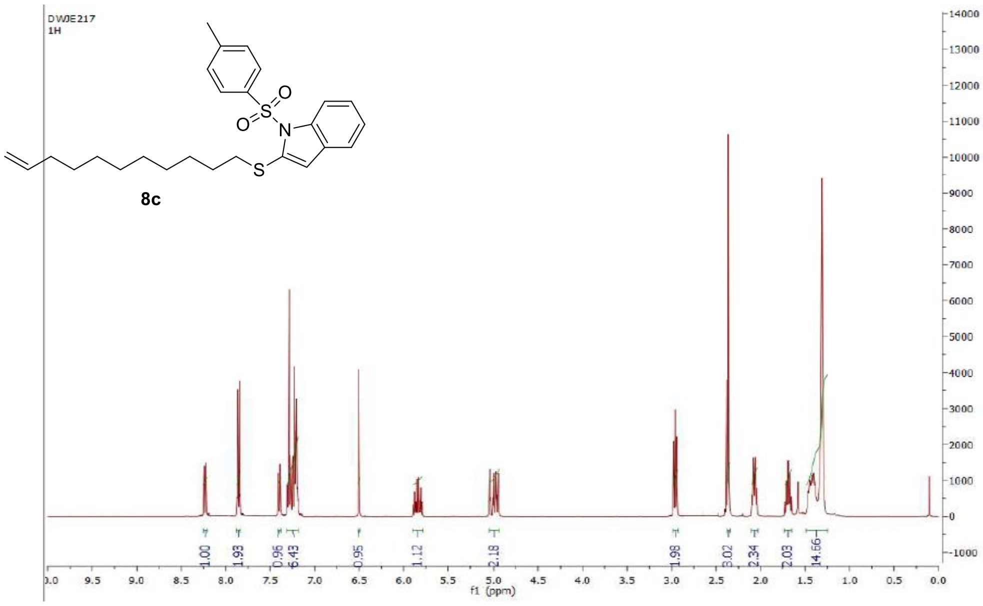
DWJE217
1H
8c
1.00
1.93
0.96
6.43
0.95
1.12
2.18
1.98
3.02
2.34
2.03
14.66
f1 (ppm)

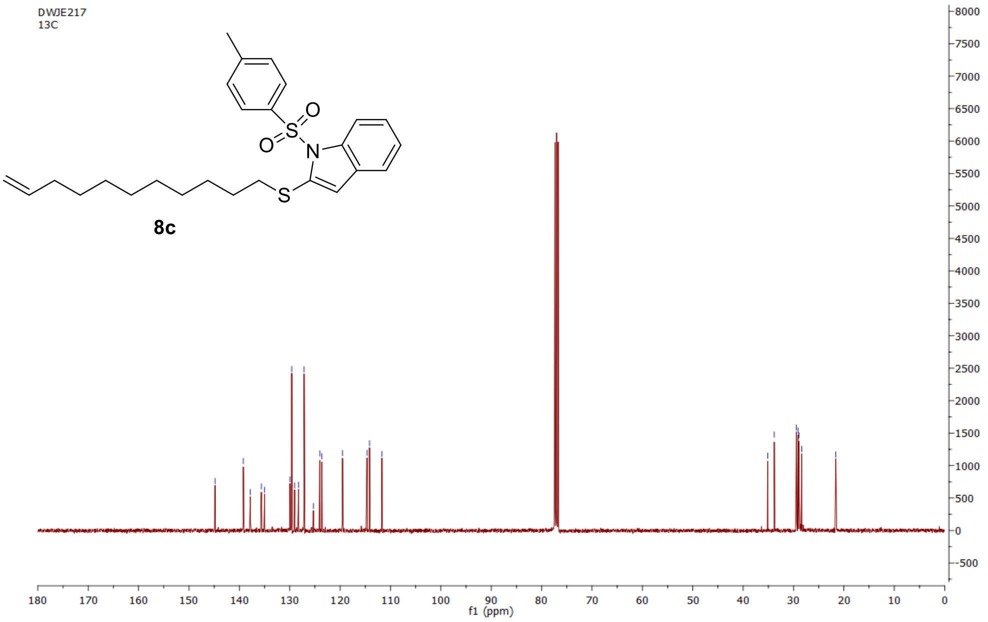
DWJE217
13C
8c
f1 (ppm)

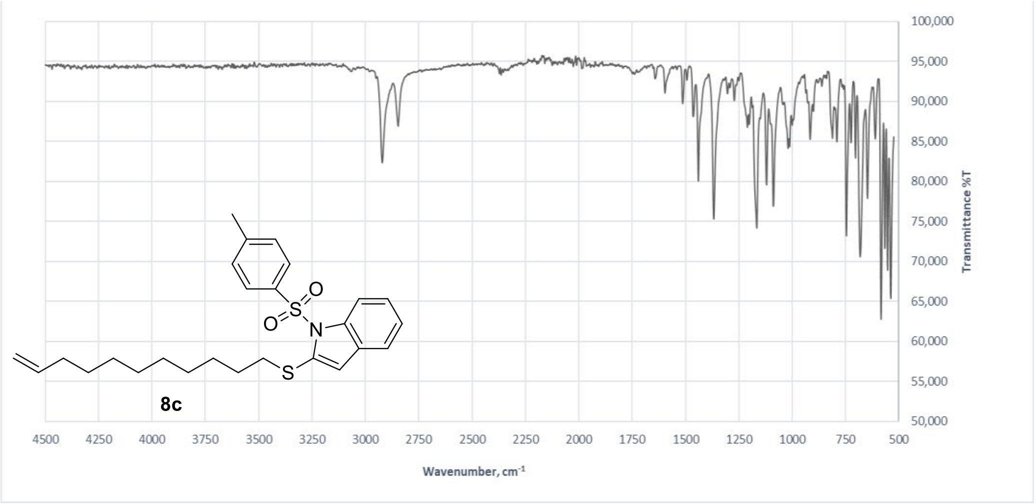
8c
Wavenumber, cm⁻¹
Transmittance %T

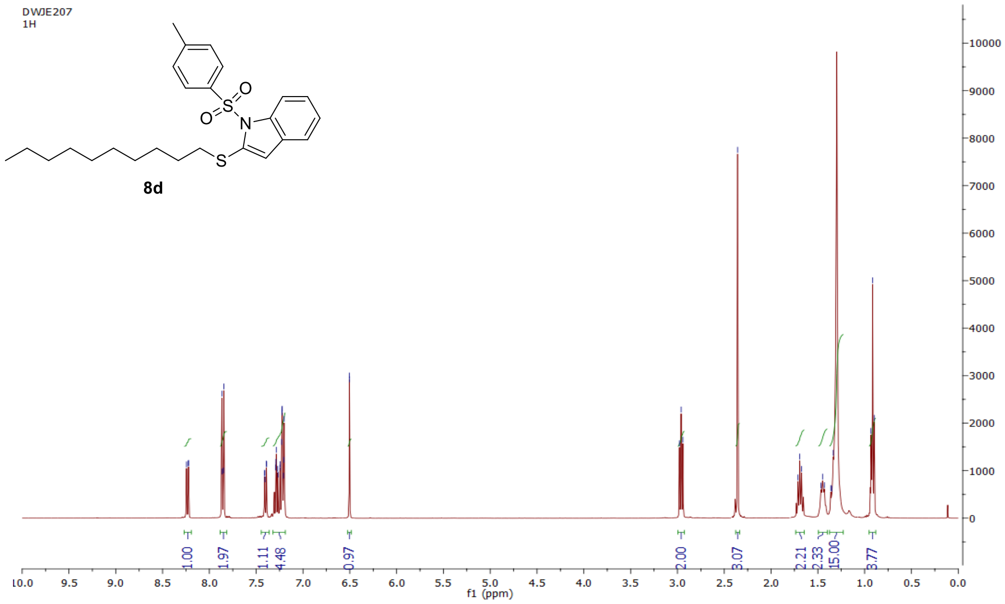
DWJE207
1H
8d
1.00
1.97
1.11
4.48
0.97
2.00
3.07
2.21
2.33
15.00
3.77
f1 (ppm)

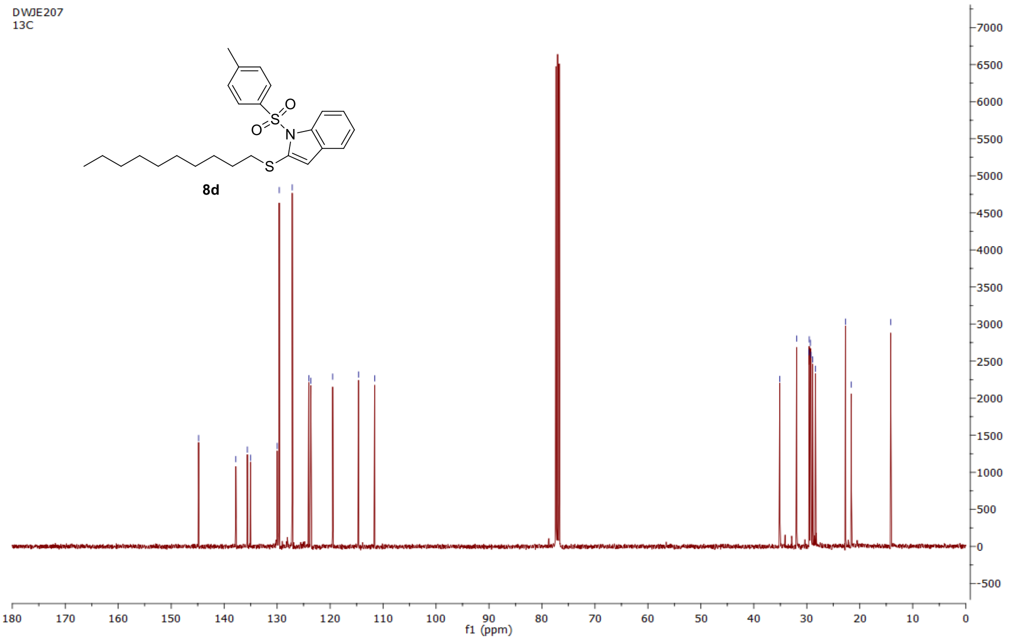
DWJE207
13C
8d
f1 (ppm)

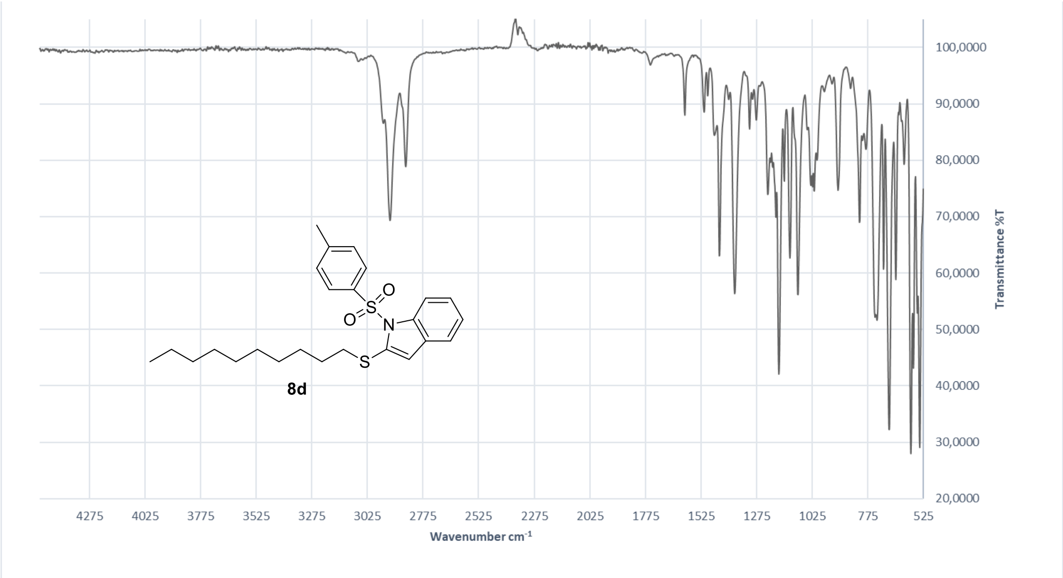
8d
Wavenumber cm-1
Transmittance %T

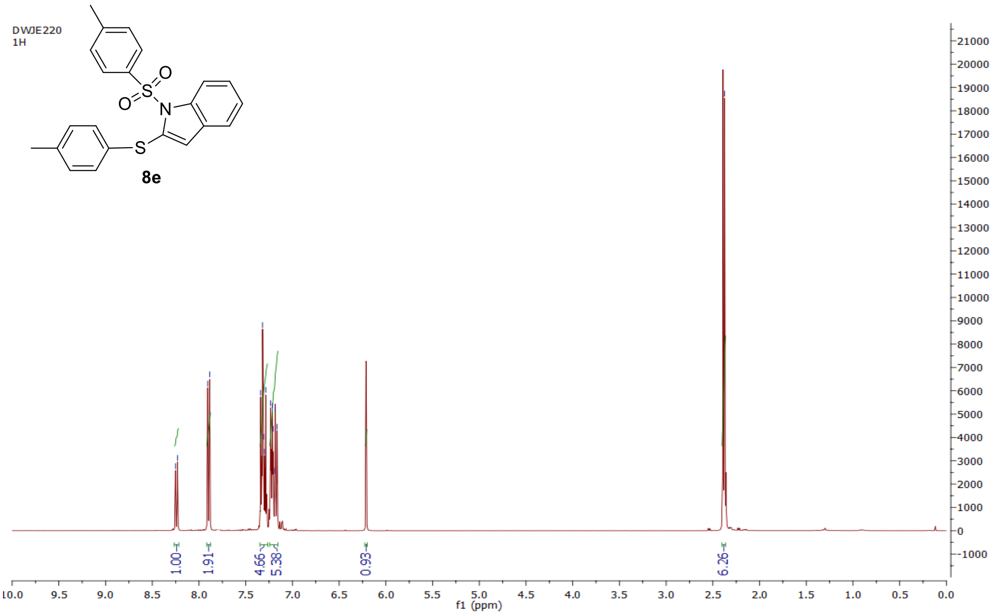
DWJE220
1H
8e
1.00
1.91
4.66
5.38
0.93
6.26
f1 (ppm)

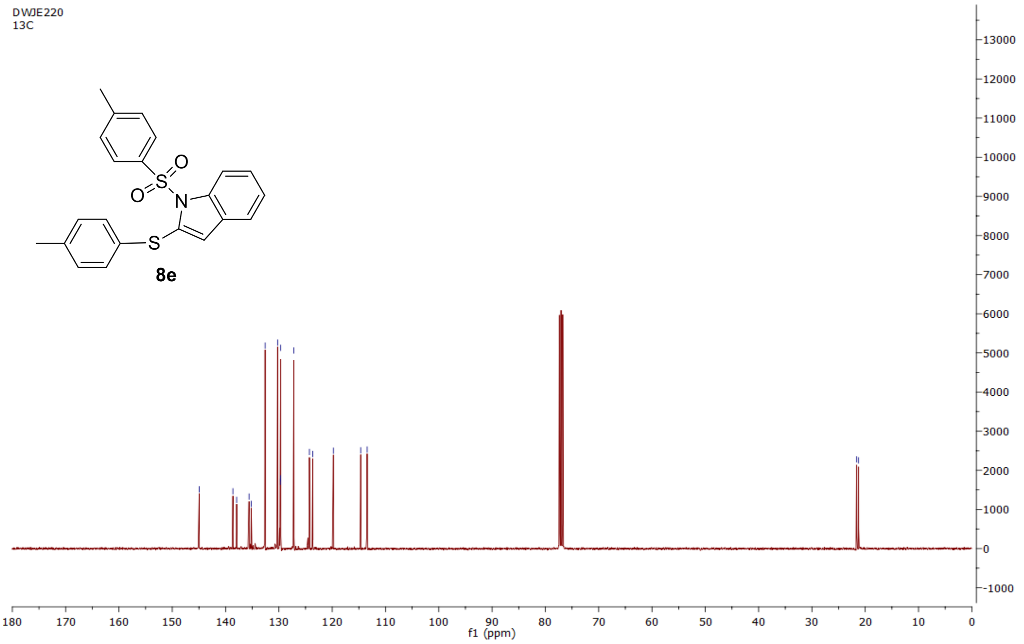
DWJE220
13C
8e
f1 (ppm)

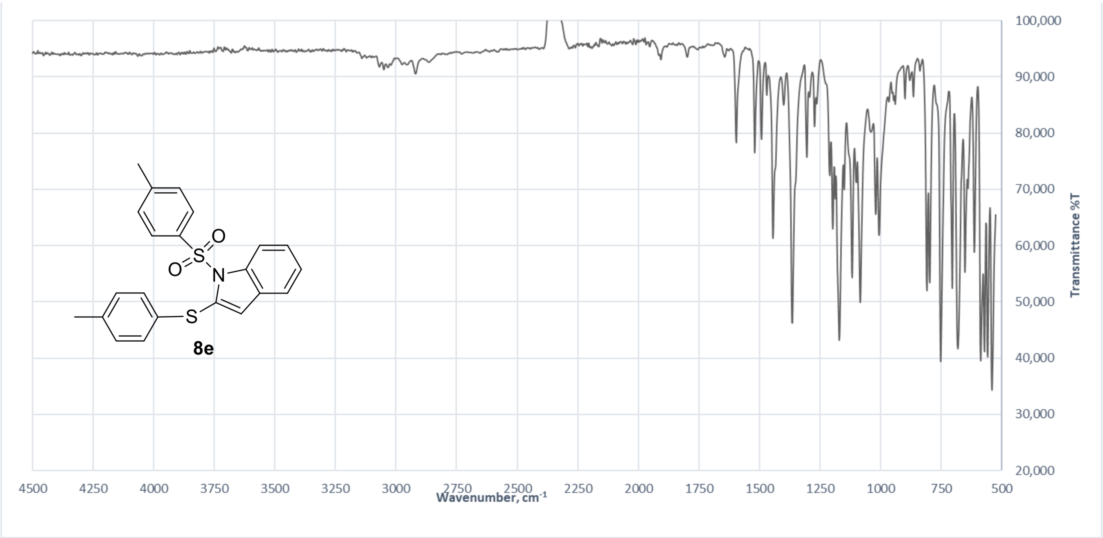
8e
Wavenumber, cm-1
Transmittance %T

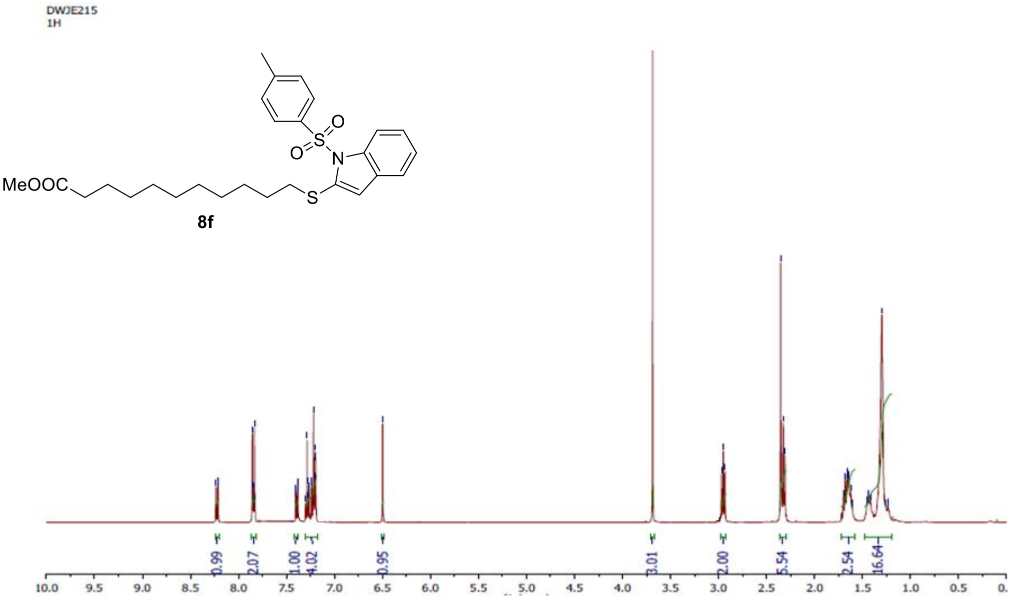
DWJE215
1H
MeOOC
8f

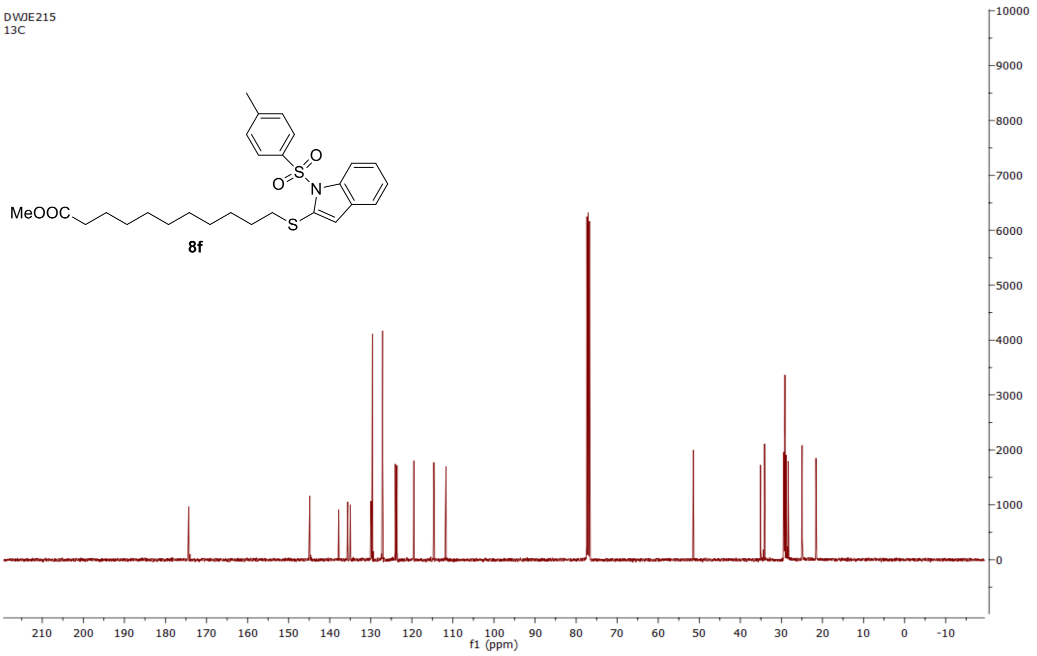
DWJE215
13C
MeOOC
S
N
O
S
O
8f
f1 (ppm)

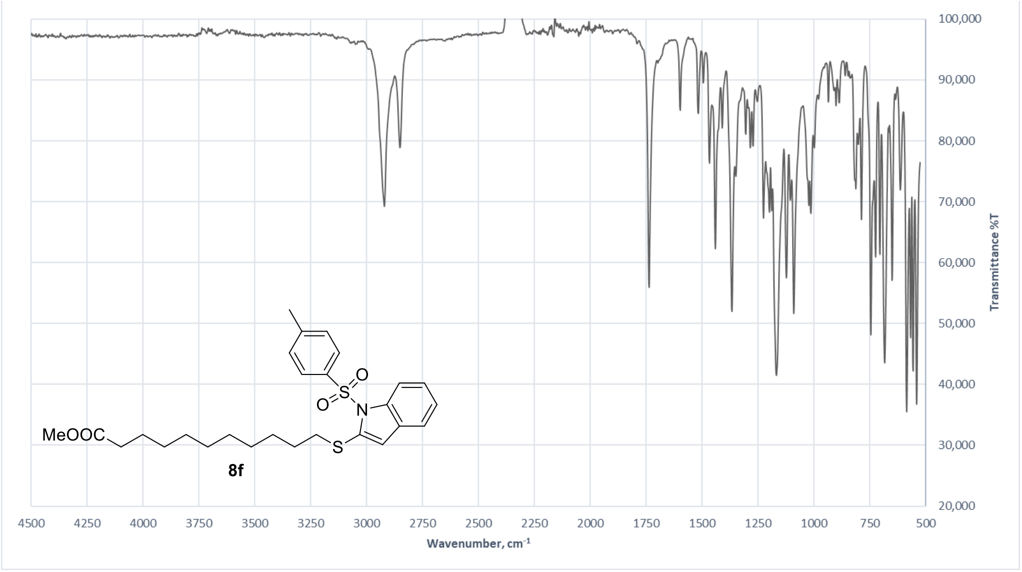
MeOOC
8f
Transmittance %T
Wavenumber, cm-1

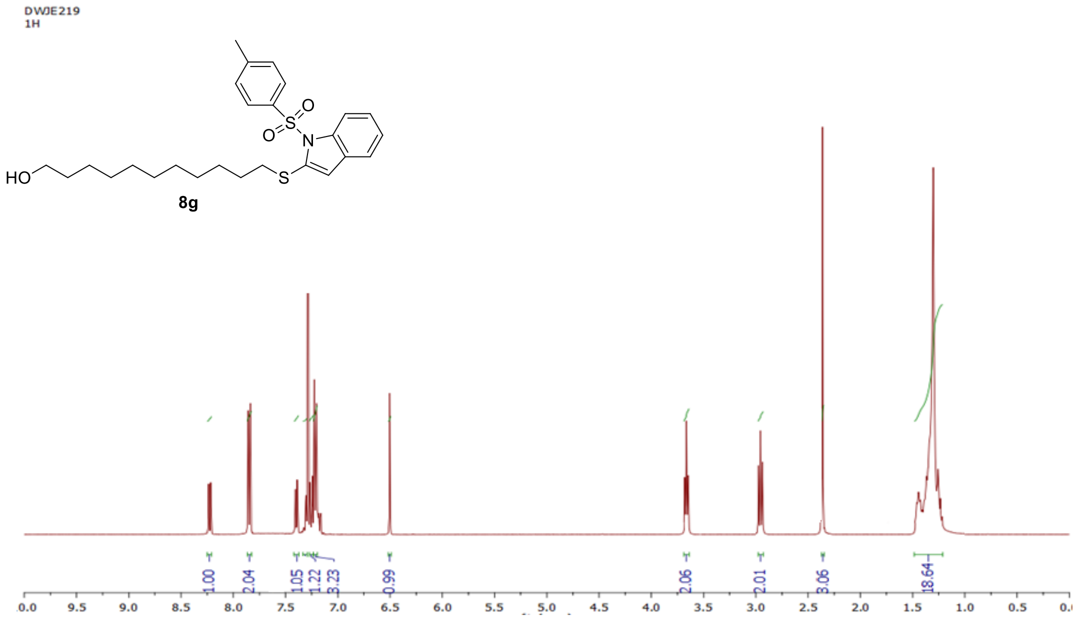
DWJE219
1H
HO
8g
1.00
2.04
1.05
1.22
3.23
0.99
2.06
2.01
3.06
18.64

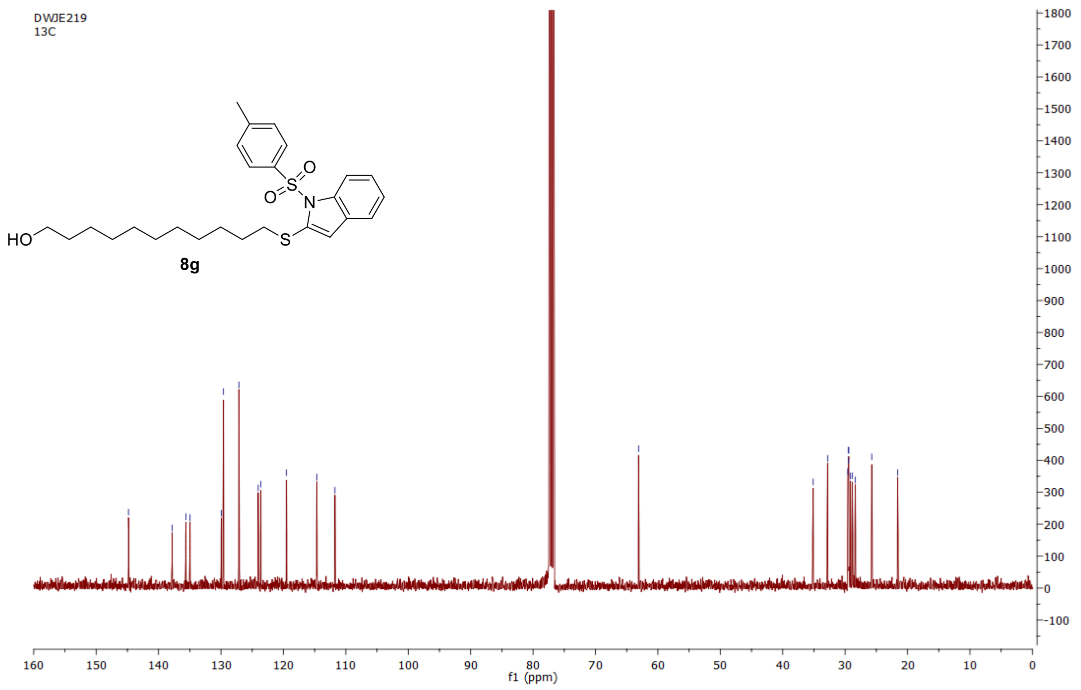
DWJE219
13C
HO
S
N
O
S
O
8g
f1 (ppm)

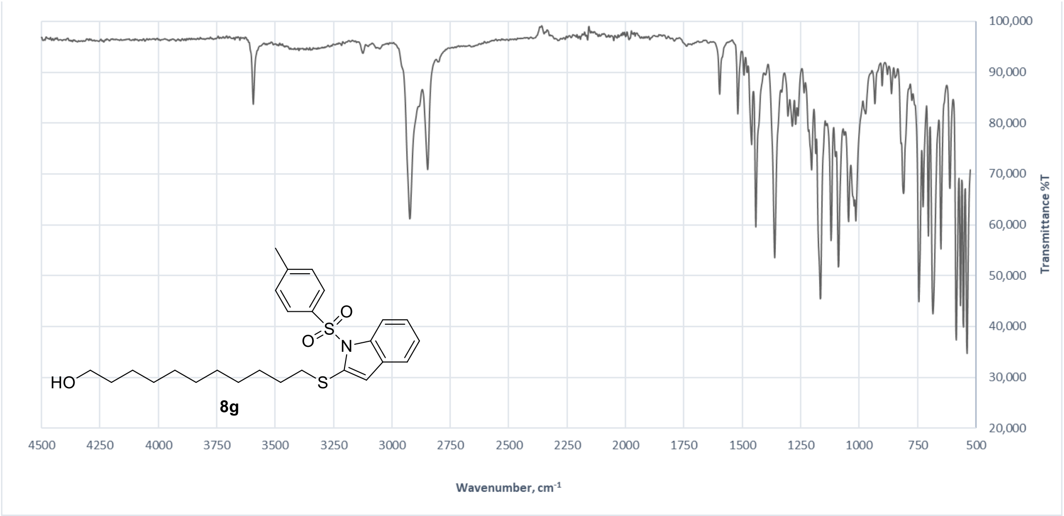
HO
S
N
O
S
O
8g
Transmittance %T
Wavenumber, cm⁻¹

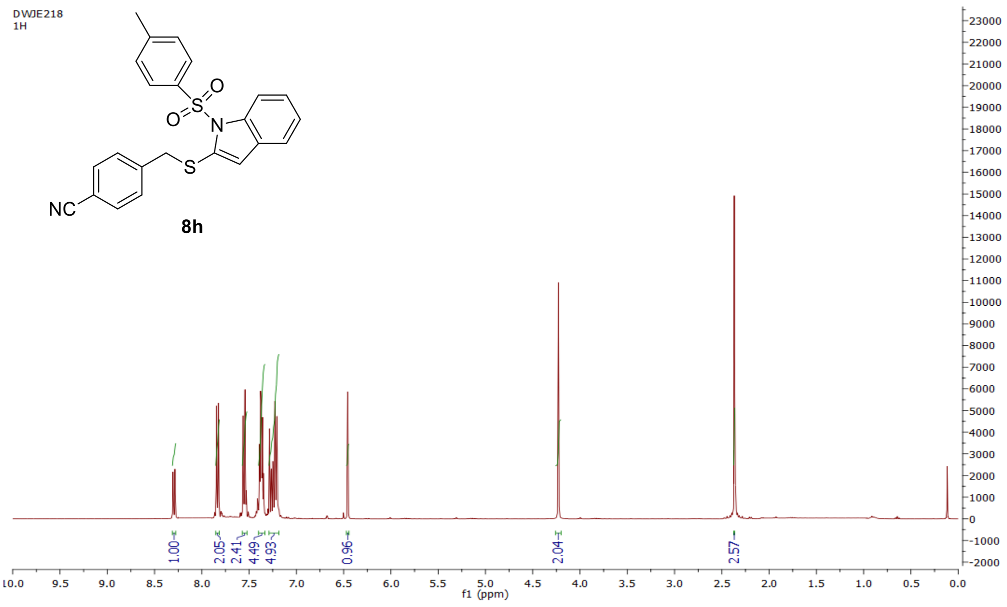
DWJE218
1H
NC
S
N
O
S
O
8h
f1 (ppm)

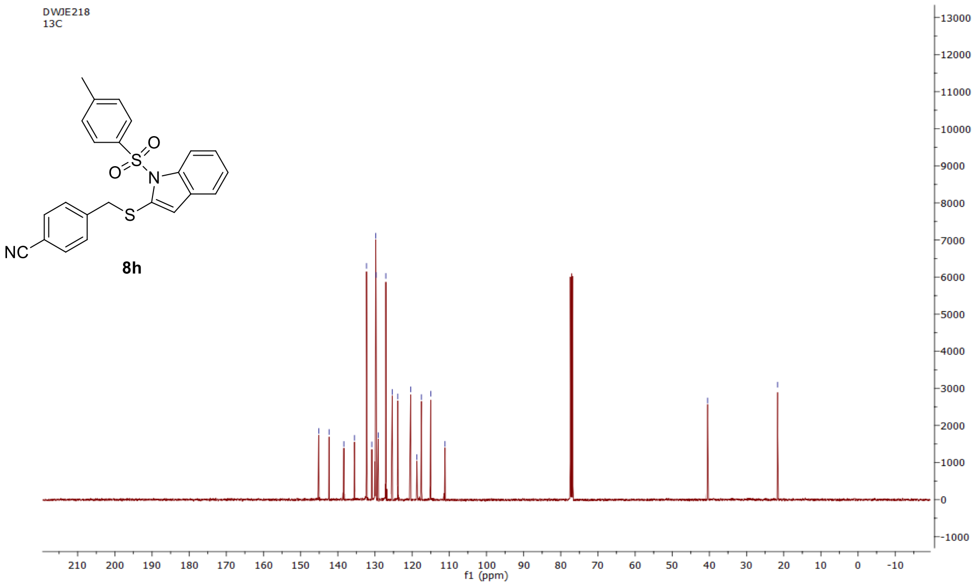
DWJE218
13C
NC
O
S
N
8h
f1 (ppm)

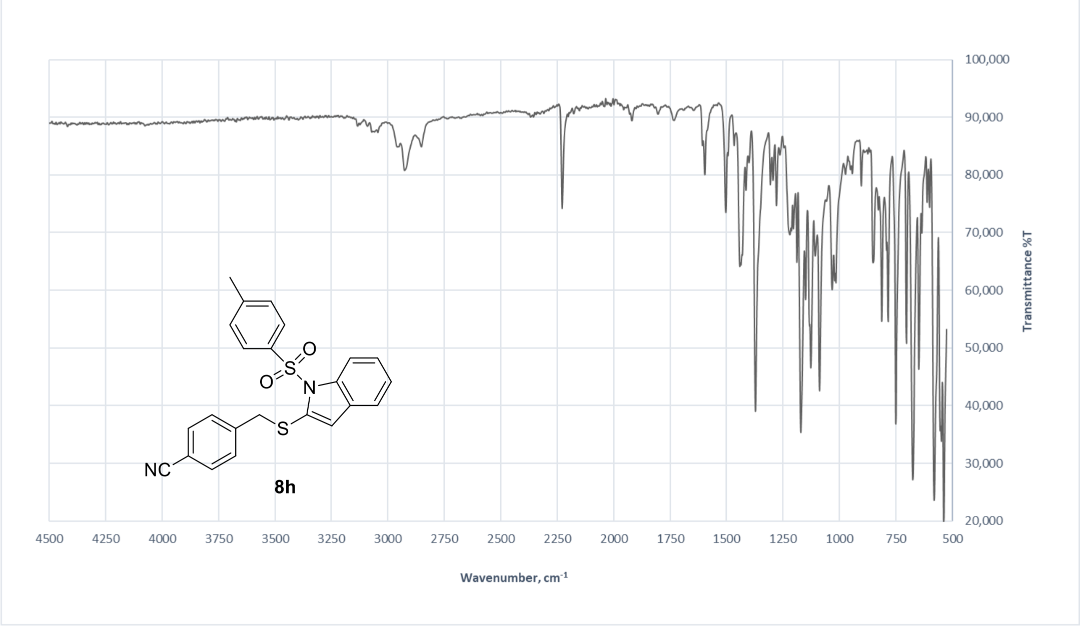
Transmittance %T
Wavenumber, cm-1
NC
8h

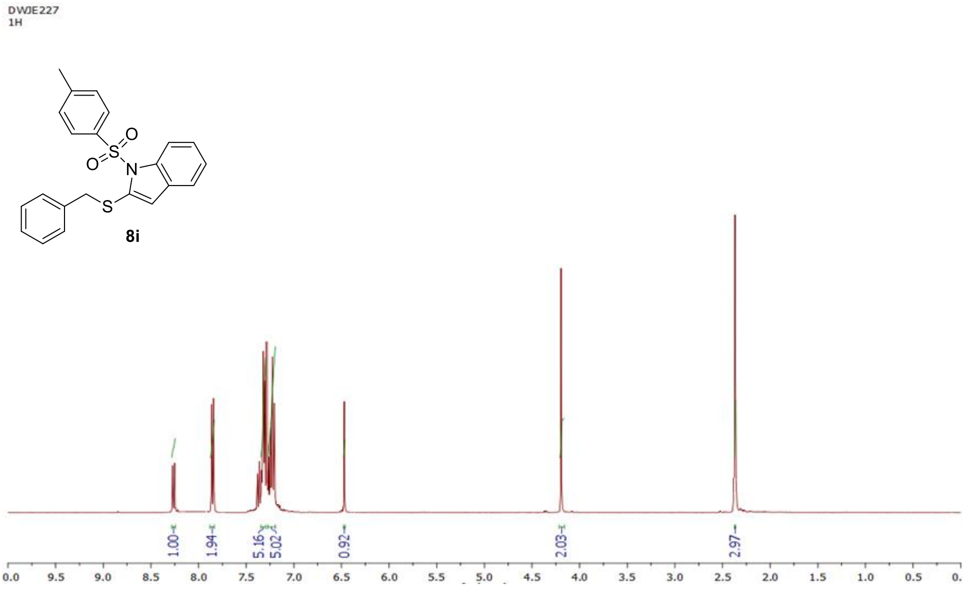
DWJE227
1H
8i

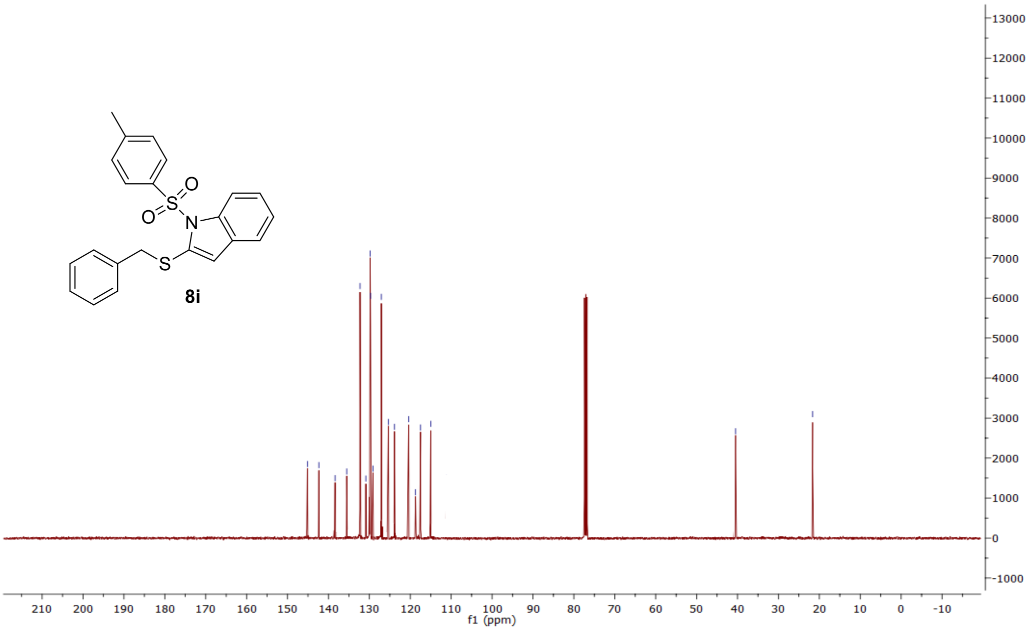
8i
f1 (ppm)

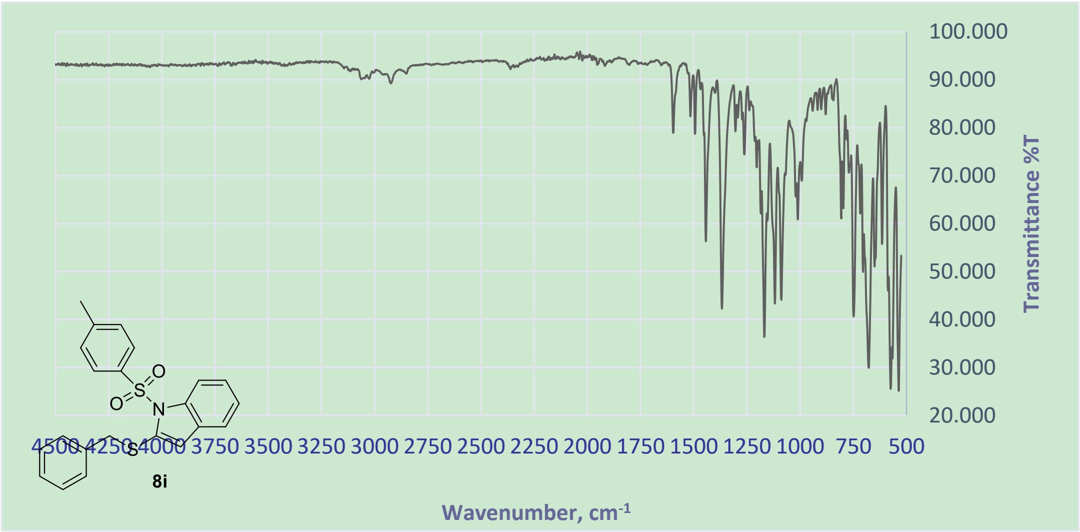
8i
Transmittance %T
Wavenumber, cm-1

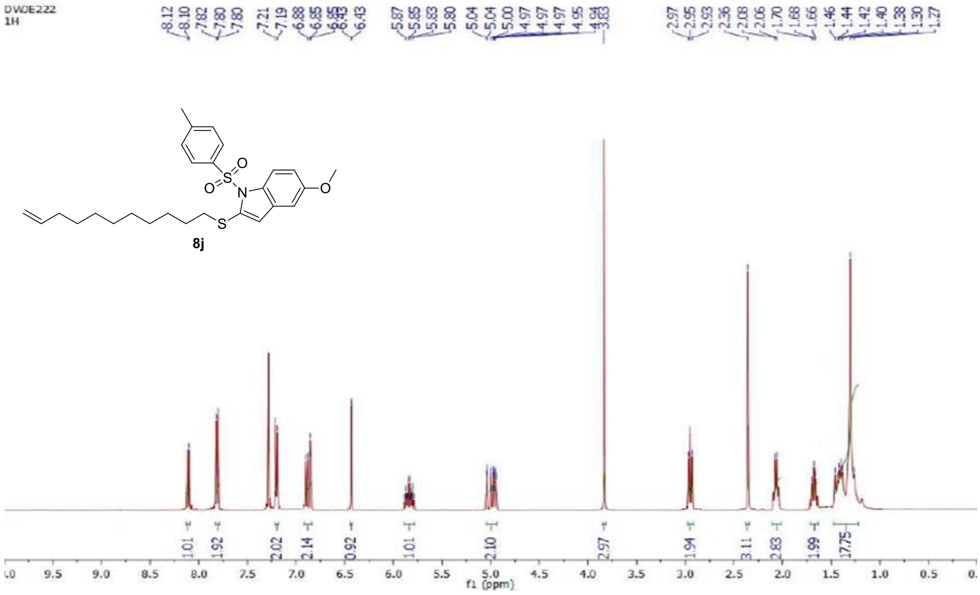
DWJE222
1H
8j
f1 (ppm)

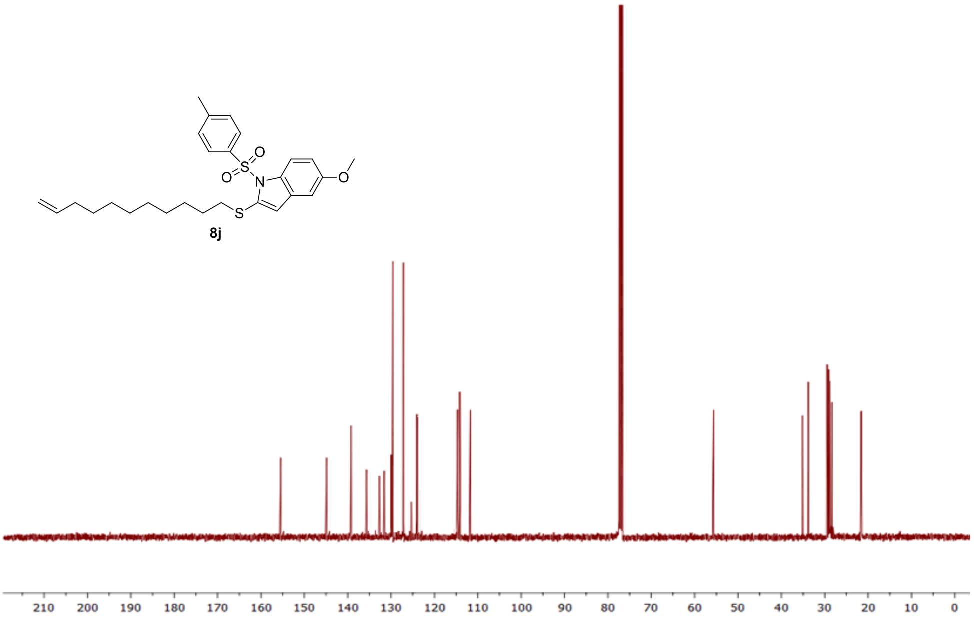

O
S
O
N
O
S
8j
210 200 190 180 170 160 150 140 130 120 110 100 90 80 70 60 50 40 30 20 10 0

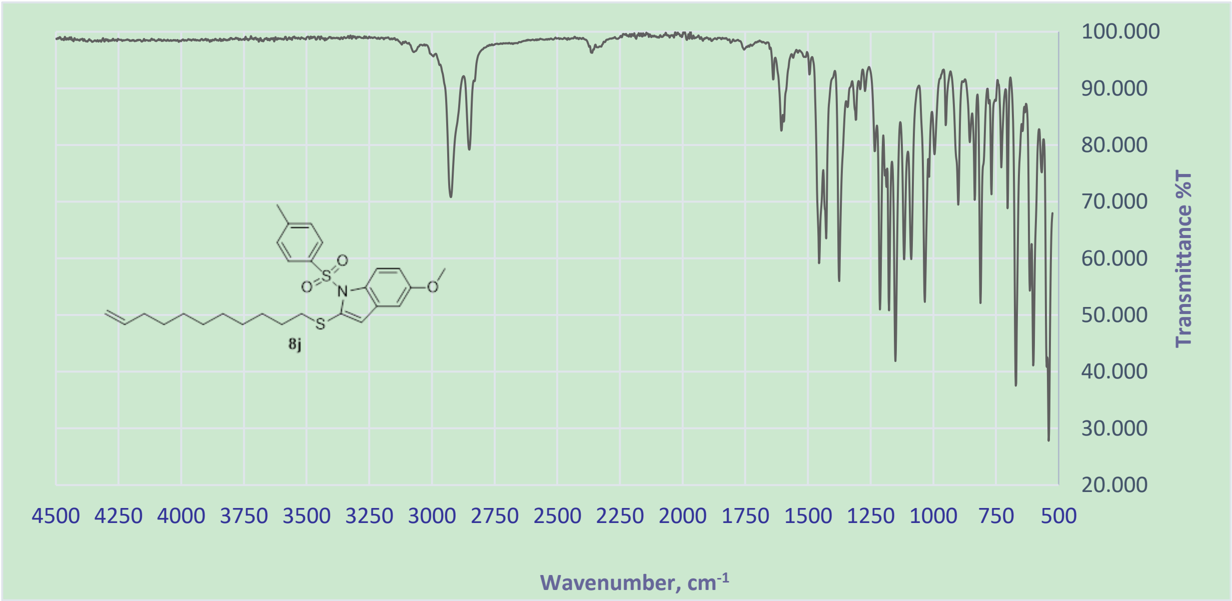

8j
100.000
90.000
80.000
70.000
60.000
50.000
40.000
30.000
20.000
Transmittance %T
4500 4250 4000 3750 3500 3250 3000 2750 2500 2250 2000 1750 1500 1250 1000 750 500
Wavenumber, cm-1

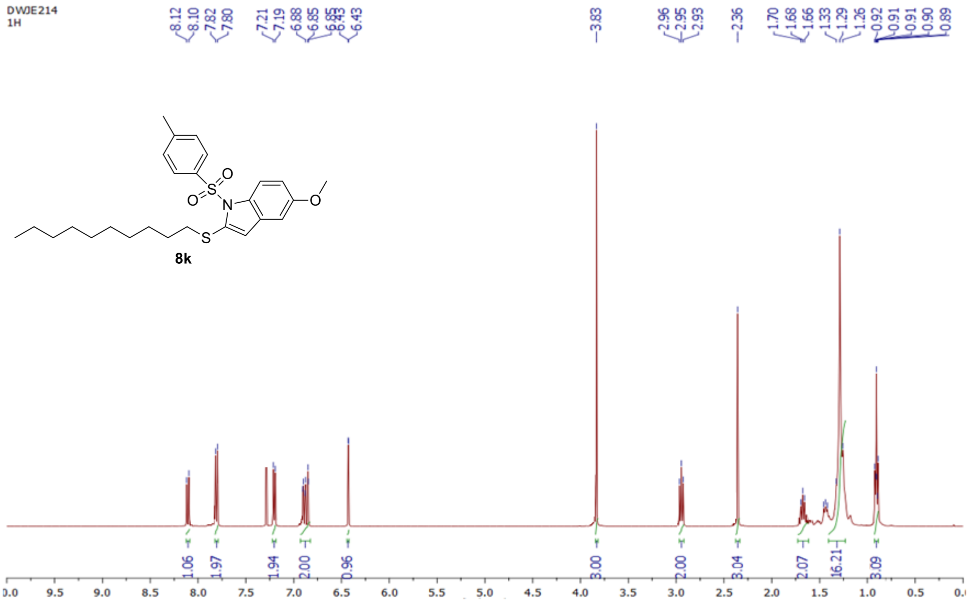

DWJE214
1H
8.12 8.10 7.82 7.80 7.21 7.19 6.88 6.85 6.43
3.83
2.96 2.95 2.93
2.36
1.70 1.68 1.66 1.33 1.29 1.26 0.92 0.91 0.91 0.90 0.89
8k
1.06 1.97 1.94 2.00 0.96 3.00 2.00 3.04 2.07 16.21 3.09
9.5 9.0 8.5 8.0 7.5 7.0 6.5 6.0 5.5 5.0 4.5 4.0 3.5 3.0 2.5 2.0 1.5 1.0 0.5

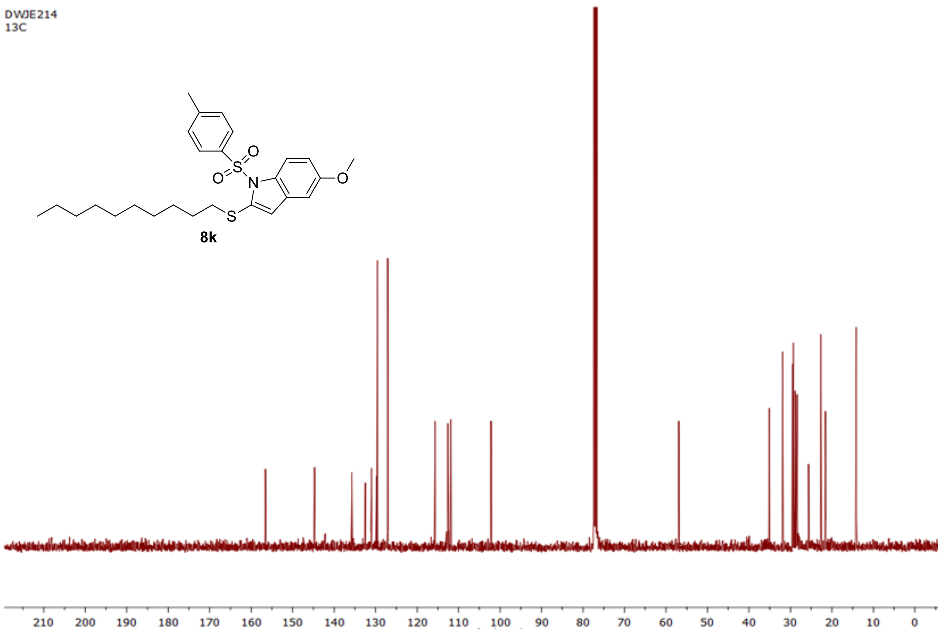
DWJE214
13C
O
S
O
N
O
S
8k
210
200
190
180
170
160
150
140
130
120
110
100
90
80
70
60
50
40
30
20
10
0

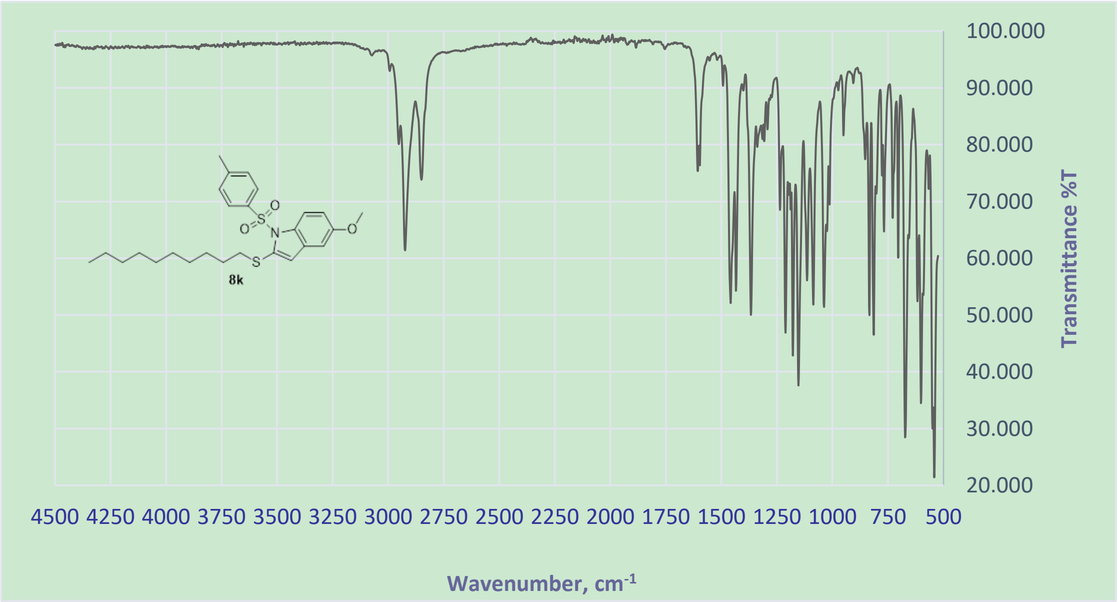
100.000
90.000
80.000
70.000
60.000
50.000
40.000
30.000
20.000
Transmittance %T
8k
4500 4250 4000 3750 3500 3250 3000 2750 2500 2250 2000 1750 1500 1250 1000 750 500
Wavenumber, cm-1

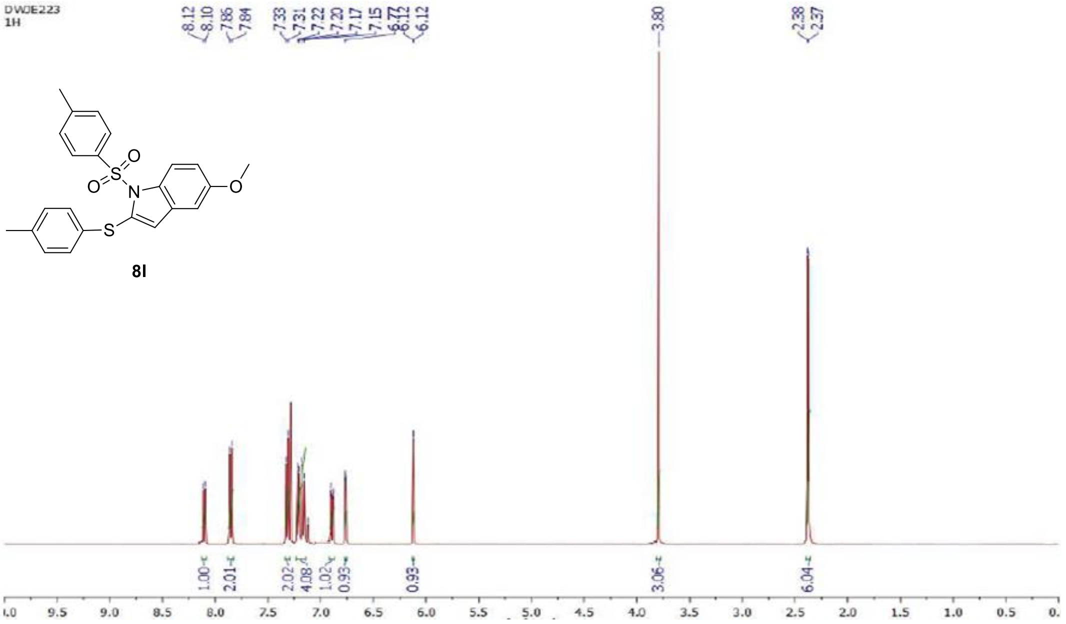
DWJE223
1H
8.12
8.10
7.86
7.84
7.33
7.31
7.22
7.20
7.17
7.15
6.77
6.12
3.80
2.38
2.37
8l
1.00
2.01
2.02
4.08
1.02
0.93
0.93
3.06
6.04

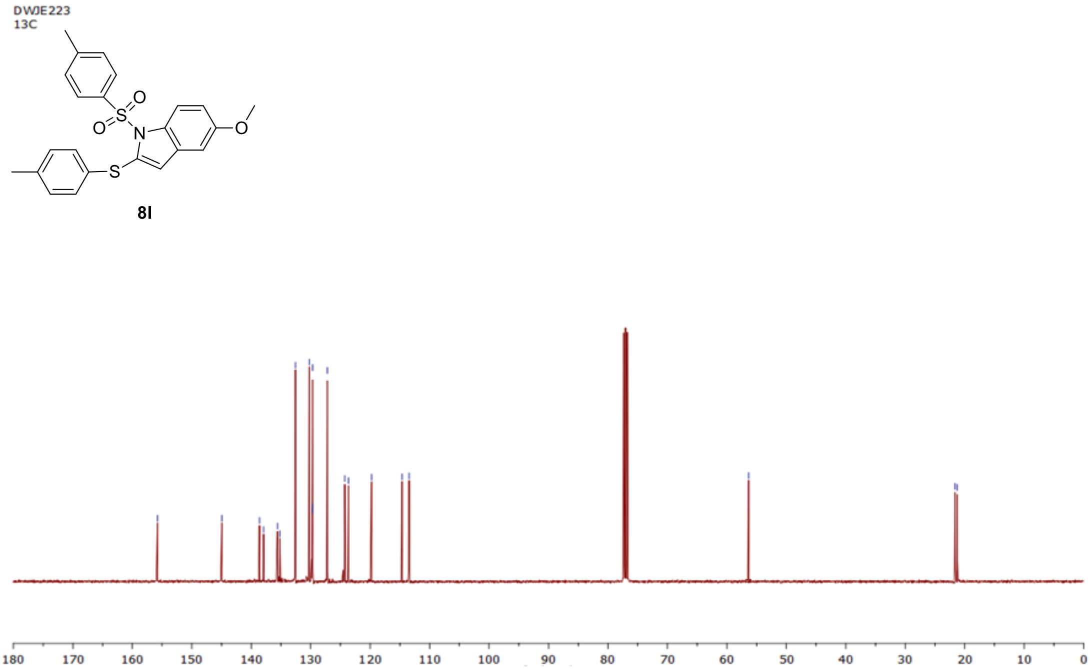
DWJE223
13C
8l
180
170
160
150
140
130
120
110
100
90
80
70
60
50
40
30
20
10
0

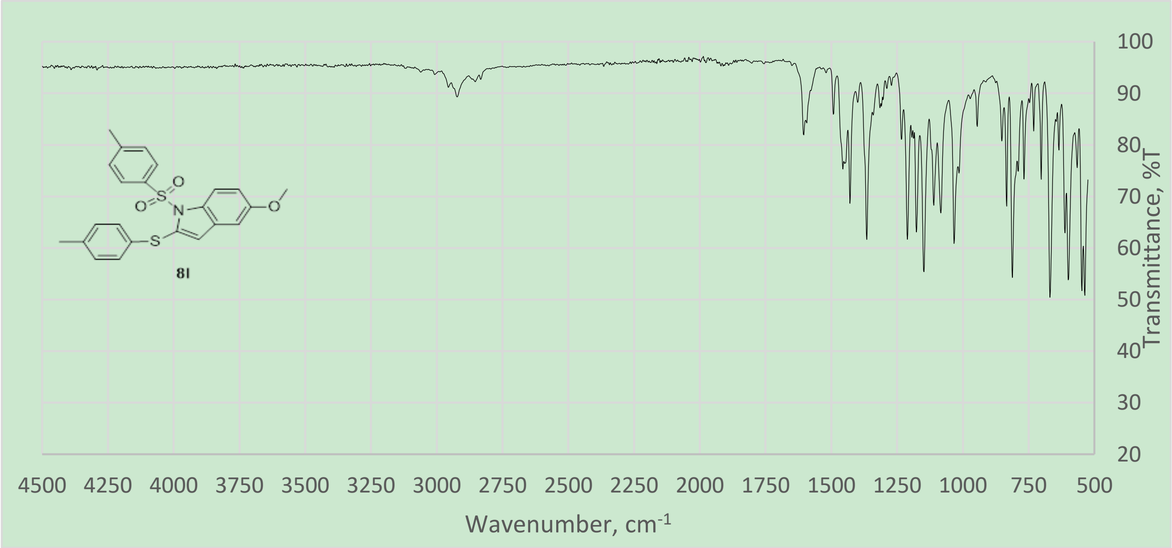
8l
Transmittance, %T
Wavenumber, cm-1

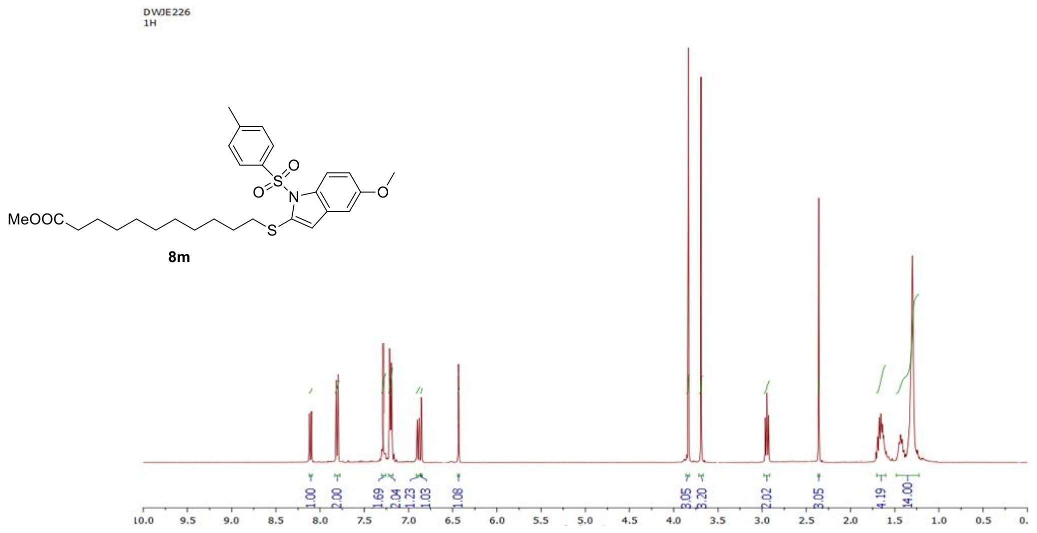
DWJE226
1H
MeOOC
S
N
O
S
O
O
8m
1.00
2.00
1.69
2.04
1.23
1.03
1.08
3.05
3.20
2.02
3.05
4.19
14.00

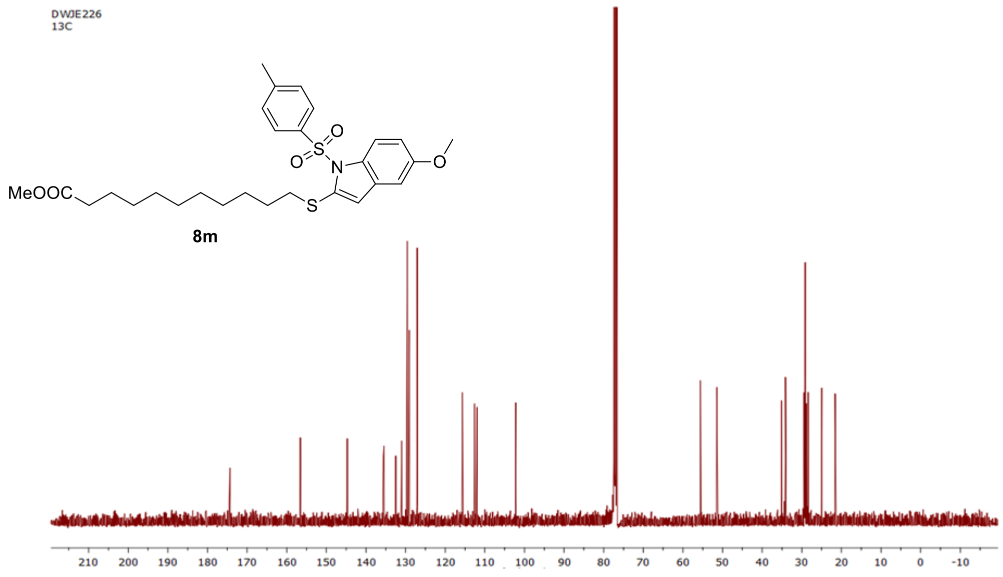
DWJE226
13C
MeOOC
S
N
O
S
O
O
8m

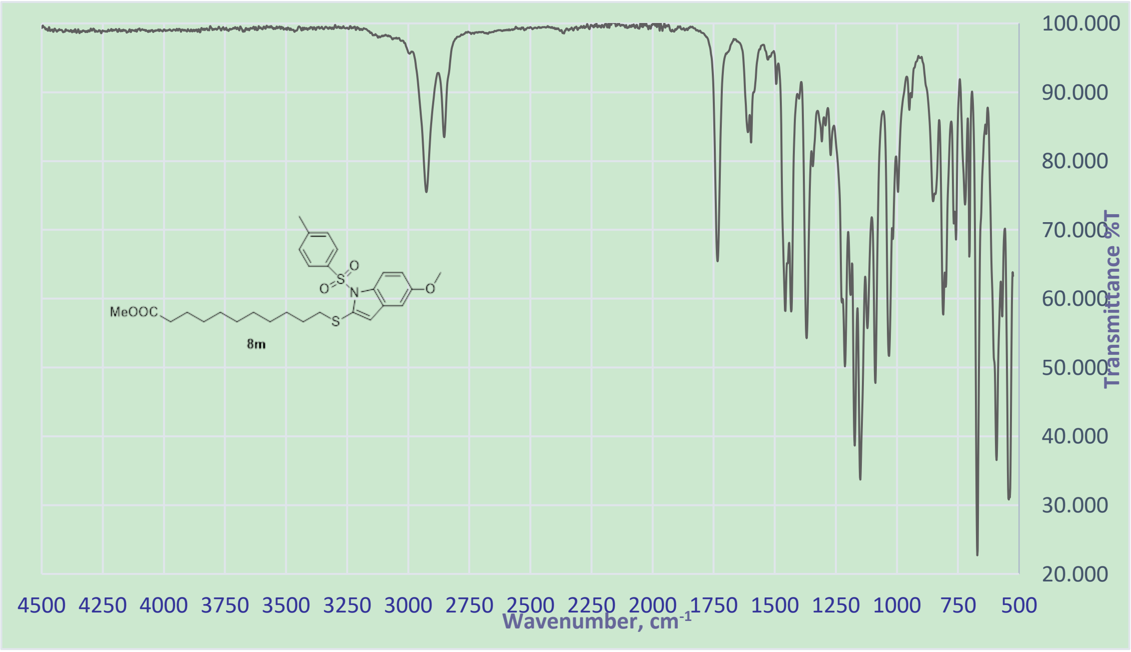
MeOOC
S
N
O
S
O
O
8m
Transmittance %T
Wavenumber, cm-1

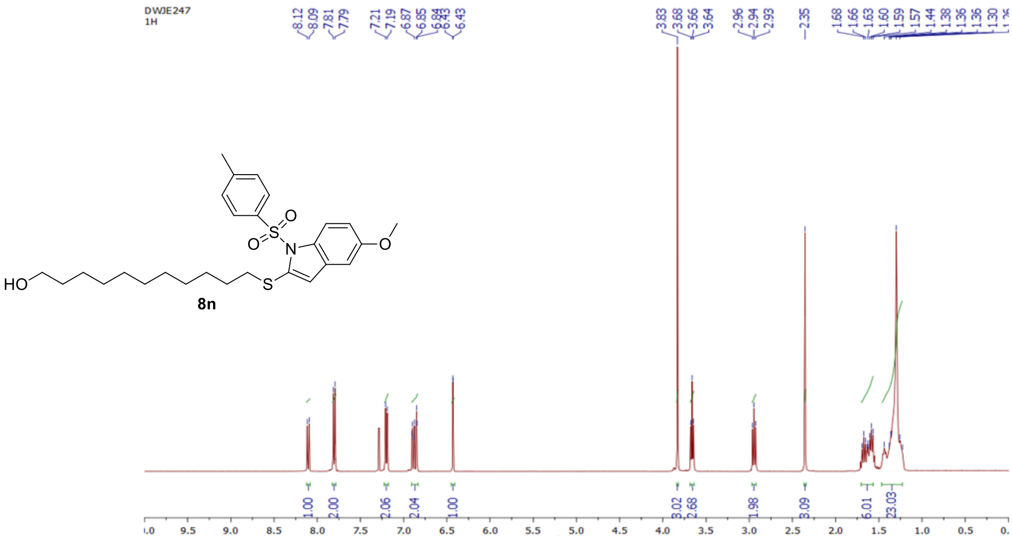
DWJE247
1H
HO
S
N
O
8n

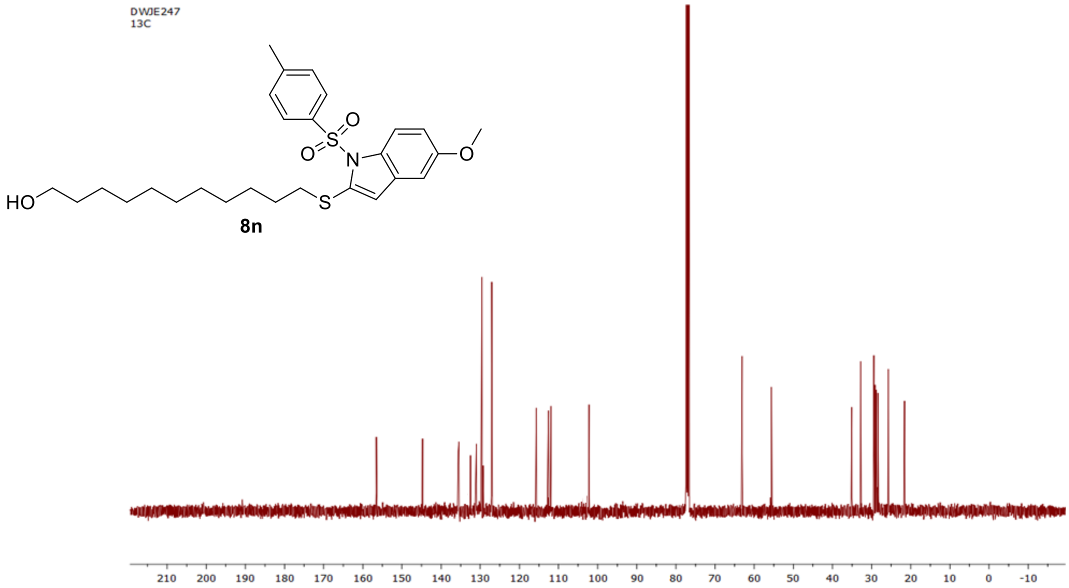
DWJE247
13C
HO
S
N
O
8n

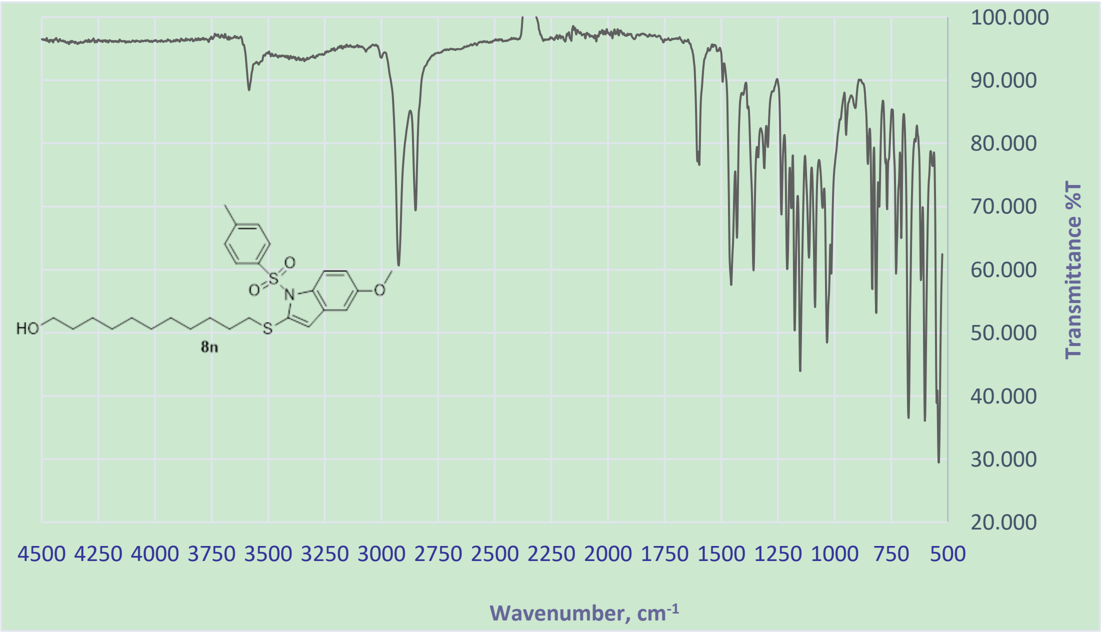
HO
S
N
O
8n
Transmittance %T
Wavenumber, cm-1

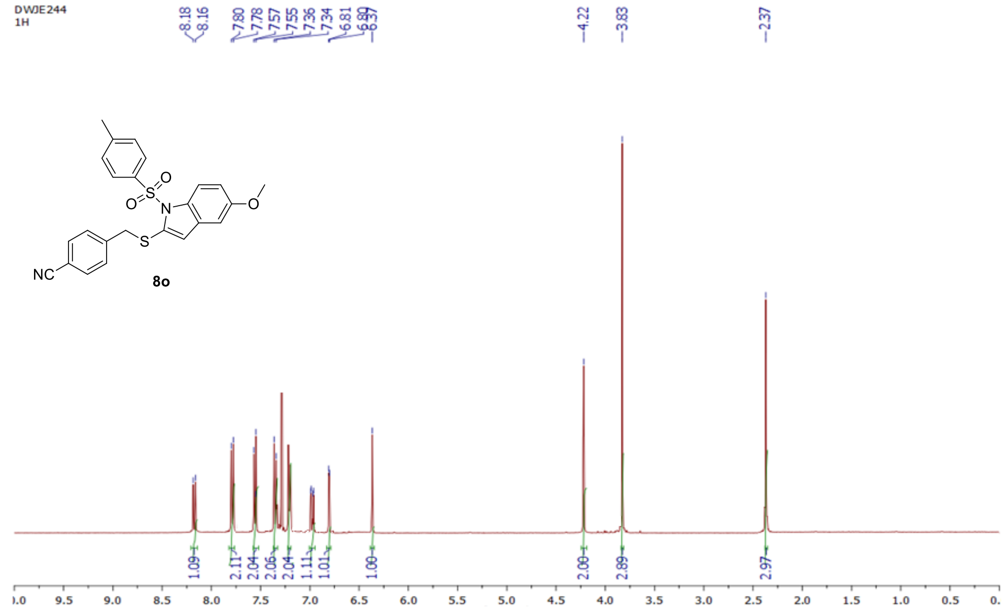
DWJE244
1H
8.18
8.16
7.80
7.78
7.57
7.55
7.36
7.34
6.81
6.80
6.37
4.22
3.83
2.37
NC
S
N
O
O
8o
1.09
2.11
2.04
2.06
2.04
1.11
1.01
1.00
2.00
2.89
2.97

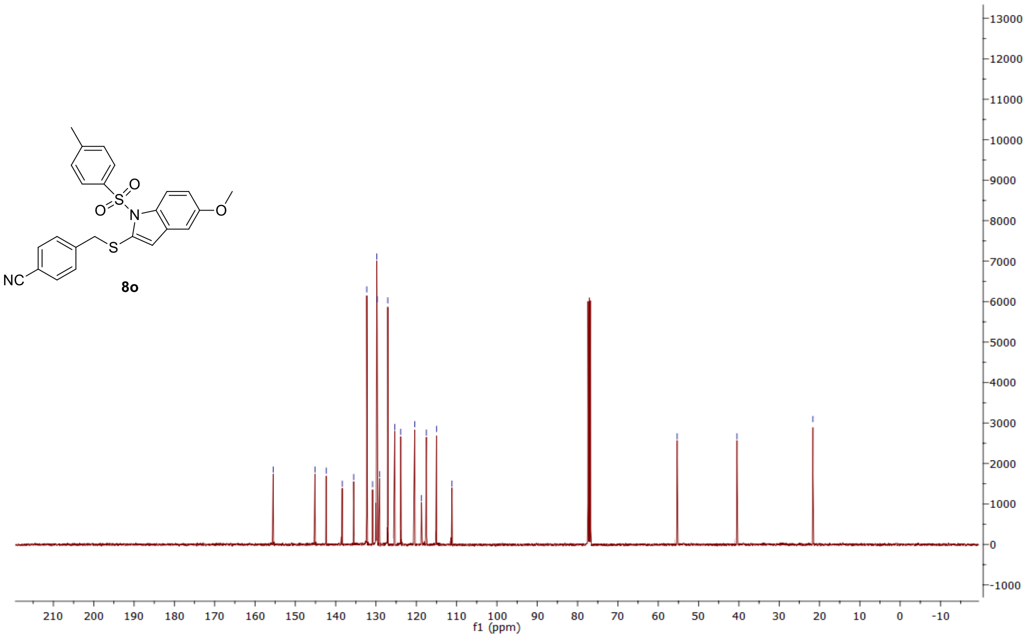
NC
S
N
O
O
8o
f1 (ppm)

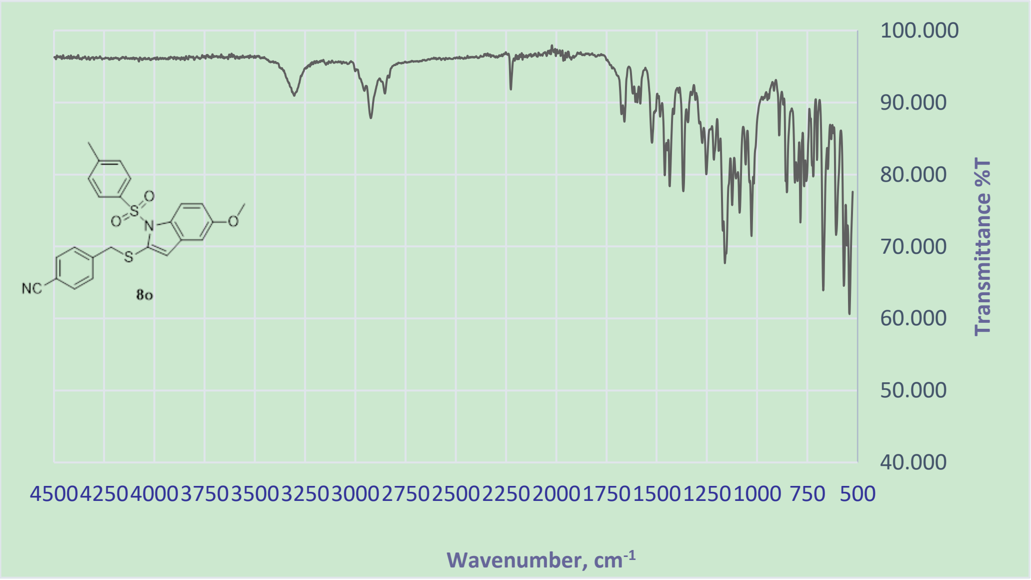
NC
S
N
O
O
8o
Transmittance %T
Wavenumber, cm-1

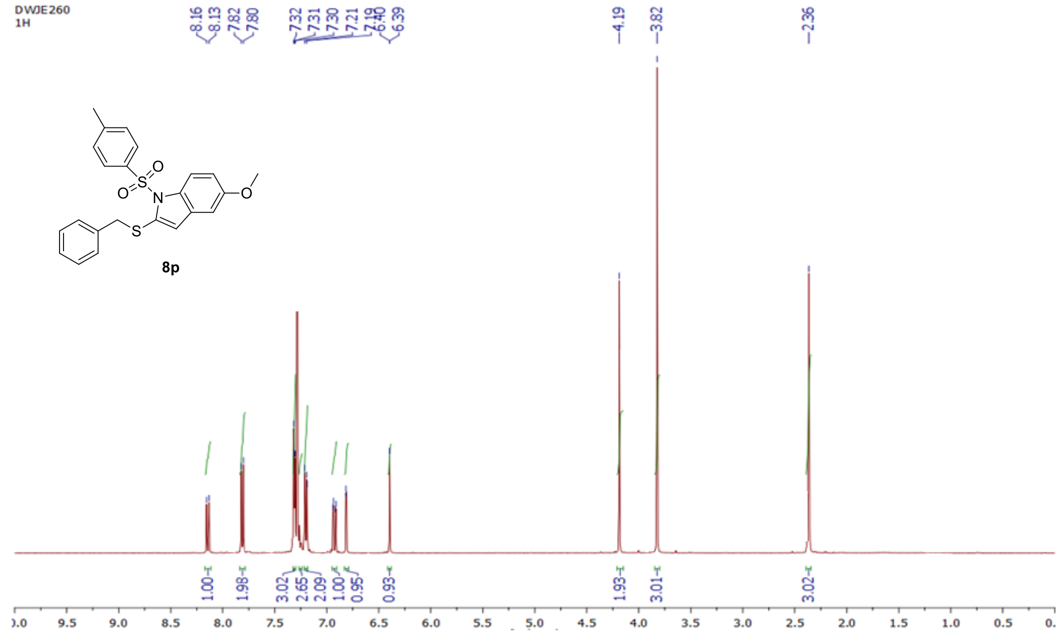

DWJE260
1H
8.16
8.13
7.82
7.80
7.32
7.31
7.30
7.21
7.19
6.40
6.39
4.19
3.82
2.36
8p
1.00
1.98
3.02
2.65
2.09
1.00
0.95
0.93
1.93
3.01
3.02
9.5
9.0
8.5
8.0
7.5
7.0
6.5
6.0
5.5
5.0
4.5
4.0
3.5
3.0
2.5
2.0
1.5
1.0
0.5

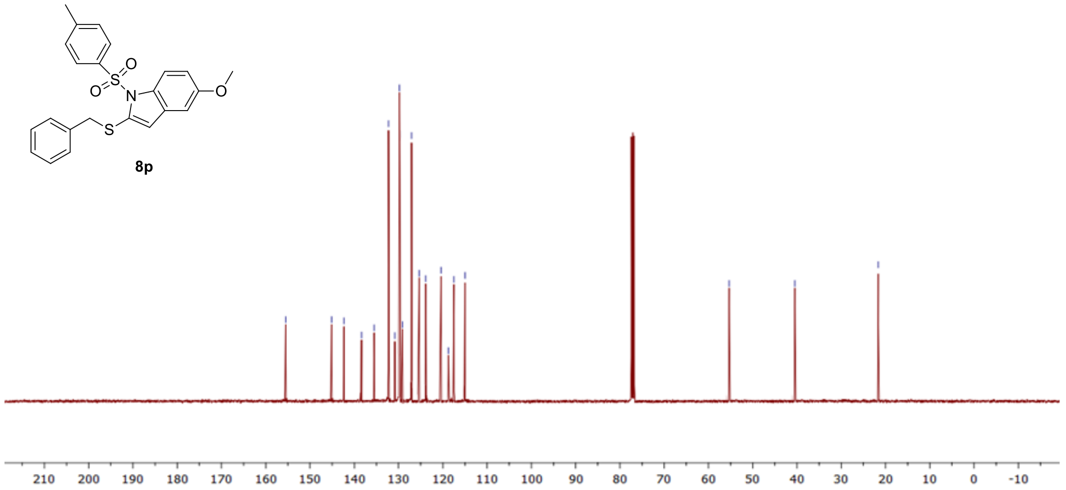

8p
210
200
190
180
170
160
150
140
130
120
110
100
90
80
70
60
50
40
30
20
10
0
-10

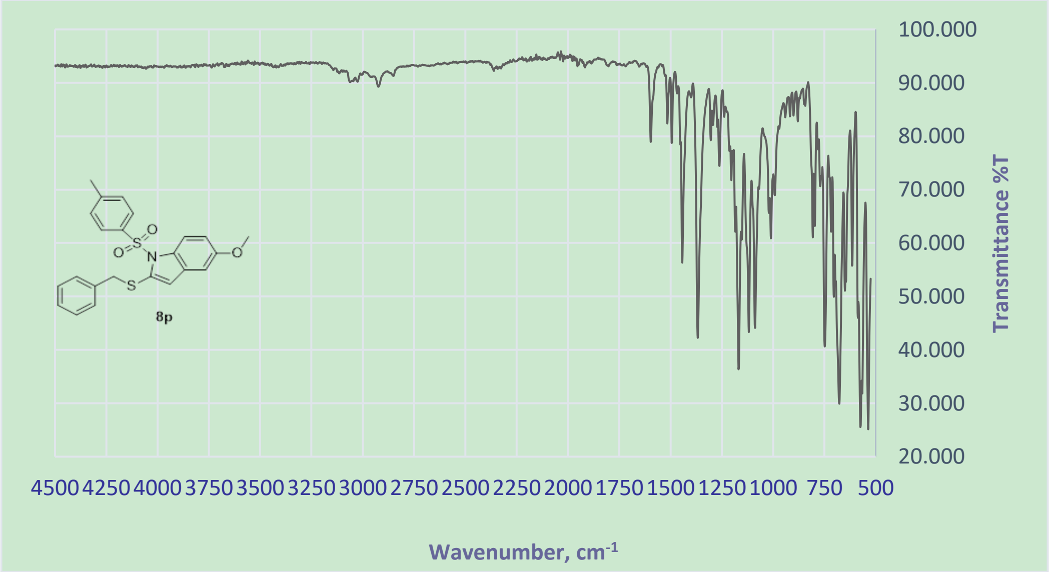

8p
100.000
90.000
80.000
70.000
60.000
50.000
40.000
30.000
20.000
Transmittance %T
4500 4250 4000 3750 3500 3250 3000 2750 2500 2250 2000 1750 1500 1250 1000 750 500
Wavenumber, cm-1

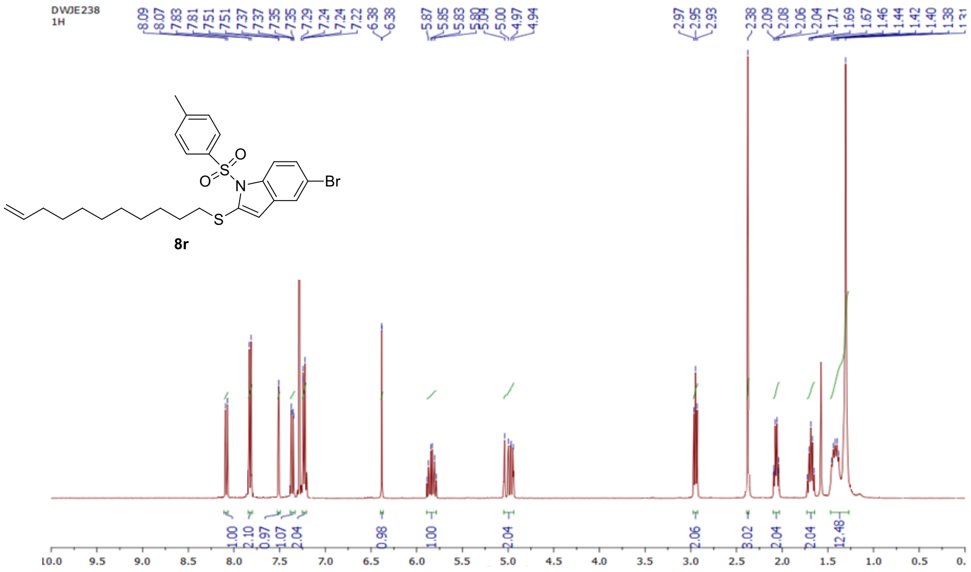
DWJE238
1H
Br
8r

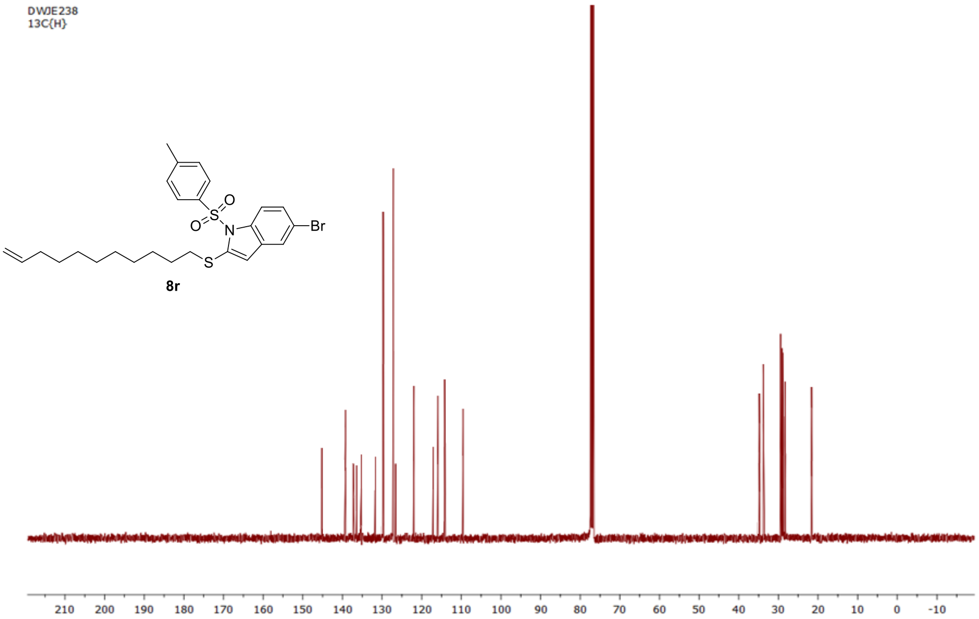
DWJE238
13C{H}
Br
8r

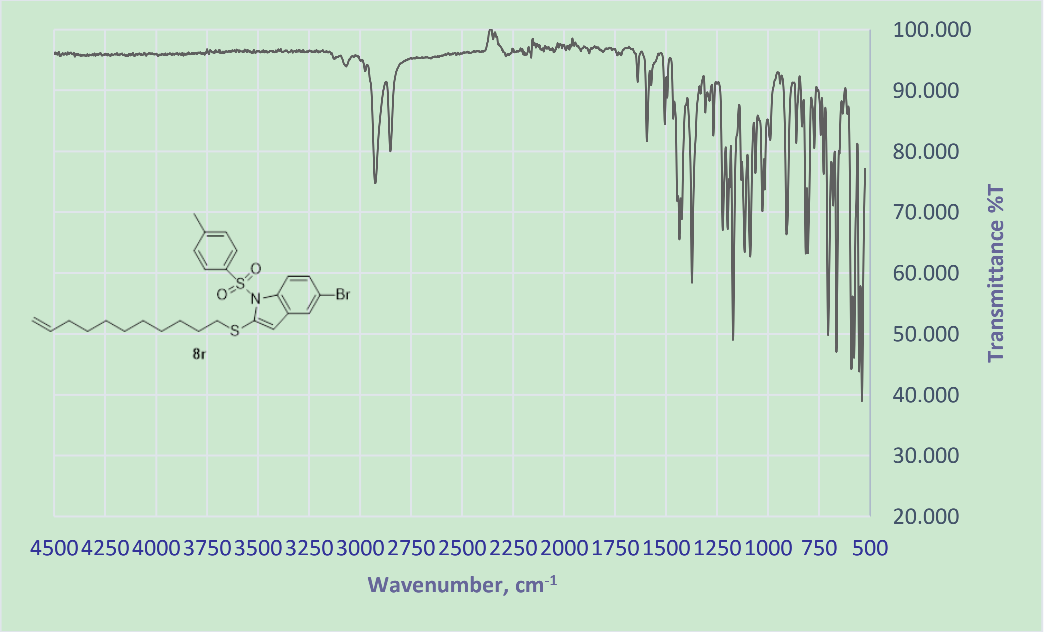
Br
8r
Transmittance %T
Wavenumber, cm-1

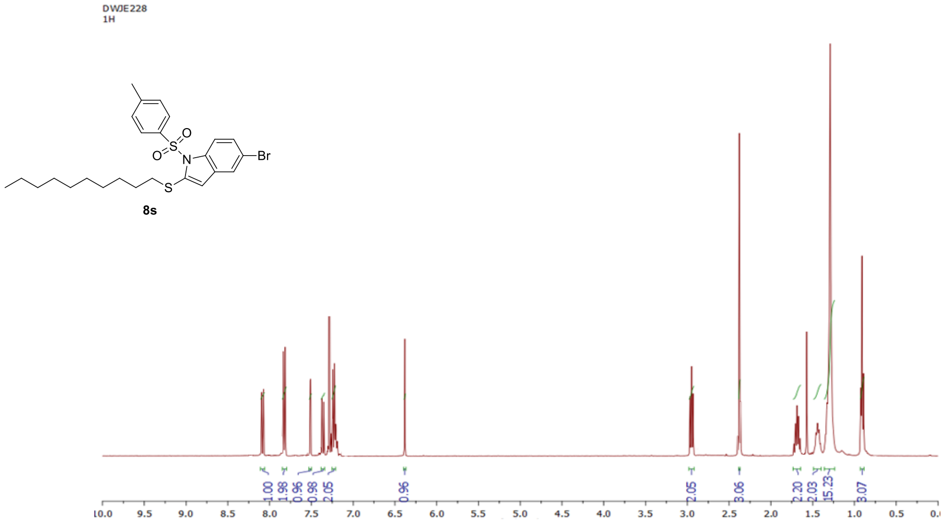
DWJE228
1H
8s
Br
1.00
1.98
0.96
0.98
2.05
0.96
2.05
3.06
2.20
2.03
15.23
3.07
10.0 9.5 9.0 8.5 8.0 7.5 7.0 6.5 6.0 5.5 5.0 4.5 4.0 3.5 3.0 2.5 2.0 1.5 1.0 0.5

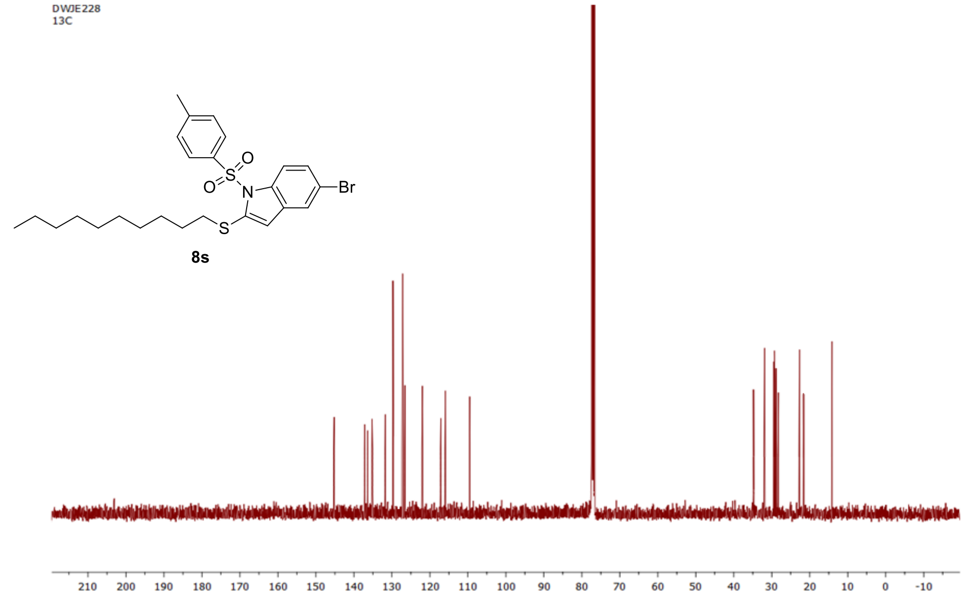
DWJE228
13C
8s
Br
210 200 190 180 170 160 150 140 130 120 110 100 90 80 70 60 50 40 30 20 10 0 -10

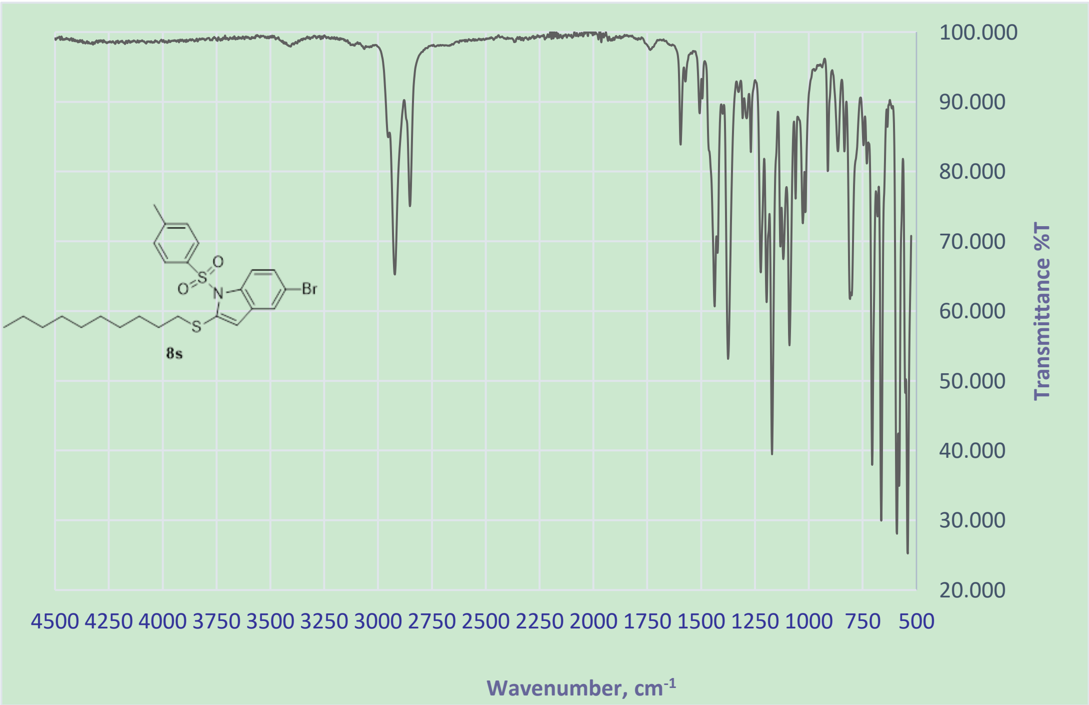
8s
Br
100.000
90.000
80.000
70.000
60.000
50.000
40.000
30.000
20.000
Transmittance %T
4500 4250 4000 3750 3500 3250 3000 2750 2500 2250 2000 1750 1500 1250 1000 750 500
Wavenumber, cm-1

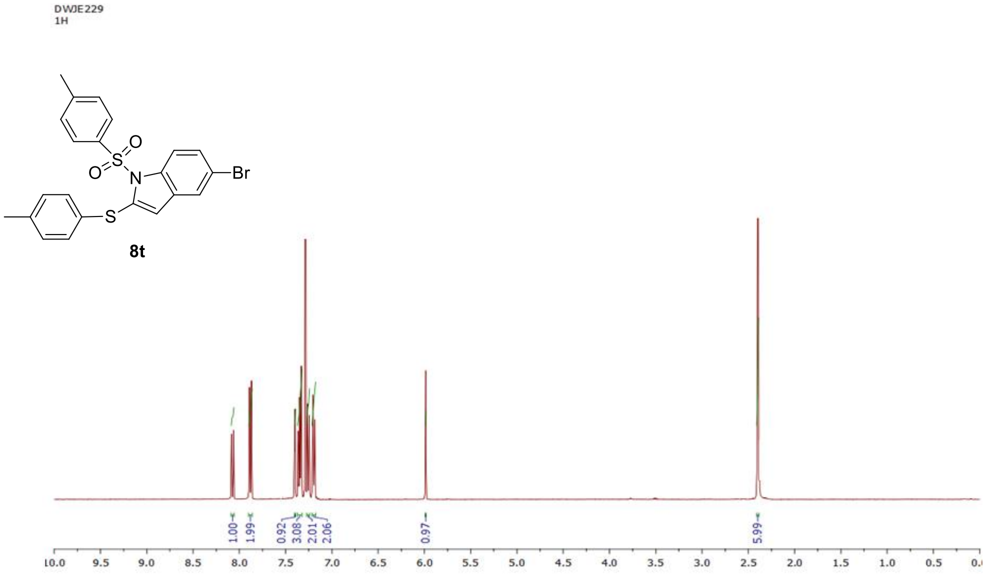
DWJE229
1H
8t
1.00
1.99
0.92
3.08
2.01
2.06
0.97
5.99

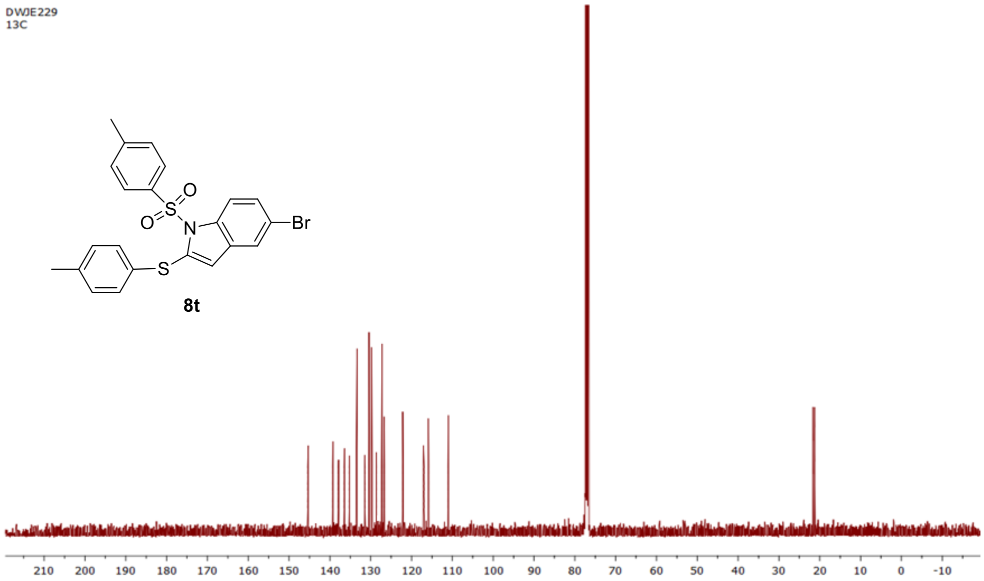
DWJE229
13C
8t

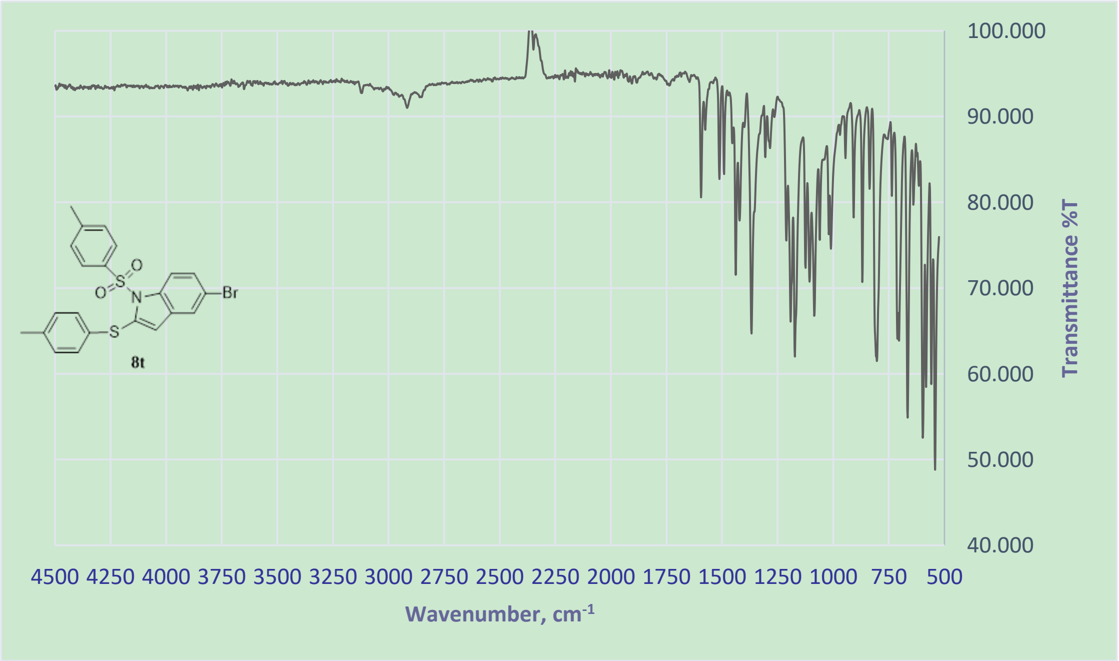
8t
Transmittance %T
Wavenumber, cm-1

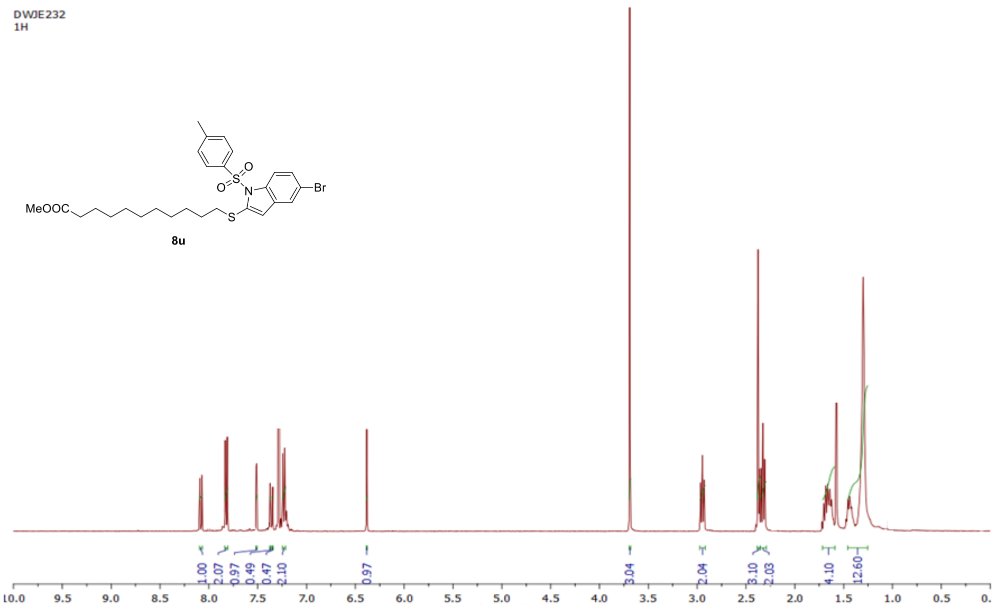
DWJE232
1H
MeOOC
S
N
O
Br
8u
1.00
2.07
0.97
0.49
0.47
2.10
0.97
3.04
2.04
3.10
2.03
4.10
12.60

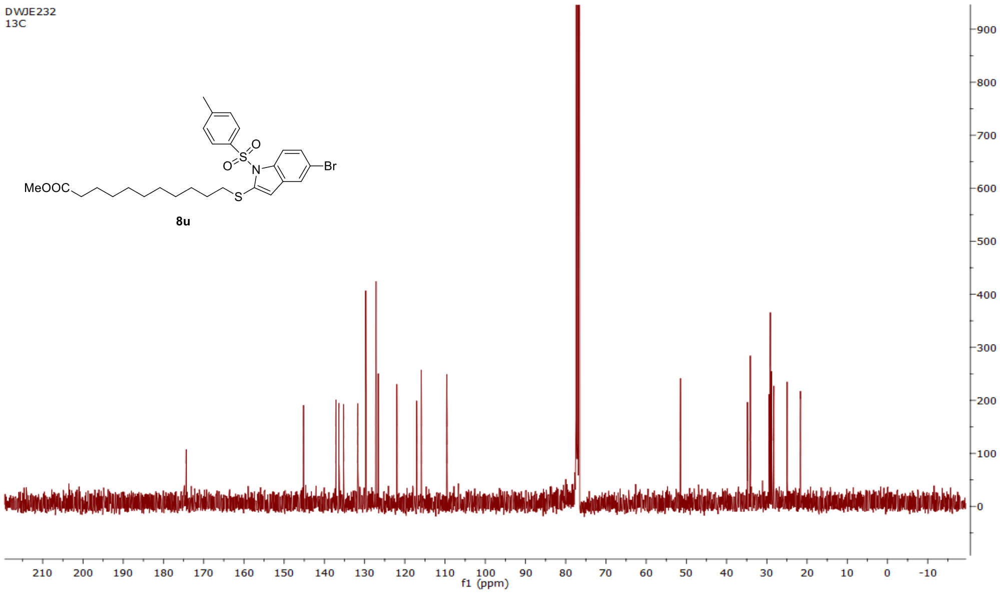
DWJE232
13C
MeOOC
S
N
O
Br
8u
f1 (ppm)

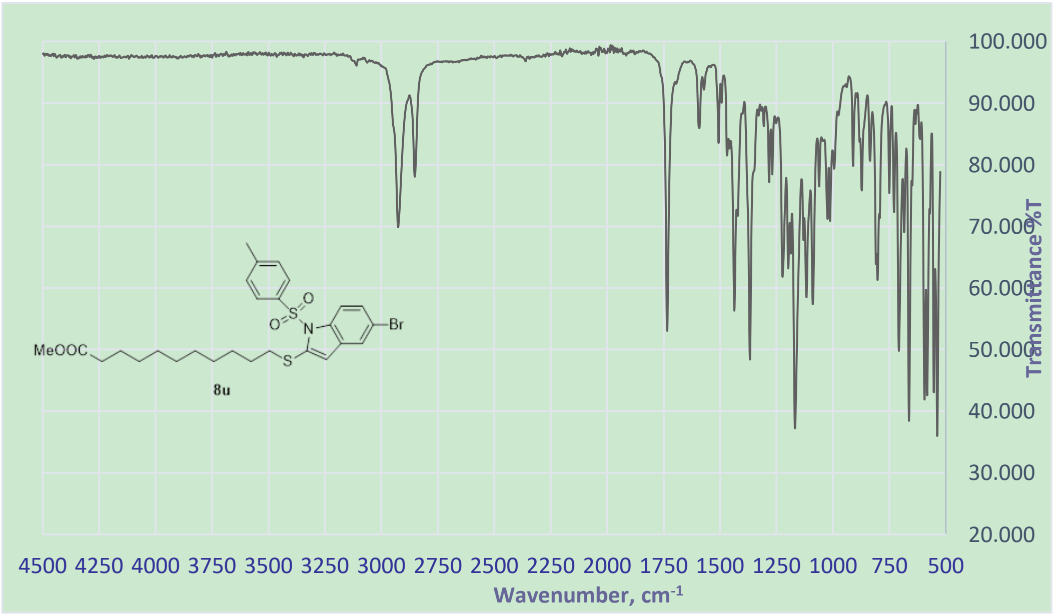
MeOOC
S
N
O
Br
8u
Transmittance %T
Wavenumber, cm-1

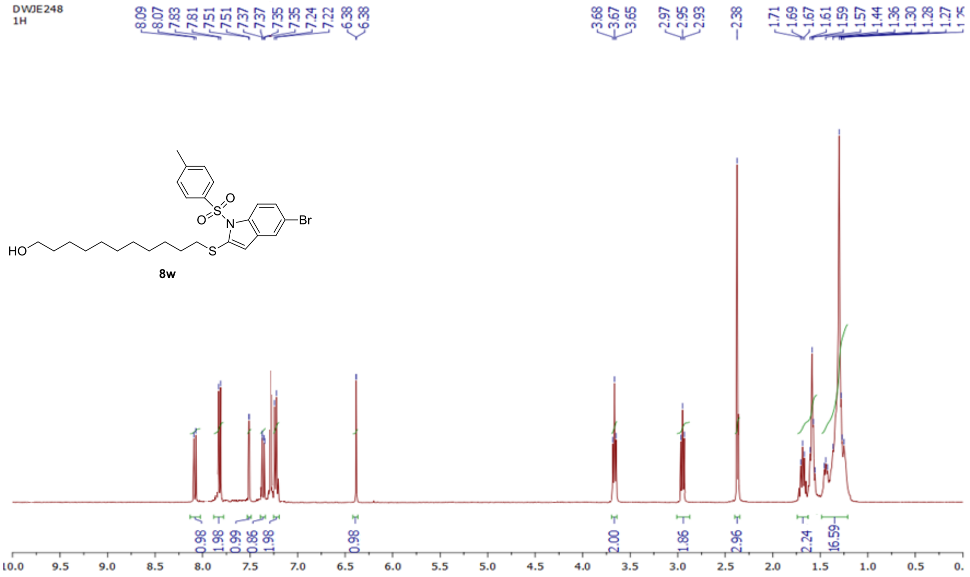
DWJE248
1H
8w
HO
Br

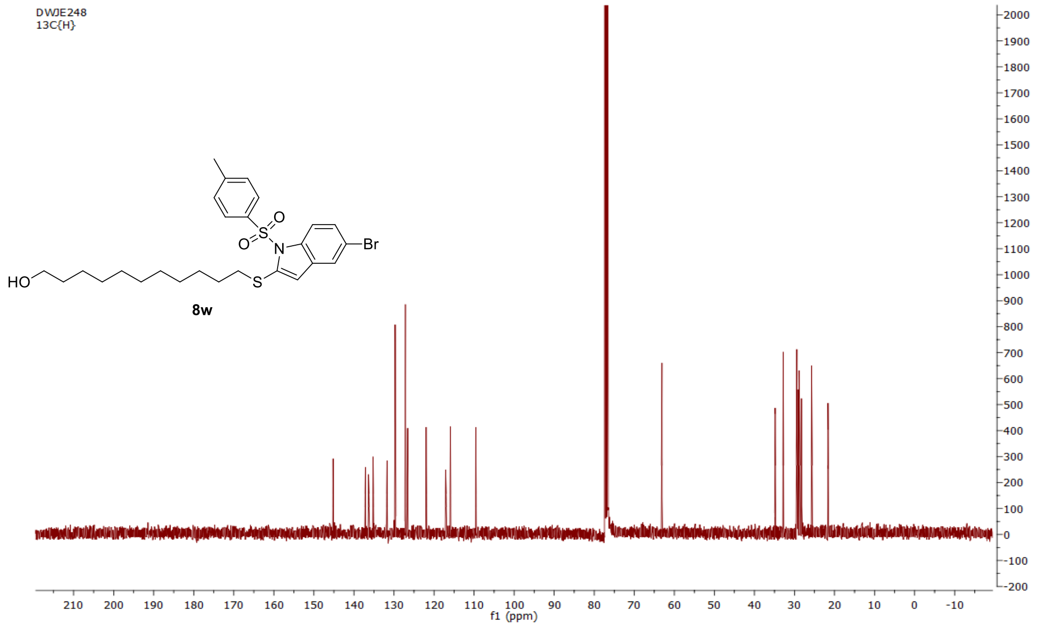
DWJE248
13C{H}
8w
f1 (ppm)

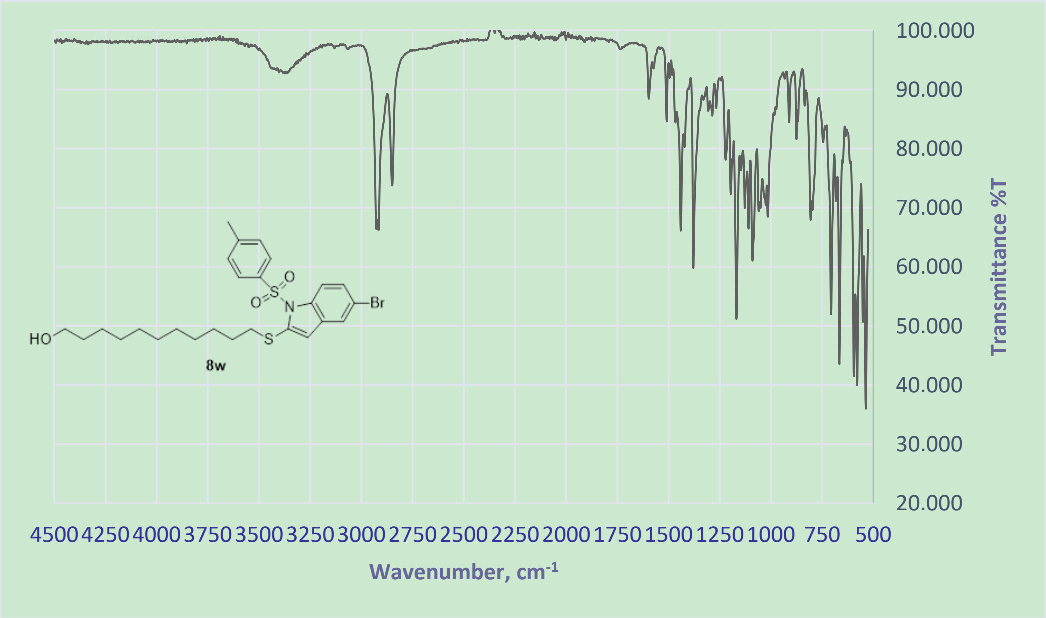
8w
Transmittance %T
Wavenumber, cm-1

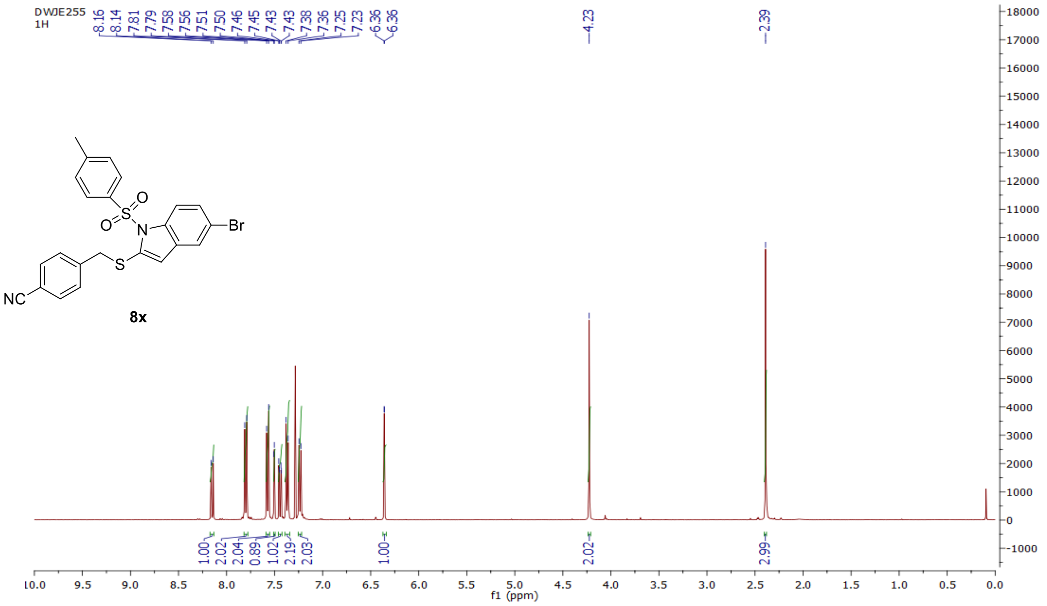
DWJE255
1H
8.16
8.14
7.81
7.79
7.58
7.56
7.51
7.50
7.46
7.45
7.43
7.43
7.38
7.36
7.25
7.23
6.36
6.36
4.23
2.39
Br
NC
S
N
O
8x
1.00
2.02
2.04
0.89
1.02
2.19
2.03
1.00
2.02
2.99
f1 (ppm)

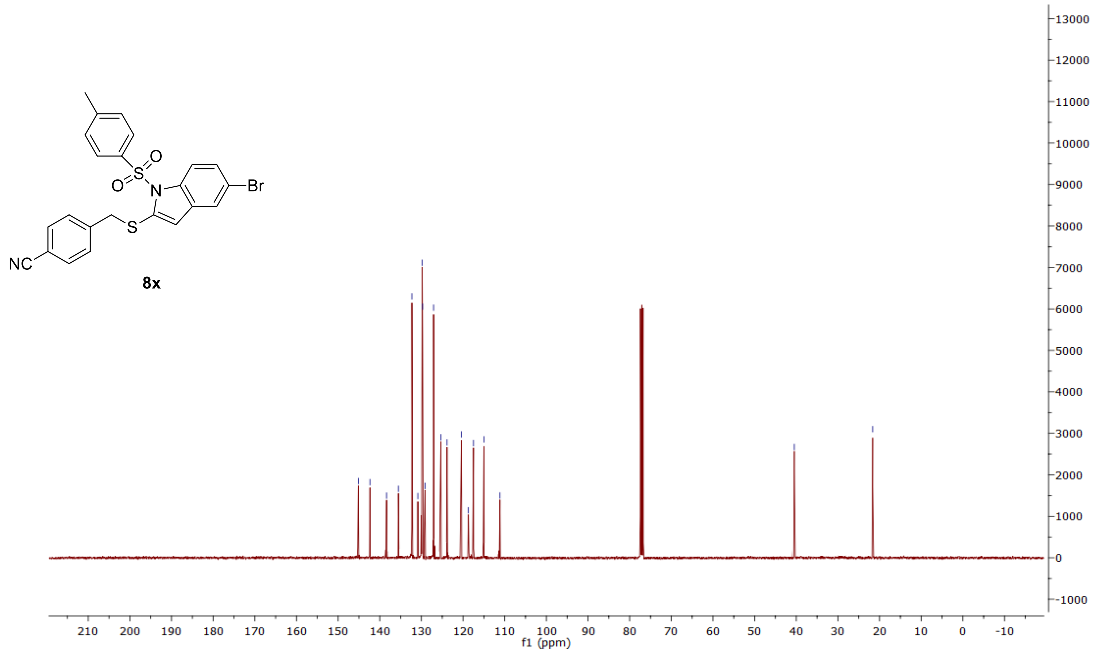
Br
NC
S
N
O
8x
f1 (ppm)

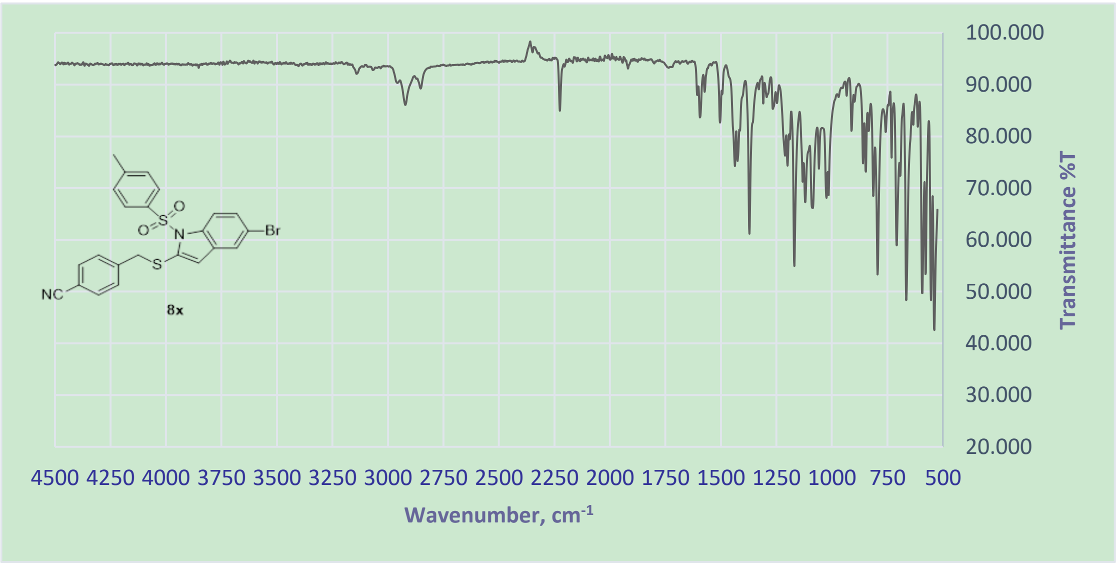
Br
NC
S
N
O
8x
Transmittance %T
Wavenumber, cm⁻¹

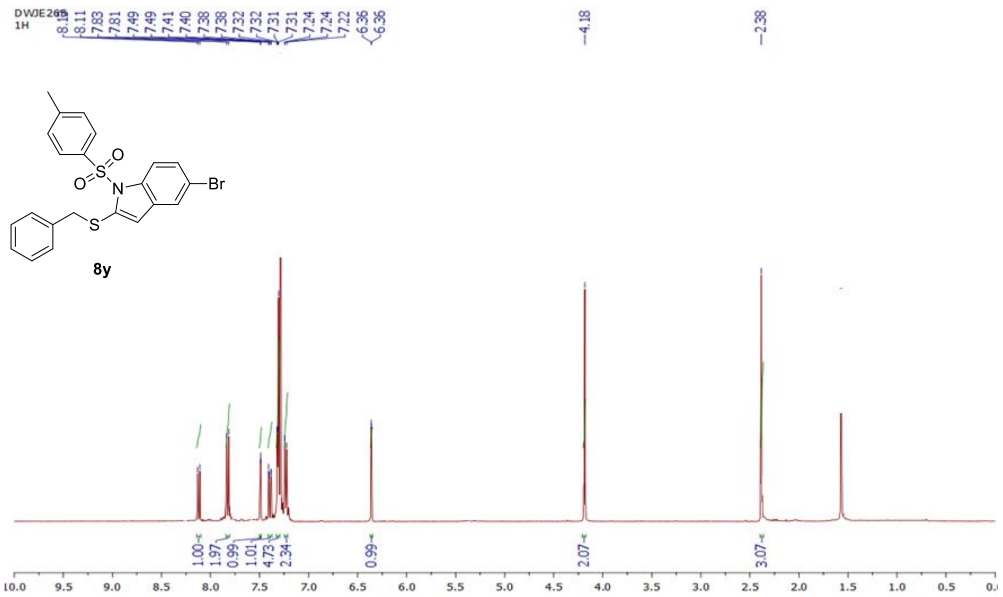
DWJE265
1H
8.13
8.11
7.83
7.81
7.49
7.49
7.41
7.40
7.38
7.38
7.32
7.32
7.31
7.31
7.31
7.24
7.24
7.22
6.36
6.36
4.18
2.38
8y
1.00
1.97
0.99
1.01
4.73
2.34
0.99
2.07
3.07

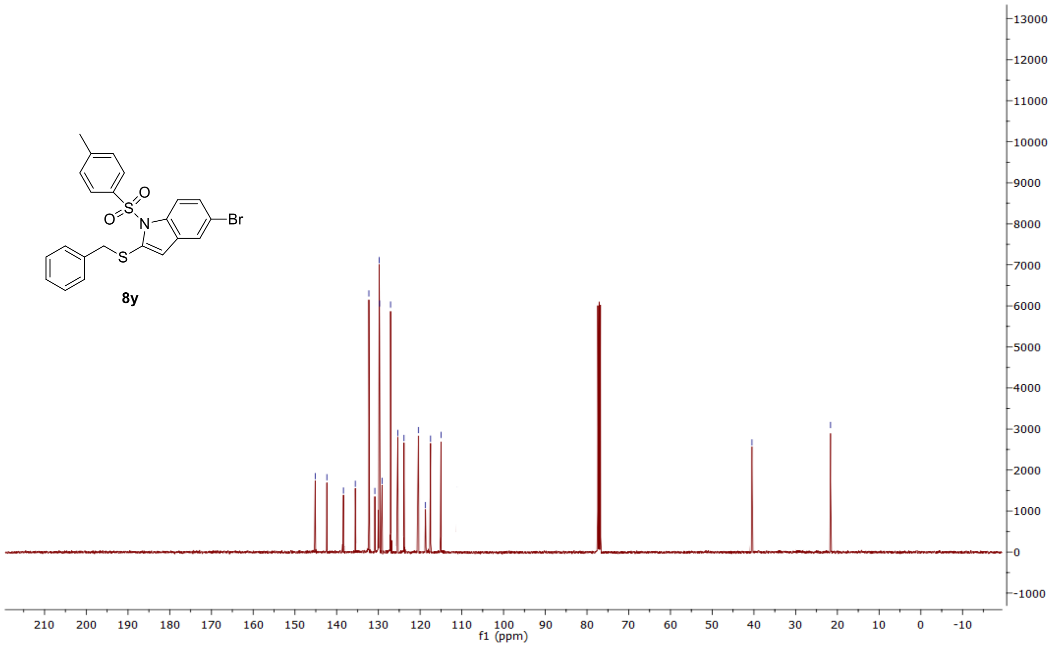
8y
f1 (ppm)

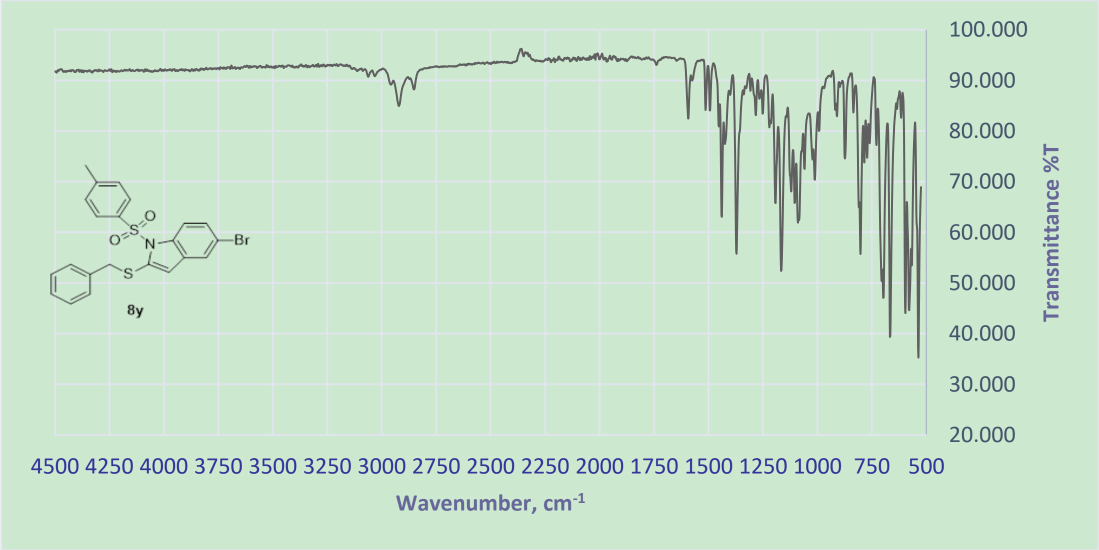
8y
Transmittance %T
Wavenumber, cm-1

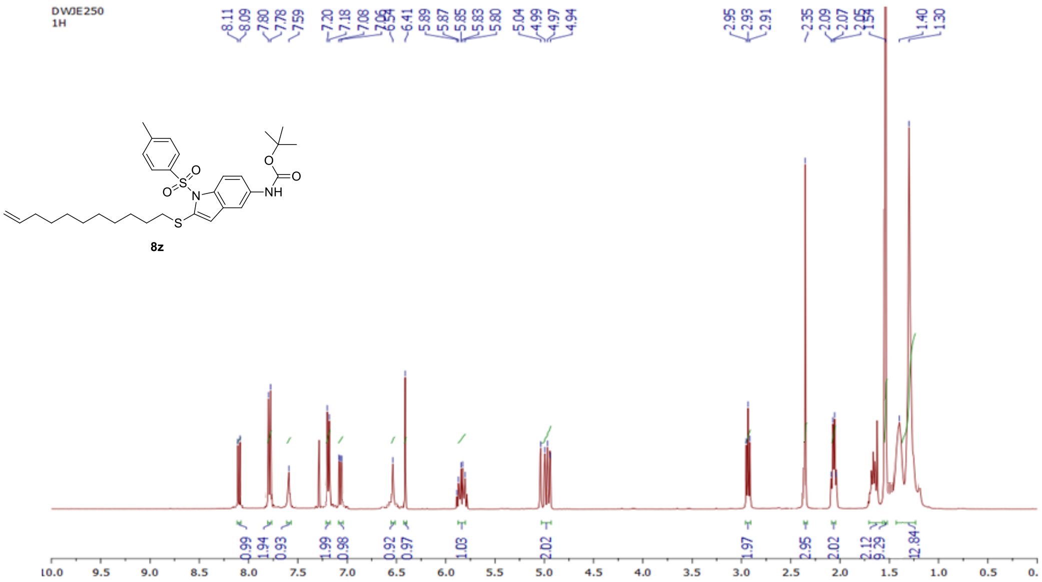
DWJE250
1H
8z
NH
O
S
N

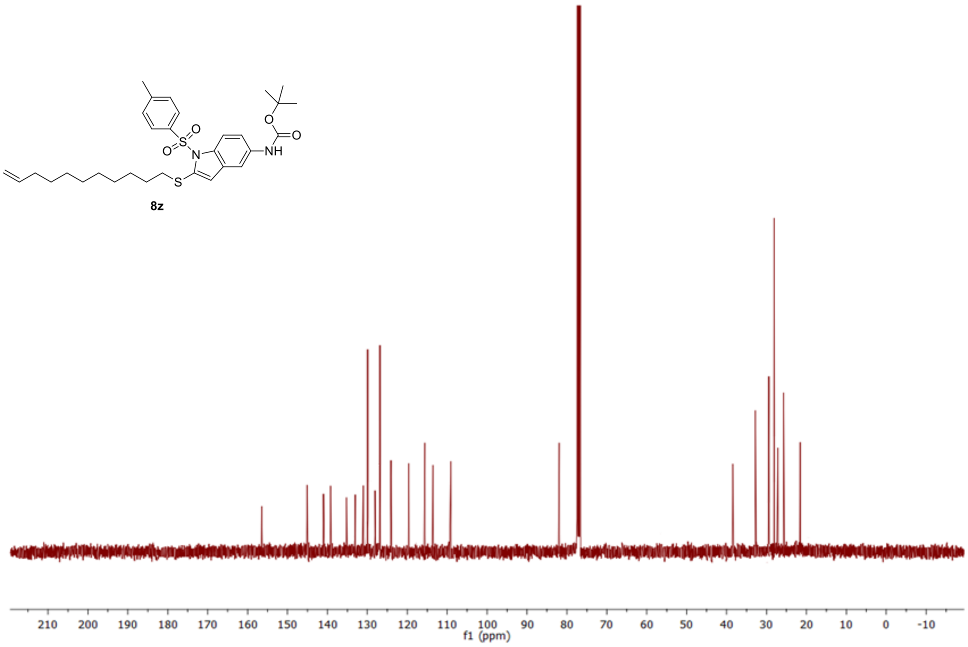
8z
f1 (ppm)

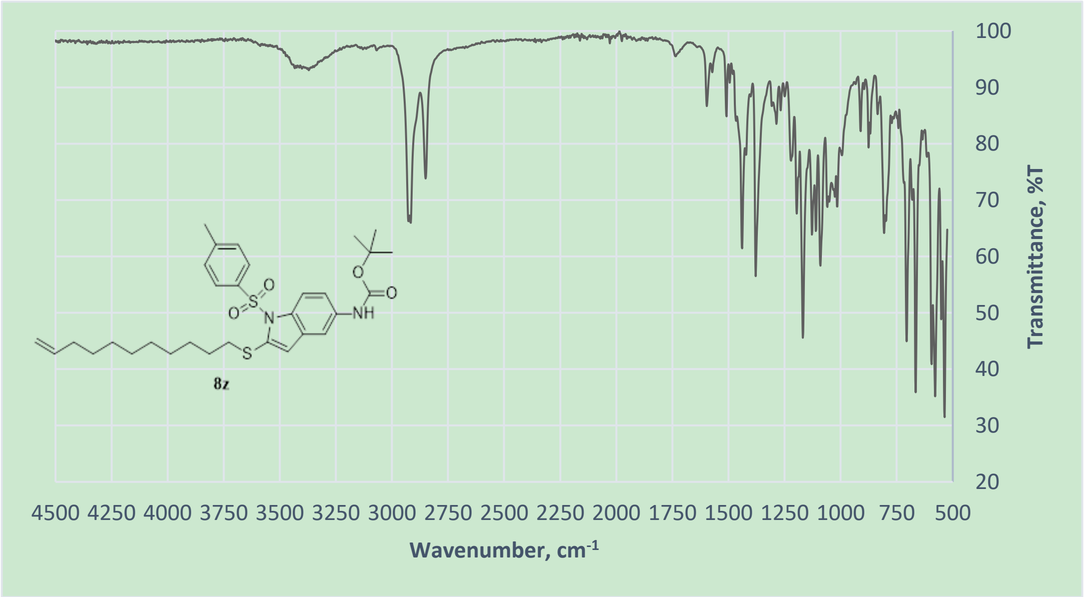
8z
Transmittance, %T
Wavenumber, cm-1

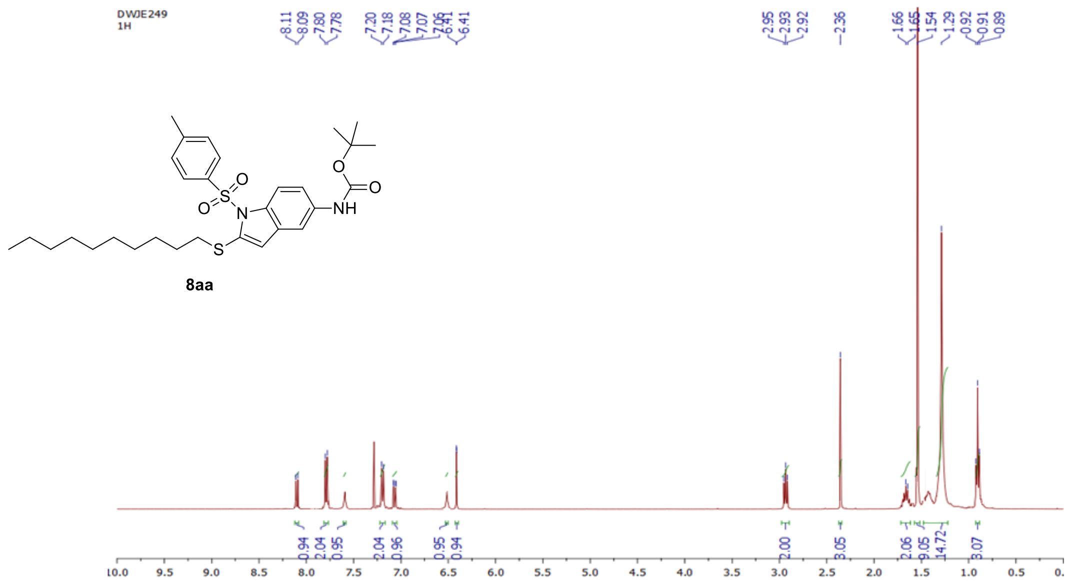

DWJE249
1H
NH
O
N
S
8aa

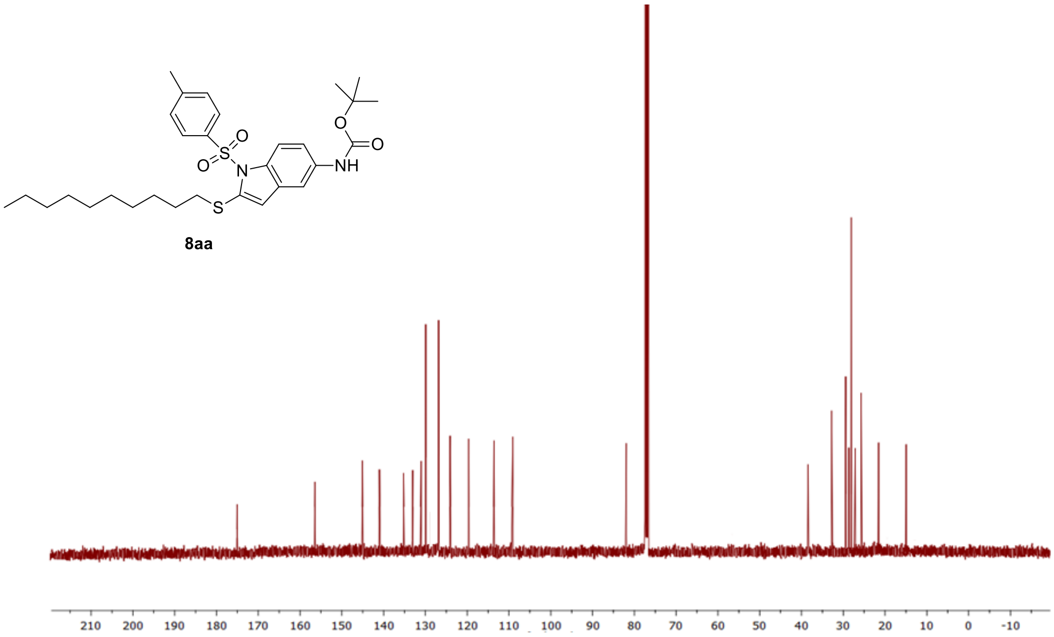

NH
8aa

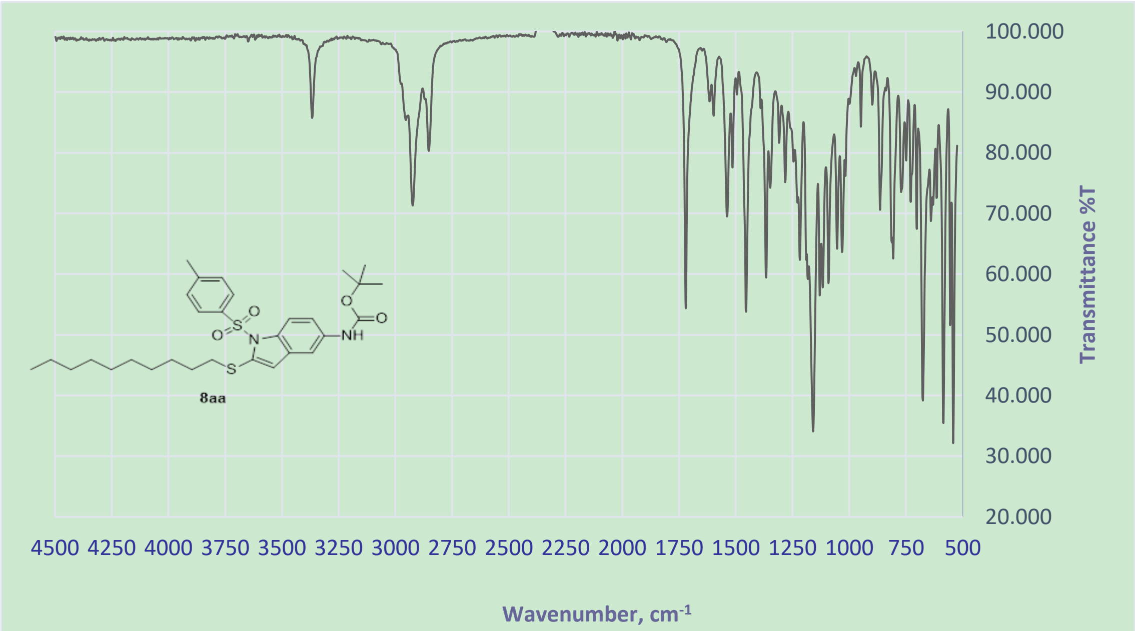

100.000
90.000
80.000
70.000
60.000
50.000
40.000
30.000
20.000
Transmittance %T
4500 4250 4000 3750 3500 3250 3000 2750 2500 2250 2000 1750 1500 1250 1000 750 500
Wavenumber, cm-1
8aa

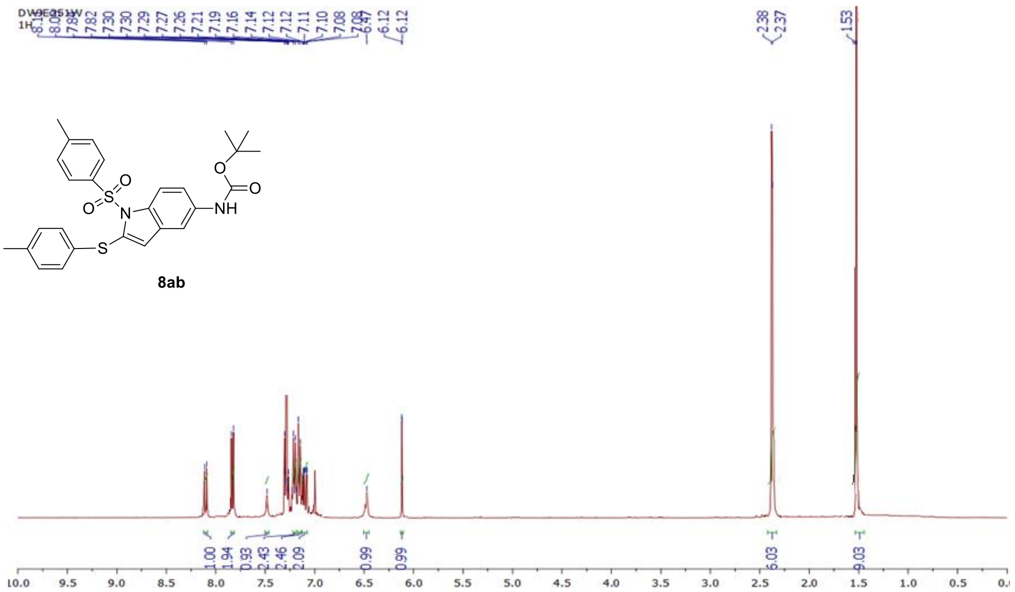
1H
8ab
2.38
2.37
1.53
1.00
1.94
0.93
2.43
2.46
2.09
0.99
0.99
6.03
9.03
10.0 9.5 9.0 8.5 8.0 7.5 7.0 6.5 6.0 5.5 5.0 4.5 4.0 3.5 3.0 2.5 2.0 1.5 1.0 0.5

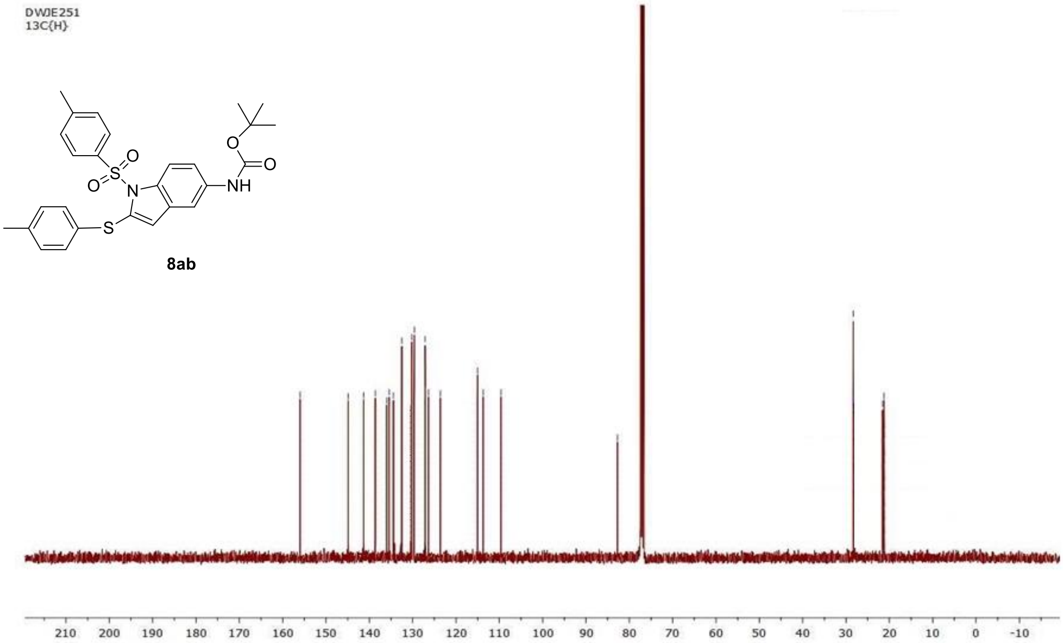
DWJE251
13C{H}
8ab
210 200 190 180 170 160 150 140 130 120 110 100 90 80 70 60 50 40 30 20 10 0 -10

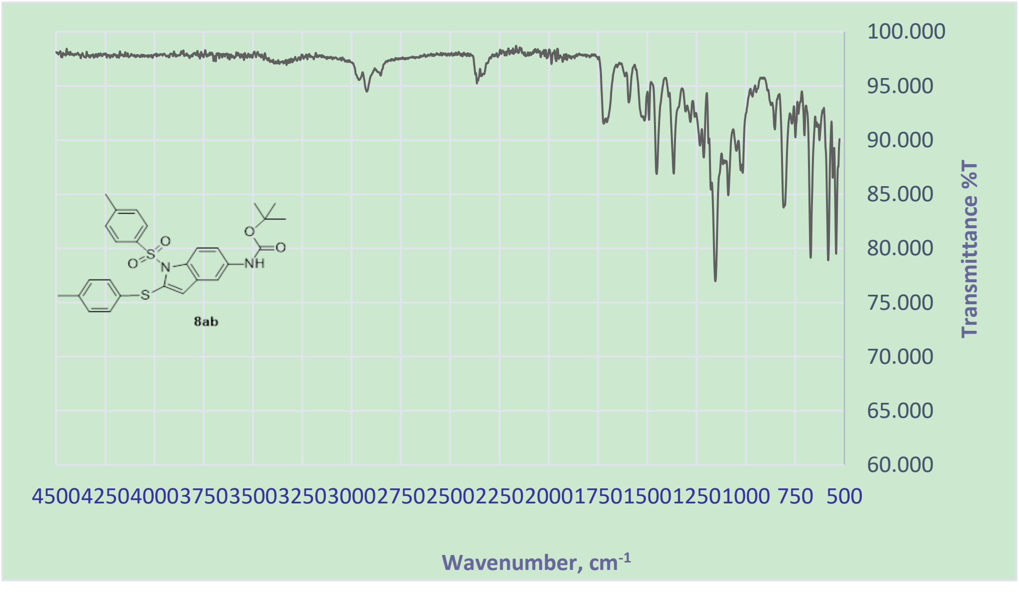
8ab
100.000
95.000
90.000
85.000
80.000
75.000
70.000
65.000
60.000
Transmittance %T
4500 4250 4000 3750 3500 3250 3000 2750 2500 2250 2000 1750 1500 1250 1000 750 500
Wavenumber, cm-1

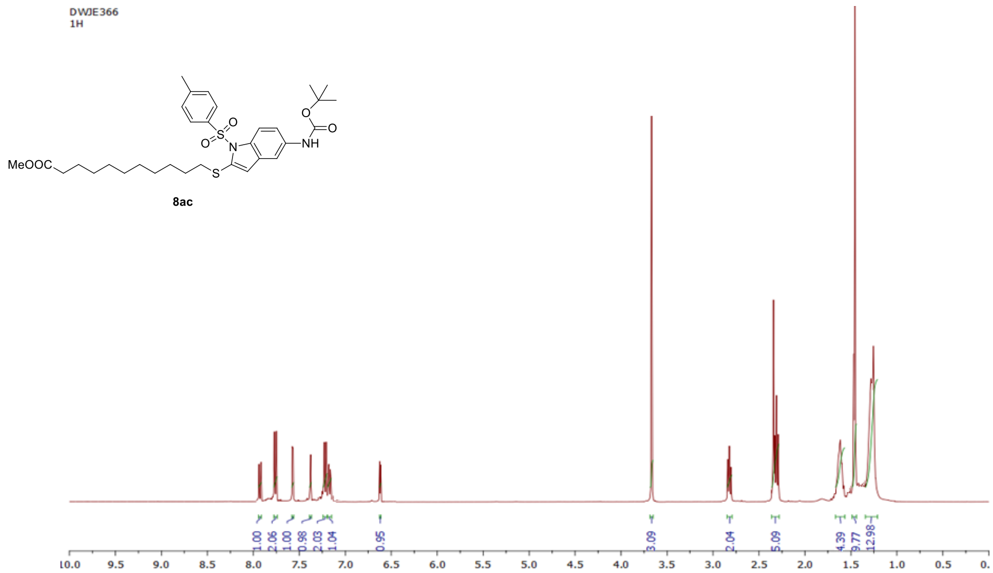
DWJE366
1H
MeOOC
8ac
1.00
2.06
1.00
0.98
2.03
1.04
0.95
3.09
2.04
5.09
4.39
9.77
12.98

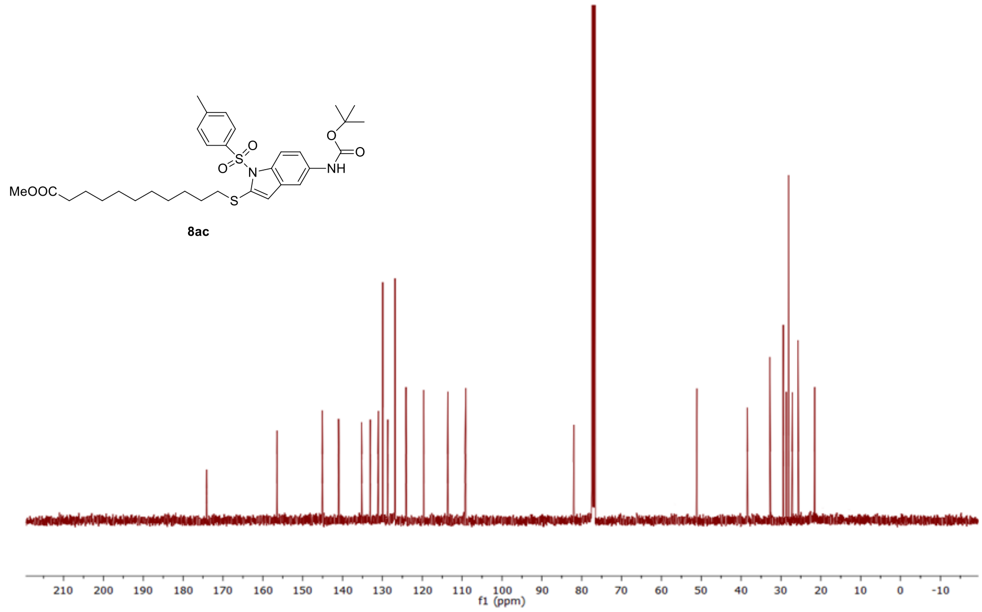
MeOOC
8ac
f1 (ppm)

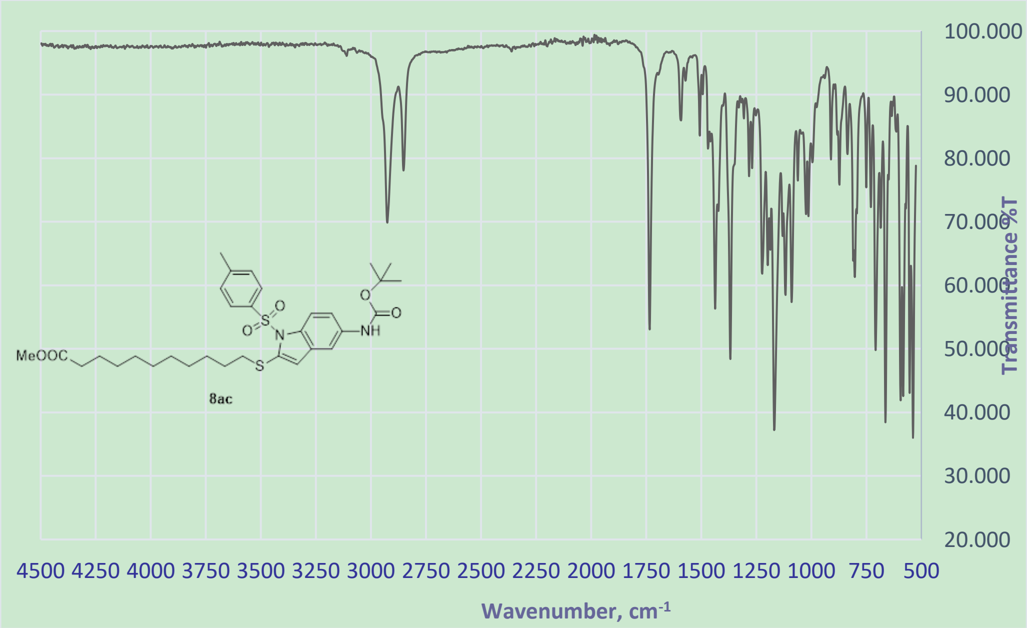
MeOOC
8ac
Transmittance %T
Wavenumber, cm-1

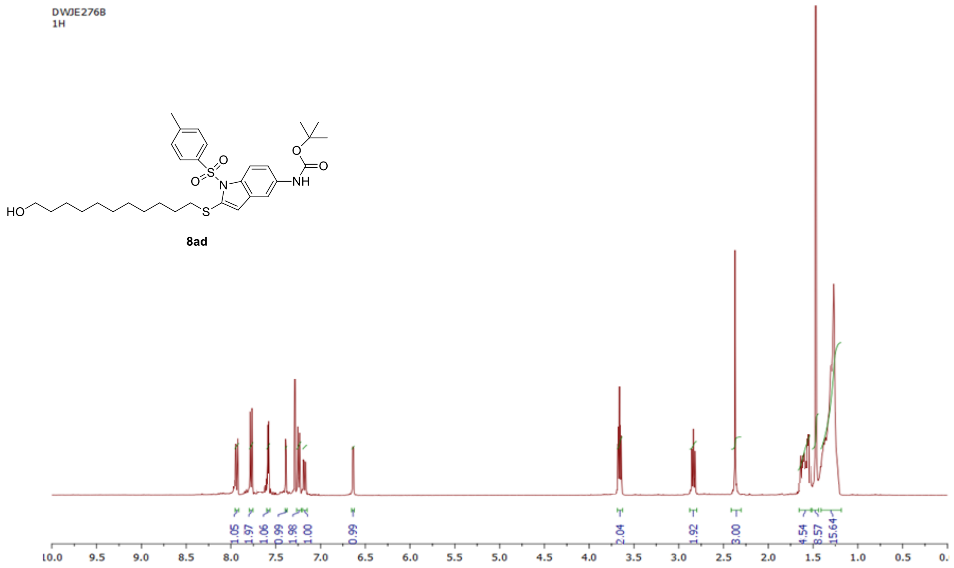
DWJE276B
1H
HO
S
N
O
S
O
NH
O
O
8ad
1.05
1.97
1.06
0.99
1.98
1.00
0.99
2.04
1.92
3.00
4.54
8.57
15.64

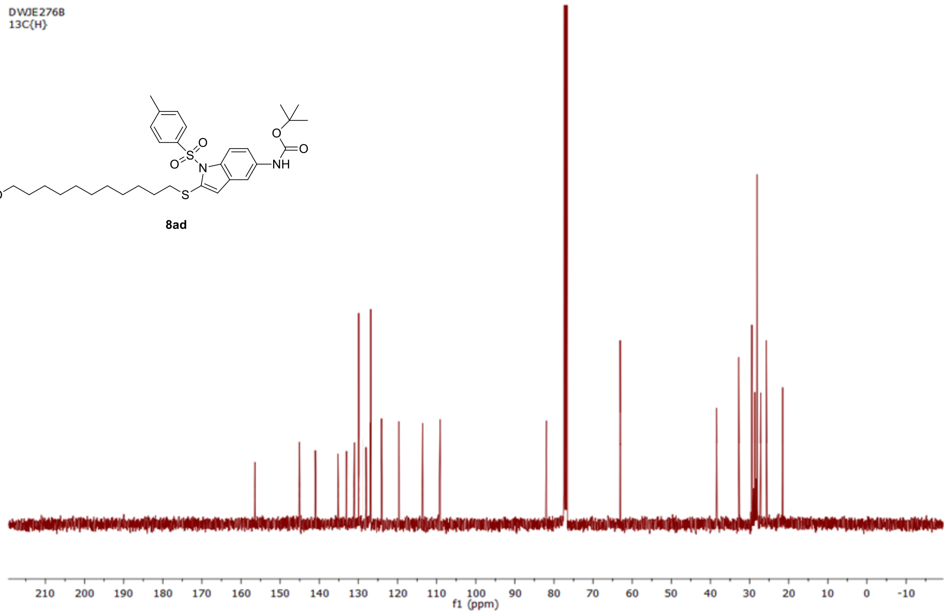
DWJE276B
13C{H}
HO
S
N
O
S
O
NH
O
O
8ad
f1 (ppm)

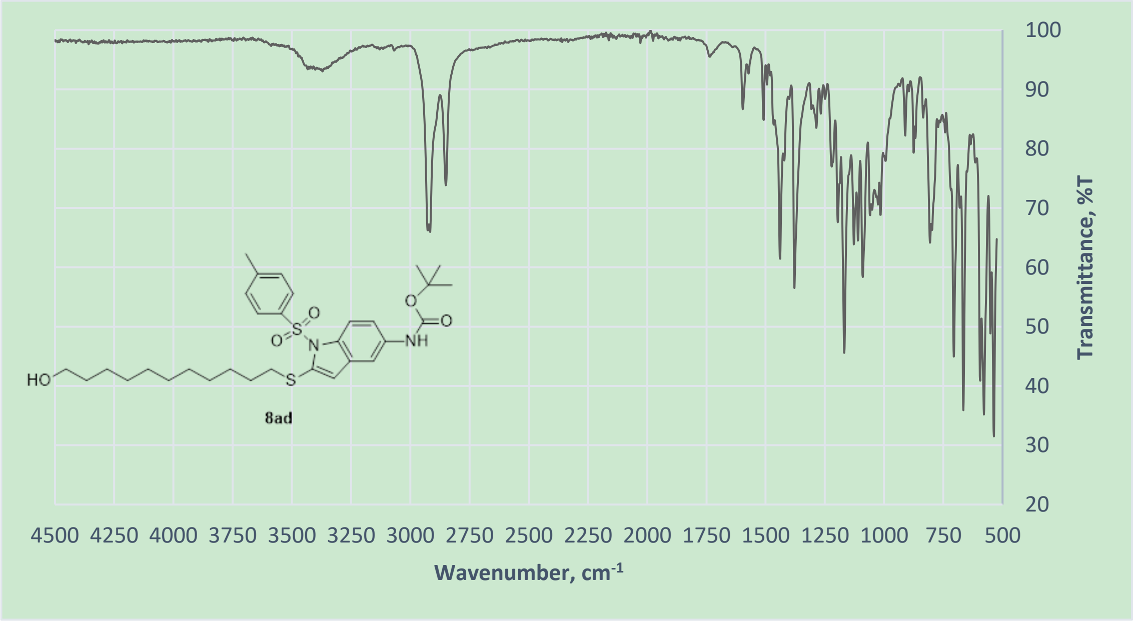
HO
S
N
O
S
O
NH
O
O
8ad
Transmittance, %T
Wavenumber, cm-1

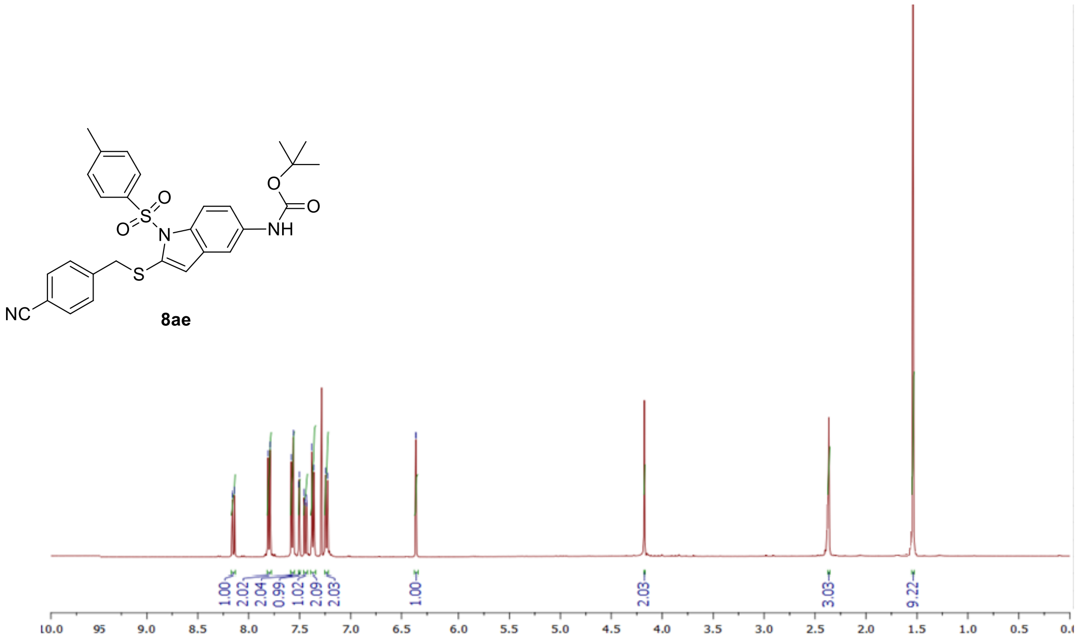
NC
S
N
O
S
O
NH
O
O
8ae
1.00
2.02
2.04
0.99
1.02
2.09
2.03
1.00
2.03
3.03
9.22
10.0 95 9.0 8.5 8.0 7.5 7.0 6.5 6.0 5.5 5.0 4.5 4.0 3.5 3.0 2.5 2.0 1.5 1.0 0.5 0.0

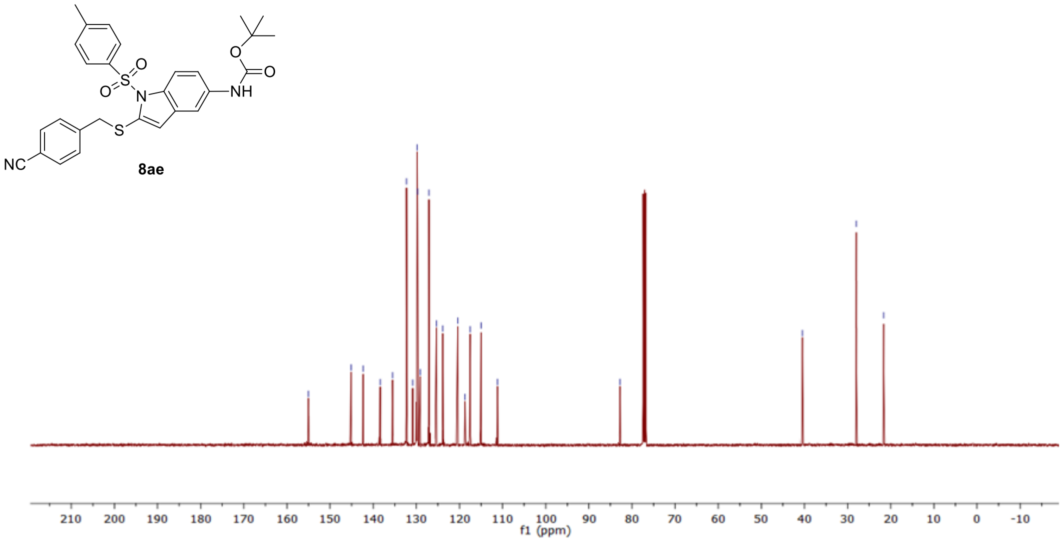
NC
S
N
O
S
O
NH
O
O
8ae
210 200 190 180 170 160 150 140 130 120 110 100 90 80 70 60 50 40 30 20 10 0 -10
f1 (ppm)

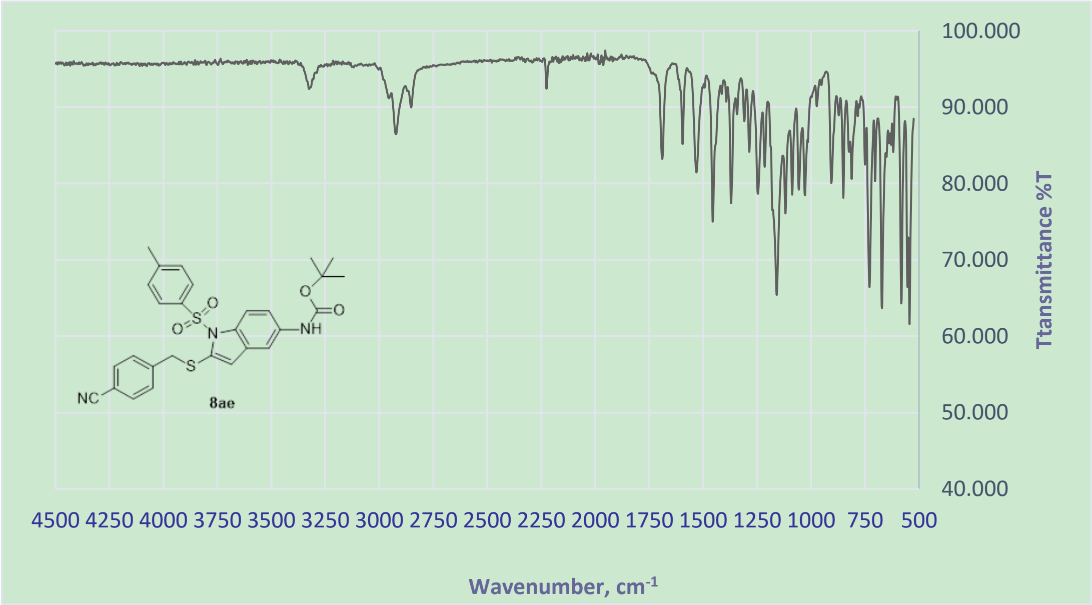
100.000
90.000
80.000
70.000
60.000
50.000
40.000
Ttansmittance %T
4500 4250 4000 3750 3500 3250 3000 2750 2500 2250 2000 1750 1500 1250 1000 750 500
Wavenumber, cm-1
NC
S
N
O
S
O
NH
O
O
8ae

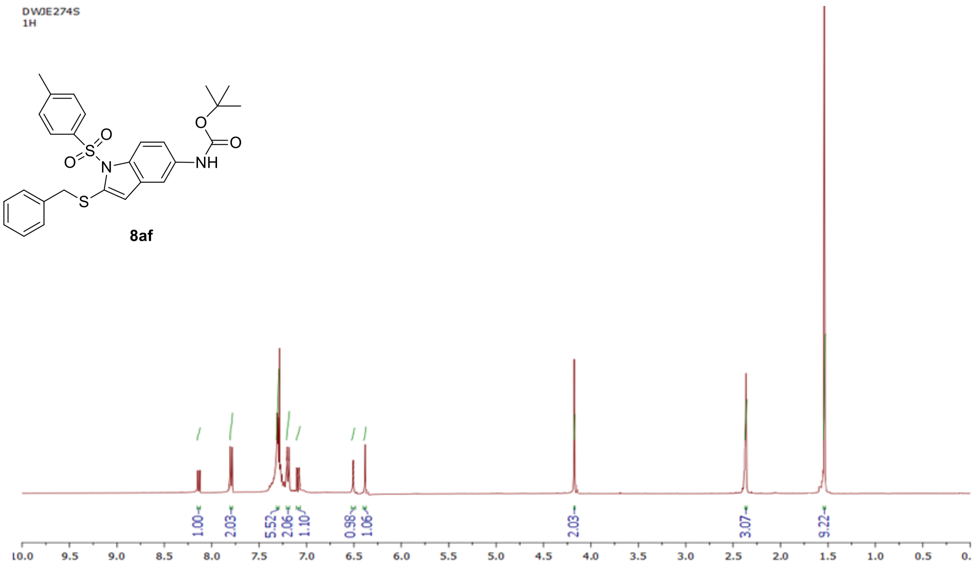
DWJE274S
1H
8af
1.00
2.03
5.52
2.06
1.10
0.98
1.06
2.03
3.07
9.22

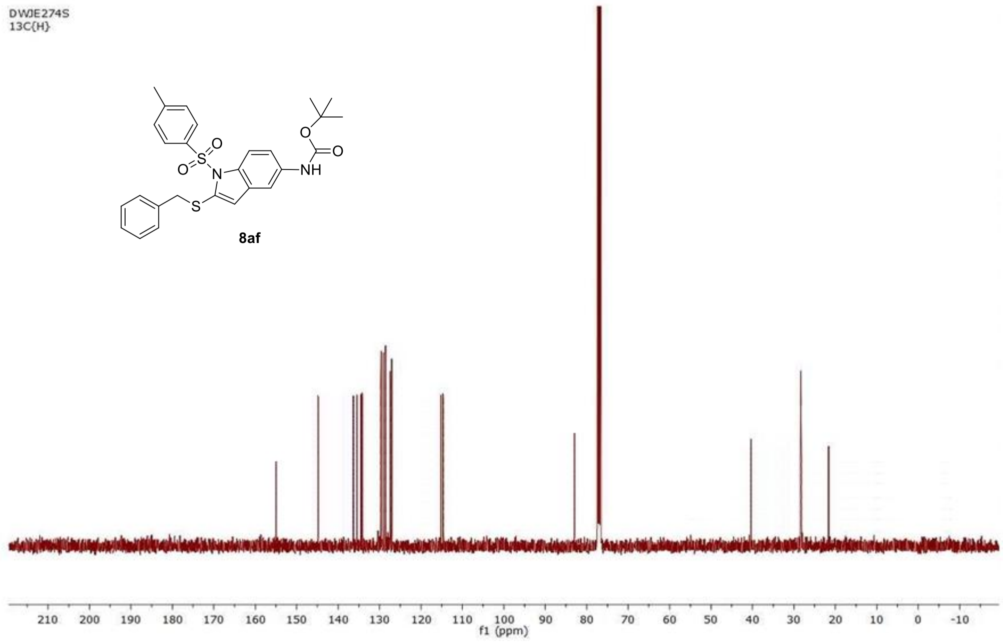
DWJE274S
13C{H}
8af
f1 (ppm)

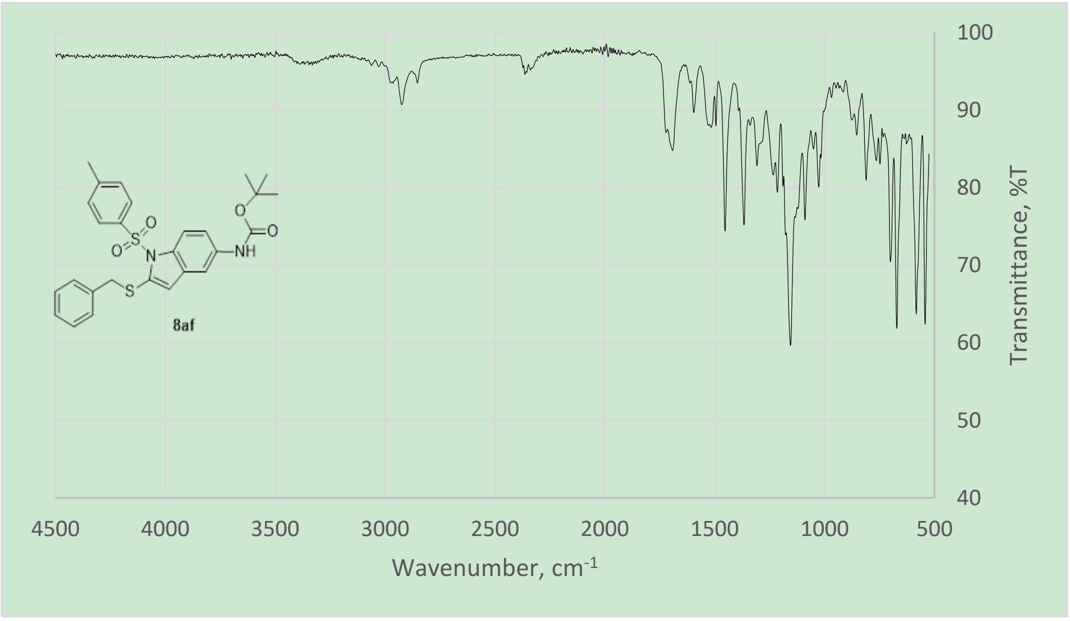
8af
Transmittance, %T
Wavenumber, cm-1

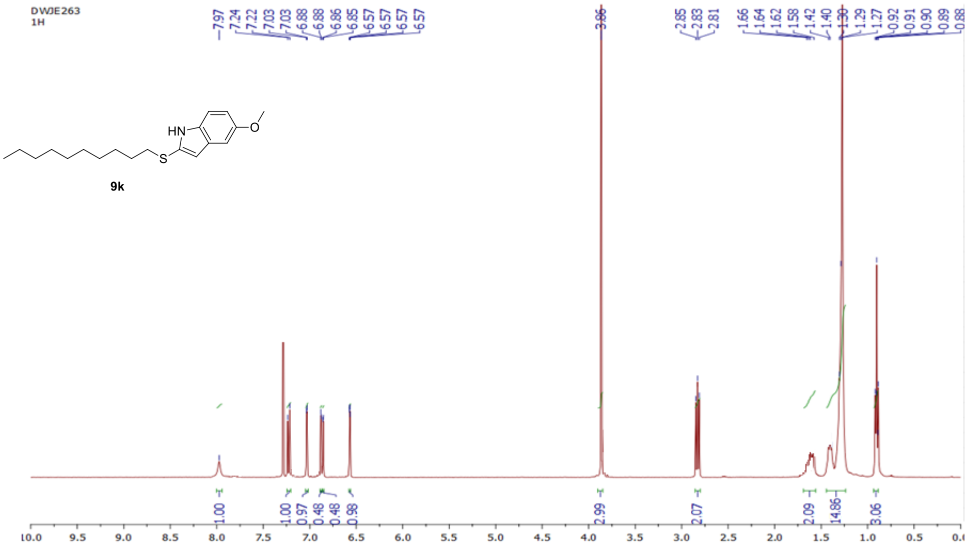
DWJE263
1H
HN
S
O
9k
7.97
7.24
7.22
7.03
7.03
6.88
6.88
6.86
6.85
6.57
6.57
6.57
6.57
3.86
2.85
2.83
2.81
1.66
1.64
1.62
1.58
1.42
1.40
1.30
1.29
1.27
0.92
0.91
0.90
0.89
0.88
1.00
1.00
0.97
0.48
0.48
0.98
2.99
2.07
2.09
14.86
3.06

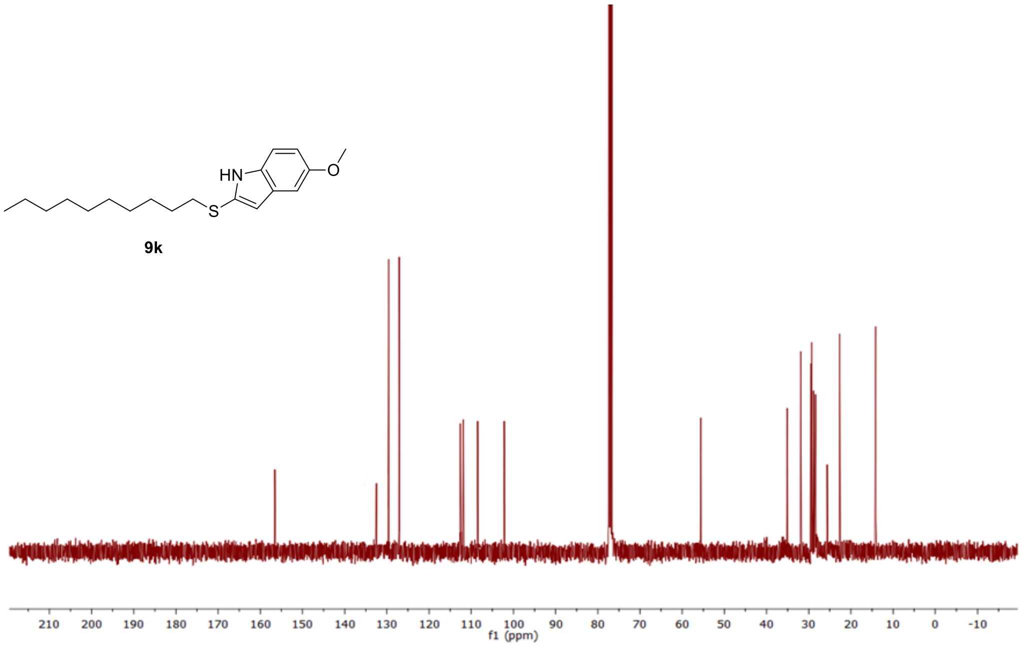
HN
S
O
9k
f1 (ppm)

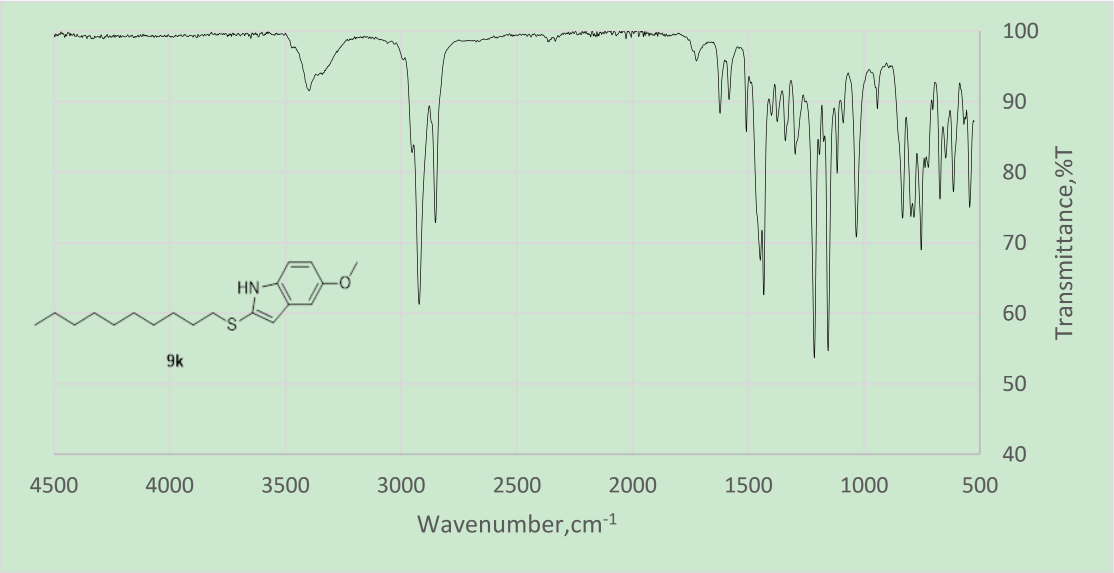
HN
S
O
9k
Transmittance,%T
Wavenumber,cm-1
